# Two C-terminal isoforms of *Aplysia* tachykinin–related peptide receptors exhibit phosphorylation-dependent and phosphorylation-independent desensitization mechanisms

**DOI:** 10.1016/j.jbc.2024.107556

**Published:** 2024-07-11

**Authors:** Rui-ting Mao, Shi-qi Guo, Guo Zhang, Ya-dong Li, Ju-ping Xu, Hui-ying Wang, Ping Fu, Cui-ping Liu, Shao-qian Wu, Ping Chen, Yu-shuo Mei, Qing-chun Jin, Cheng-yi Liu, Yan-chu-fei Zhang, Xue-ying Ding, Wei-jia Liu, Elena V. Romanova, Hai-bo Zhou, Elizabeth C. Cropper, James W. Checco, Jonathan V. Sweedler, Jian Jing

**Affiliations:** 1 Department of Neurology and Medical Psychology, Nanjing Drum Tower Hospital, State Key Laboratory of Pharmaceutical Biotechnology, Institute for Brain Sciences, Chinese Academy of Medical Sciences Research Unit of Extracellular RNA, Jiangsu Engineering Research Center for MicroRNA Biology and Biotechnology, Chemistry and Biomedicine Innovation Center, School of Life Sciences, Nanjing University, Nanjing, Jiangsu, China; 2 Department of Chemistry and the Beckman Institute for Advanced Science and Technology, University of Illinois at Urbana-Champaign, Urbana, Illinois, USA; 3 School of Electronic Science and Engineering, Nanjing University, Nanjing, Jiangsu, China; 4 Peng Cheng Laboratory, Shenzhen, China; 5 Department of Neuroscience and Friedman Brain Institute, Icahn School of Medicine at Mount Sinai, New York, New York, USA; 6 Department of Chemistry, University of Nebraska-Lincoln, Lincoln, Nebraska, USA; 7 The Nebraska Center for Integrated Biomolecular Communication (NCIBC), University of Nebraska-Lincoln, Lincoln, Nebraska, USA

**Keywords:** G protein-coupled receptor (GPCR), neuropeptide, desensitization, signal transduction, ligand–receptor interaction, tachykinin, *Aplysia*

## Abstract

Diversity, a hallmark of G protein-coupled receptor (GPCR) signaling, partly stems from alternative splicing of a single gene generating more than one isoform for a receptor. Additionally, receptor responses to ligands can be attenuated by desensitization upon prolonged or repeated ligand exposure. Both phenomena have been demonstrated and exemplified by the deuterostome tachykinin signaling system, although the role of phosphorylation in desensitization remains a subject of debate. Here, we describe the signaling system for tachykinin-related peptides (TKRPs) in a protostome, mollusk *Aplysia*. We cloned the *Aplysia* TKRP precursor, which encodes three TKRPs (apTKRP-1, apTKRP-2a, and apTKRP-2b) containing the FXGXR-amide motif. *In situ* hybridization and immunohistochemistry showed predominant expression of TKRP mRNA and peptide in the cerebral ganglia. TKRPs and their posttranslational modifications were observed in extracts of central nervous system ganglia using mass spectrometry. We identified two *Aplysia* TKRP receptors (apTKRPRs), named apTKRPR-A and apTKRPR-B. These receptors are two isoforms generated through alternative splicing of the same gene and differ only in their intracellular C termini. Structure-activity relationship analysis of apTKRP-2b revealed that both C-terminal amidation and conserved residues of the ligand are critical for receptor activation. C-terminal truncates and mutants of apTKRPRs suggested that there is a C-terminal phosphorylation-independent desensitization for both receptors. Moreover, apTKRPR-B also exhibits phosphorylation-dependent desensitization through the phosphorylation of C-terminal Ser/Thr residues. This comprehensive characterization of the *Aplysia* TKRP signaling system underscores the evolutionary conservation of the TKRP and TK signaling systems, while highlighting the intricacies of receptor regulation through alternative splicing and differential desensitization mechanisms.

One fundamental property of neuropeptide signaling systems is their diversity, enabling the nervous system to modulate a variety of behaviors, such as feeding, locomotion, and reproduction (1, 2, 3, 4, 5, 6). This diversity is characterized by the existence of multiple peptide ligands, posttranslational modifications (PTMs) of ligands from a single precursor and possible actions of a ligand on its multiple G protein-coupled receptors (GPCRs) (5, 6, 7, 8, 9). In part, multiple GPCRs for a ligand could originate from GPCR isoforms derived from alternative splicing of receptor genes (10, 11, 12). Often, these receptor isoforms undergo varying degrees of desensitization, leading to attenuated responses to a ligand after prolonged or repeated exposure (13), further promoting diversity in the GPCR signaling.

Receptor desensitization is an important form of plasticity, regulating cellular and network activity across both ligand-gated ion channels and GPCRs (13, 14, 15). For GPCRs, this has been extensively studied in deuterostome tachykinin (TK) signaling systems, particularly regarding the substance P receptor in pain perception pathways in dorsal horn neurons (16), which underscores its functional significance. Substance P receptor desensitization (17) could reduce excessive scratching behavior (18). For molecular mechanisms, the TK receptor 1 gene was shown to encode neurokinin 1 receptor (NK1R), and through differential splicing, can generate long and truncated NK1R isoforms, termed NK1R-F and NK1R-T, respectively. These isoforms differ in their tissue distributions, response properties, and propensity for desensitization, with NK1R-F undergoing more significant desensitization compared to the desensitization-resistant NK1R-T (19, 20, 21, 22, 23). Desensitization of GPCRs typically involves receptor phosphorylation (a type of receptor PTMs) and arrestin signaling (24, 25, 26). Although substantial evidence supports phosphorylation-dependent desensitization of NK1R-F at Ser/Thr residues in its longer C terminus (27, 28, 29, 30, 31, 32), conflicting reports exist, suggesting that desensitization might also occur independent of phosphorylation (19, 21). Here, we study this issue in the ortholog of the TK signaling system in protostomes, that is, the tachykinin-related peptide (TKRP) system in mollusk, *Aplysia californica*. We show, for the first time, the presence of two isoforms of TKRP receptors in a protostome. Moreover, both of the isoforms undergo desensitization, with one of them appearing to show only a phosphorylation-independent mechanism, and the other showing both a phosphorylation-dependent and phosphorylation-independent component.

TKs in deuterostomes are a class of peptides characterized by a C-terminal FXGLM-amide motif (where X represents any amino acid). These peptides are implicated in pain, inflammation, depressive disorder, gut function, sensory processing, and more (4, 33). Similarly, TKRPs in protostomes feature an FXGXR-amide motif at the C terminus, with significant research conducted in *Drosophila* and other arthropods (4, 34). However, few studies have fully characterized TKRP signaling systems in the superphylum lophotrochozoa (*i.e.*, annelids, mollusks, and brachiopods), except for an annelid *Urechis unicinctus* (35, 36, 37, 38) and two mollusks: *Octopus vulgaris* (39, 40) and *Crassostrea gigas* (41), where only one precursor and one receptor have been identified in each species. For *Aplysia*, TKRP signaling is based solely on bioinformatic prediction of two *Aplysia* TKRPs (apTKRPs) (8), without experimental validation. More importantly, there is a lack of evidence for alternative splicing within the protostome TKRP signaling systems and limited data on desensitization, with a single report from the arthropod *Bombyx mori* (42). Another outstanding issue is that some protostome TKRPs may feature an N-terminal glutamine or glutamate, which could potentially be modified into pyroglutamic acid or pyroglutamate, affecting bioactivity (43, 44), though the impact of this modification on receptor activity remained undetermined.

*Aplysia* is an experimentally advantageous neurobiological system, widely used in studies of learning and memory (45, 46, 47, 48, 49), neural circuits (50, 51, 52, 53), and neuromodulation (54, 55, 56). Recent research has expanded to include numerous neuropeptides and their receptors, such as GdFFD/apALNR (57, 58, 59, 60), allatotropin-related peptides and their receptors (61, 62, 63), *Aplysia* leucokinin–like peptides and their receptors (64, 65), allatostatin C (AstC) and its receptors (66), *Aplysia* vasotocin and its receptors (67), elevenin and its receptors (68), and Wamide peptides and their receptors (69). In this report, we describe one TKRP precursor (apTKRP precursor) and two receptor isoforms (*Aplysia* tachykinin–related peptide receptors, apTKRPR-A and apTKRPR-B) in *Aplysia*, differing in the length of their intracellular C termini. We predict three TKRPs from the apTKRP precursor and identify them using mass spectrometry (MS). These peptides activate the apTKRPRs with high efficacy. Notably, the apTKRP featuring an N-terminal pyroglutamic acid exhibits the highest receptor activation potency. We also investigate the potential phosphorylation sites at the C-terminal Ser/Thr residues of the receptors, exploring their roles in desensitization. We find both similarities and distinctions in the desensitization mechanisms of the two isoforms, which may have implications for the studies in vertebrate TK signaling systems. The identification of alternative splicing and desensitization in the apTKRP signaling system underscores the conservation of the TKRP and TK signaling throughout evolution.

## Results

### Identifying the precursor and the TKRPs in *Aplysia*

**Figure 1:**
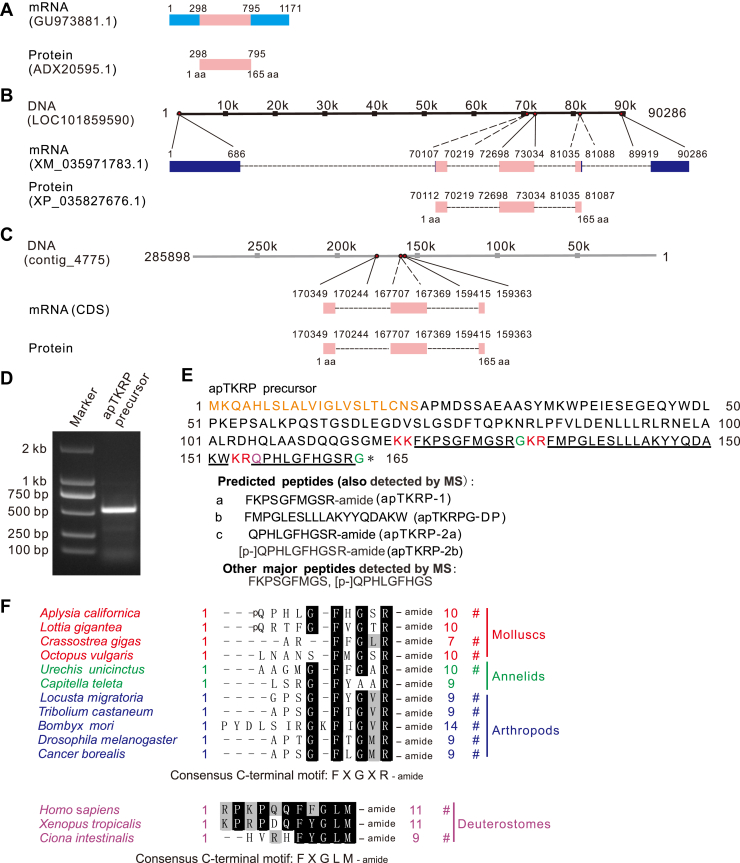
**Gene expression mapping and cloning of the *Aplysia* tachykinin–related peptide precursor.***A*, a mRNA from NCBI (GU973881.1), named *Aplysia californica* tachykinin-2 mRNA, complete cds, was submitted by Moroz *et al.* in 2016. It produces a protein, tachykinin-2 [*Aplysia californica*] (ADX20595.1). *B*, a gene from NCBI (LOC101859590) corresponds to an mRNA (XM_035971783.1), which produces an uncharacterized protein (XP_035827676.1), which is identical to ADX20595.1 in (*A*). *C*, a gene from the AplysiaTools (contig_4775) produces the same protein as in (*A* and *B*). Note that the nucleotide number on *top* starts from the *right* because it is a complementary DNA. *D*, a PCR product for apTKRP precursor gene with a length of ∼500 bp. Lane 1: DNA marker; Lane 2: the target gene. *E*, cleavage and processing of the apTKRP precursor. The apTKRP precursor consists of 165 amino acids and has a structure characteristic of a neuropeptide prohormone, having a signal peptide (*in orange*) and four predicted peptides: apTKRP-1, apTKRP-2a, apTKRP-2b, and apTKRPG-DP. KK and KR (*in red*) are potential basic cleavage sites. The predicted apTKRPs can undergo two posttranslational modifications: C-terminal amidation (the last Gly, shown in *green*) and N-terminal pyroglutamic acid cyclization (the first Gln, shown in *purple*). All the predicted peptides are also detected by mass spectrometry (MS). Two additional major peptides detected by MS in most or all ganglia are shown. See Table S3 and Fig. S4 for details on the MS data. *F*, comparison of selected TKRPs and TK peptides using BioEdit. All TKRPs have a conserved FXGXRamide motif, except for *Capitella teleta*. Similarly, all TK peptides have a conserved FXGLMamide motif. ∗ denotes the stop codon. # indicates that the peptide has been studied. apTKRP, *Aplysia* tachykinin–related peptide; apTKRPG-DP, apTKRP gene–derived peptide; TK, tachykinin; TKRPs, tachykinin-related peptides.

A precursor for TKRPs in *Aplysia*, named “tachykinin-2 [*A. californica*]” with accession number ADX20595 (corresponding mRNA: GU973881.1), has been deposited in the NCBI database in 2016 (Fig. 1*A*). We used this information to search the NCBI database and identified another sequence: XM_035971783 (with corresponding DNA sequence LOC101859590) (Fig. 1*B*). The coding sequence regions of the two sequences are identical suggesting that they are the same protein. Using the mRNA sequence of the coding sequence region, we also searched a second database, AplysiaTools (70), and found a DNA sequence (contig_4775) that produces an identical mRNA sequence (Fig. 1*C*). We generated gene expression maps for all three sequences and found that maps for LOC101859590 and contig_4775 both suggest the precursor protein is derived from three exons. We then designed primers (Table S1) using the above mRNA sequences as templates and performed PCR on *Aplysia* complementary DNA (cDNA). We obtained an mRNA sequence that was ∼ 500 bp in length (Figs. 1*D* and S1 for complete gels), which is consistent with the predicted length of 498 bps. This sequence is identical to the sequences predicted or obtained from NCBI (*i.e.*, GU973881.1 and XM_035971783) and AplysiaTools.

**Figure 2:**
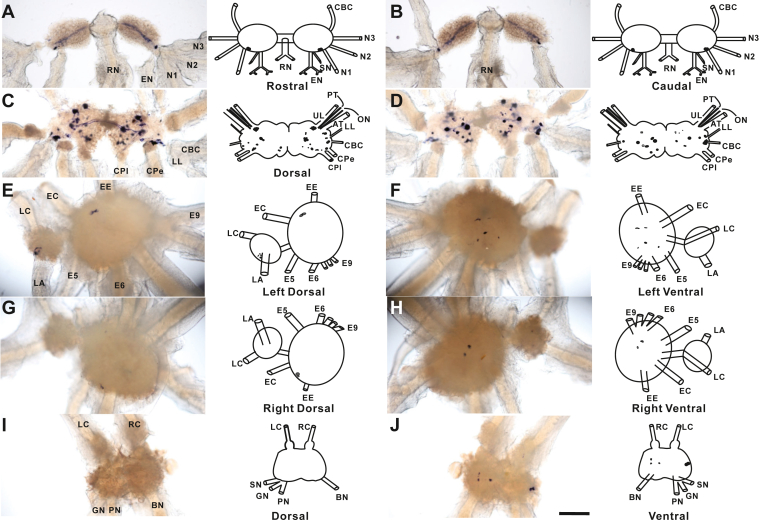
**Localization of apTKRP precursor expression in the *Aplysia* central nervous system using *in situ* hybridization.***A* and *B*, rostral (*A*) and caudal (*B*) buccal ganglia. *C* and *D*, dorsal (*C*) and ventral (*D*) cerebral ganglia. *E–H*, left dorsal (*E*), left ventral (*F*), right dorsal (*G*), and right ventral (*H*) pedal-pleural ganglia. *I* and *J*, dorsal (*I*) and ventral (*J*) abdominal ganglia. *In situ* hybridization reveals that the majority of *apTKRP*-positive neurons are distributed in the cerebral ganglia. The scale bar represents 500 μm. Buccal abbreviations are as follows: AT, anterior tentacular nerve; CBC, cerebrobuccal connective; CPe, cerebropedal connective; CPl, cerebropleural connective; EC, cerebropedal connective; EN, esophageal nerve; N1, nerve 1; N2, nerve 2; N3, nerve 3; RN, radula nerve; SN, salivary nerve. Cerebral abbreviations are as follows: LL, lower labial nerve; ON, optic nerve; PT, posterior tentacular nerve; UL, upper labial nerve. Pedal-pleural abbreviations are as follows: EE, pedal commissure; LC, cerebropleural connective; LA, pleuroabdominal connective; E5, posterior tegumentary nerve (P5); E6, anterior parapodial nerve (P6); E9, posterior pedal nerve (P9). Abdominal abbreviations are as follows: BN, branchial nerve; GN, genital nerve; LC, left pleuroabdominal connective; PN, pericardial nerve; RC, right pleuroabdominal connective; SN, siphon nerve. apTKRP, *Aplysia* tachykinin-related peptide.

To map the distribution of the apTKRP precursor mRNA and apTKRP generated peptides in the *Aplysia* central nervous system, we performed *in situ* hybridization (ISH) (Fig. 2) and immunohistochemistry experiments (Fig. S2). The specificity of the primary antibody for immunostaining was validated in preabsorption experiments (Fig. S3). Overall patterns of staining of both ISH and immunostaining were similar. In particular, we found that most apTKRP-positive neurons were in the cerebral ganglion. This is of interest since this ganglion contains a number of high-order neurons that elicit or modulate feeding and locomotion (50, 51, 52, 53, 71, 72, 73, 74, 75, 76). Thus, our data suggest that apTKRPs may play roles in regulating multiple behaviors. Additionally, we observed a few apTKRP-positive neurons in each of the four other major ganglia.

We used SignalP, 5.0, to analyze the apTKRP precursor protein and found that it contains a 22-residue signal peptide with a cleavage site between Ser^22^ and Ala^23^ (Fig. 1*E*). We then used NeuroPred (77) to predict peptides encoded by the precursor (Fig. 1*E*). They include three peptides with the conserved FXGXRamide C-terminal motif (Fig. 1, *E* and *F* and Table S2): apTKRP-1: FKPSGFMGSRamide and two peptides generated from the same “QPHLGFHGSRG” sequence in the precursor: that is, apTKRP-2a: QPHLGFHGSRamide and apTKRP-2b (with the N-terminal pyroglutamic acid modification): [p-]QPHLGFHGSRamide. Additionally, the precursor contains a fourth peptide (a linker) that does not contain the FXGXRamide motif: apTKRP gene-derived peptide (apTKRPG-DP): FMPGLESLLLAKYYQDAKW. All predicted peptides, including apTKRP-1, apTKRP-2a, apTKRP-2b, and apTKRPG-DP, were detected in extracts of *Aplysia* ganglia by MS and their sequences confirmed (Fig. 1*E*, Table S3, and Fig. S4). In addition, another linker peptide, NELAALRDHQLAASDQQGSGME, was also detected (Table S3). Notably, an analysis of TKRP precursors from several related mollusks showed that linker peptides similar to apTKRPG-DP are not unique to *Aplysia* (Fig. S5). These data elucidate three TKRPs (with the conserved FXGXR-amide motif) in *Aplysia*.

### Identifying two receptor isoforms for TKRPs *in Aplysia*

**Figure 3:**
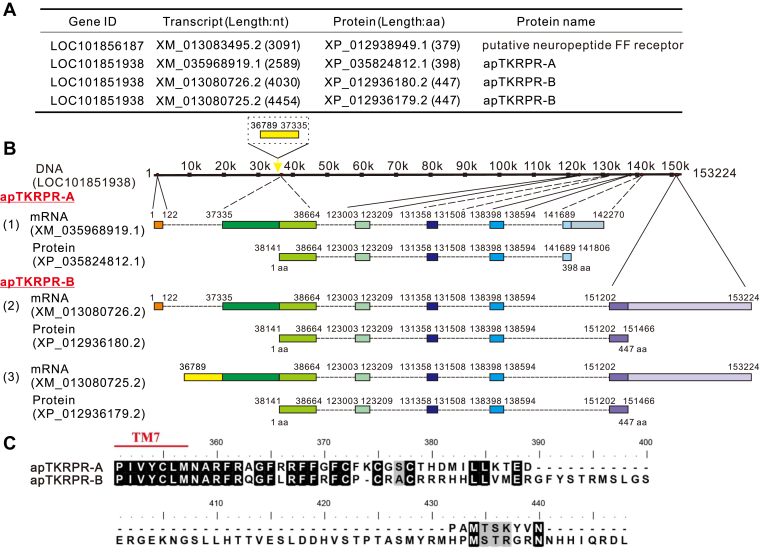
**The putative *Aplysia* tachykinin–related peptide receptors.***A*, the putative apTKRP receptors are listed in the table. A gene from NCBI (LOC101856187) corresponds to an mRNA (XM_013083495.2), which produces a protein (XP_012938949.1). In the NCBI database, this protein is named as substance-P receptor. However, in this article, we named it as putative neuropeptide FF receptor because it has the highest homology (the lowest E value) with zebrafish's neuropeptide FF receptor 2a. The second gene from NCBI (LOC101851938) produces three transcripts (XM_035968919.1, XM_013080726.2, and XM_013080725.2), corresponding to three proteins (XP_035824812.1, XP_012936180.2, and XP_012936179.2). XP_012936180.2 and XP_012936179.2 have the same protein sequence. *B* and *C*, gene expression mapping and protein sequence alignment of apTKRPRs. apTKRPR-A (1) and apTKRPR-B (2 and 3) may be derived from alternative splicing of a common primary transcript of a gene (LOC101851938), and apTKRPR-B could be generated in two different ways. The *numbers above the line* (or frame) represent the base positions, the *numbers below* represent the number of amino acids, and the *boxes* represent the exons (*B*). The difference between apTKRPR-A and apTKRPR-B lies in the C terminus (including the length and amino acid composition), and the rest are identical. *Panel C* shows the C-terminal sequences of apTKRPRs, plus the part of TM7 sequence (transmembrane domain 7) (*C*). apTKRPR, *Aplysia* tachykinin–related peptide receptor; TM, transmembrane.

**Figure 4:**
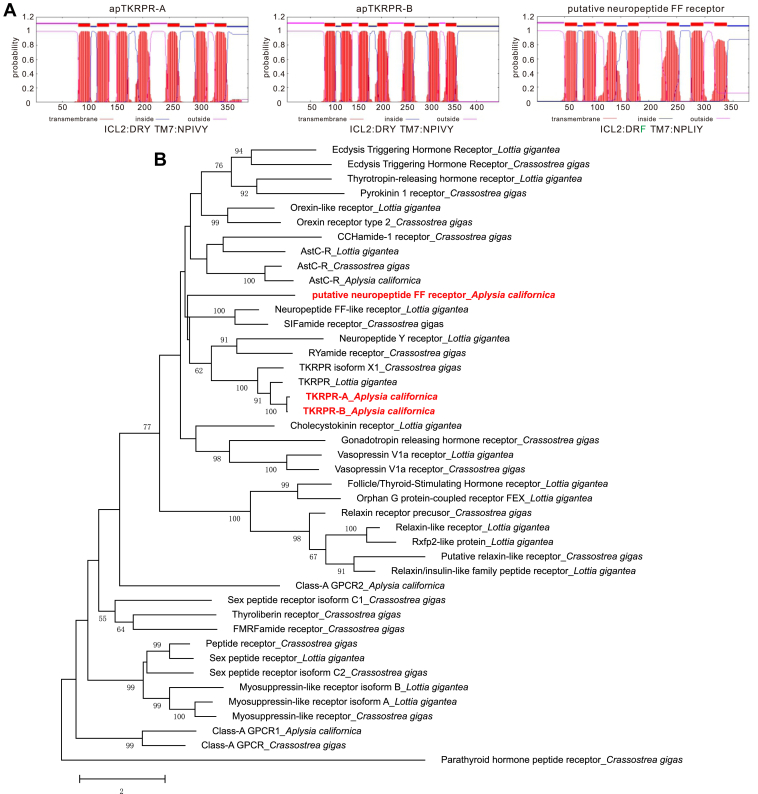
**Bioinformatics of putative apTKRP receptors.***A*, prediction of 7 TM domains of the putative receptors (*from left to right*): apTKRPR-A, apTKRPR-B, and putative neuropeptide FF receptor using TMHMM. Conserved motifs in the second intracellular loop (ICL2) and seventh TM (TM7) are shown *below*, with F residue in the DRY motif shown in *green*. *B*, a phylogenetic tree of three *Aplysia* putative receptors (shown in *red*) with a number of molluscan (*i.e.*, *Crassostrea gigas* and *Lottia gigantea*) class-A GPCR sequences from Jiang *et al.* 2022 (66) using MEGA X. Parathyroid hormone peptide receptor_*Crassostrea gigas* (a class-B GPCR) was used as an outgroup. The tree suggests that TKRPR-A and TKRPR-B could potentially be *Aplysia* TKRP receptors as they are clustered together with other molluscan TKRP receptors. The tree is drawn to scale, with branch lengths measured in the number of substitutions per site. Numbers at the nodes are bootstrap values as percentages. Only bootstrap values greater than 50 are shown. apTKRPR, *Aplysia* tachykinin–related peptide receptor; GCPR, G protein-coupled receptor; TM, transmembrane.

To identify putative receptors for the apTKRPs, we performed NCBI searches. When we used the terms “*Aplysia* TK receptor” or “*Aplysia* substance-P receptor,” we obtained one hit (XP_012938949.1) (Fig. 3*A*). When we used a TKRPR, that is, present in *C. gigas* (Dubos *et al.*, 2018) (AWI66418) to perform a BLAST search in NCBI, we had one hit (XP_012938949.1) and three hits (XP_035824812.1 and XP_012936180.2/XP_012936179.2), and the three hits were somewhat similar. Two of latter three (XP_012936179.2 and XP_012936180.2) had identical protein sequences but their mRNA differed. The latter three proteins (XP_035824812.1 vs. XP_012936180.2/XP_012936179.2) might be isoforms produced by alternative splicing of the same gene (LOC101851938) (Figs. 3, *A–C* and S6). In total, we obtained four protein sequences, with two of them identical (Fig. 3*A*), which were encoded by three mRNAs (also with three corresponding mRNAs in AplysiaTools, Table S4). To determine whether these three sequences are likely to be complete GPCRs, we used the TMHMM sever 2.0 (78, 79) and performed an NCBI Conserved Domain Search (80). All three protein sequences are predicted to have seven transmembrane (TM) domains and conserved motifs (DRY or DRF in the second intracellular loop and NPXXY in the seventh TM) (Fig. 4*A*). Thus, all three sequences are presumably class A GPCRs.

To determine whether the predicted receptors are TKRP receptors, we performed a BLAST search with each sequence in NCBI in four representative species where more sequence data are available, that is, in *Caenorhabditis elegans*, *Drosophila melanogaster*, *Danio rerio,* and *Mus musculus*. For the GPCR with accession number: XP_012938949.1, which is named substance-P receptor [*A. californica*] in NCBI, a *D. rerio* sequence named neuropeptide FF receptor 2a had the lowest E-value (3E-43) (Table S5). This suggests that this GPCR might not be a TKRP receptor. We therefore tentatively refer to it as a neuropeptide FF receptor [*A. californica*] (apNPFFR). For the two GPCRs with accession numbers XP_035824812.1 and XP_012936179.2/XP_012936180.2, which are referred to as TK-like peptide receptors in NCBI, a *Drosophila* sequence named TK-like receptor at 99D, isoform A had the lowest E-value (Table S5). This suggests that these two GPCRs are most apt to be the receptors for the TKRPs. We tentatively named them apTKRPR-A and apTKRPR-B, respectively.

**Figure 5:**
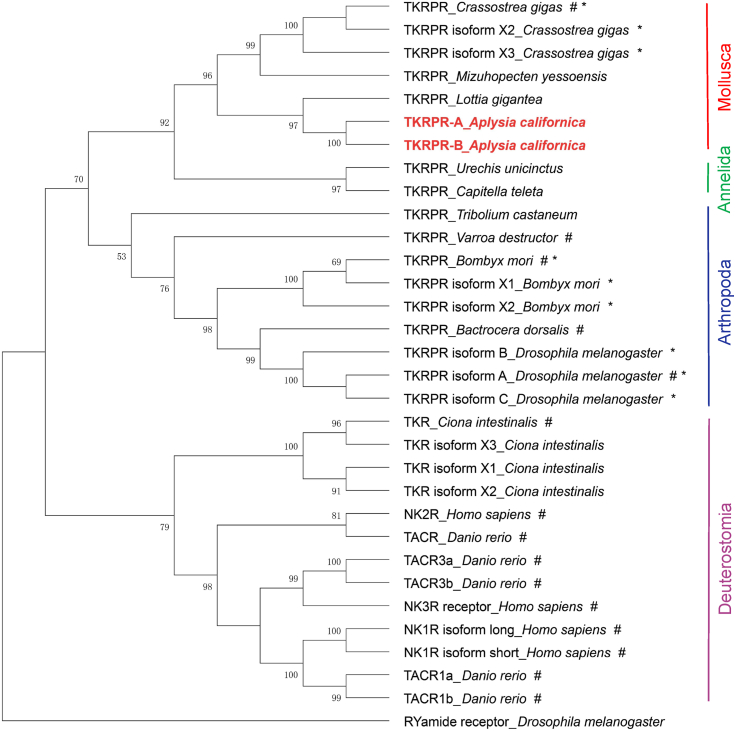
**A phylogenetic tree of apTKRPRs with TKRPRs or TKRs from selected species in protostomes (arthropods, annelids, and mollusks) and deuterostomes (see**Table S6**for information on these sequences).** The tree suggests that apTKRPRs are more closely related to *Lottia gigantea* TKRPR. The tree was generated using MEGA X with 1000 replicates.“RYamide Receptor_*Drosophila melanogaster*” (NP_524525.3) was used as an outgroup. Numbers at the nodes are bootstrap values as percentages. Only bootstrap values greater than 50 are shown. # indicates that the receptor has been studied/verified. ∗ indicates C-terminal receptor isoforms possibly generated in three species: *Crassostrea gigas*, *Bombyx mori,* and *Drosophila melanogaster* (Fig. S7). apTKRPR, *Aplysia* tachykinin–related peptide receptor; NKR, neuromedin-K receptor or neurokinin receptor; TACR, tachykinin receptor; TKR, tachykinin receptor; TKRPR, tachykinin-related peptide receptor.

To determine the phylogenetic relationship between the three GPCRs and others that have been characterized, we initially took advantage of a phylogenetic tree that had been generated showing a number of class A GPCRs in *Lottia gigantea* and *C. gigas* (66) (Fig. 5*B*, and Table S5 in (66)). We added the above three GPCR protein sequences and reran the phylogenetic tree with MEGA X (Fig. 4*B*). We found that apTKRPR-A and apTKRPR-B clustered with *Lottia* and *Crassostrea* TKRPR receptors, supporting the idea that they are indeed receptors for TKRP receptors. In contrast, the putative apNPFFR did not cluster with other TKRPRs suggesting that, as hypothesized, it is not a TKRP receptor.

To get a more complete picture of relationships between GPCRs which we characterized and receptors for TKs and TKRPs in other species, we generated a phylogenetic tree that included the apTKRPRs and TKRPRs and TKRs from selected species in protostomes (arthropods, annelids, and mollusks) and deuterostomes (Fig. 5, see Table S6 for information on these sequences). This phylogenetic tree shows that apTKRPR-A and apTKRPR-B are more closely related to *L. gigantea* TKRPR, with similarities of 62.6% and 71.0%, respectively.

Our bioinformatic analysis indicates that apTKRPR-A and apTKRPR-B are likely isoforms derived through alternative splicing of a single gene (Fig. 3, *A–C*). Isoforms for TKRs have been described in several vertebrate species (81, 82, 83), but have not previously been reported in a protostome. It is likely that isoforms for protostome TKRPRs are not unique to *Aplysia*. During our database search for the additional TKRPRs included in the phylogenetic tree shown in Figure 5, we found potential receptor isoforms in two arthropods (*i.e.*, *D. melanogaster* and *B. mori*) and in another mollusk (*C. gigas)*. To confirm that these sequences are in fact isoforms, we predicted their seven TM regions and compared them in Bioedit (Fig. S7). These data suggest that the sequences identified in each species are likely C-terminal receptor isoforms derived from alternative splicing.

**Figure 6:**
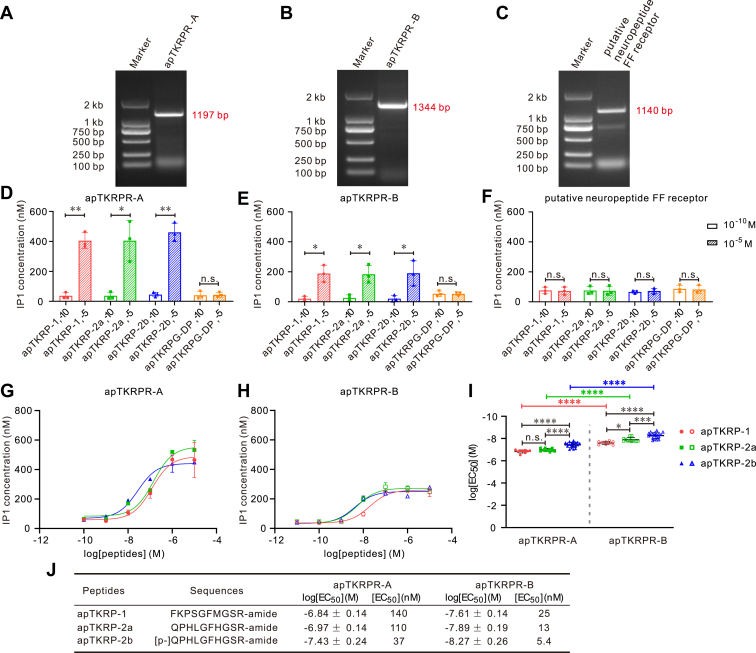
**Identification of apTKRP receptors.***A–C*, cloning of putative apTKRP receptors. The PCR products of three putative TKRP receptors (*from left to right*): *apTKRPR-A* with a length of 1197 bp, *apTKRPR-B* with a length of 1344 bp, *putative neuropeptide FF receptor* with a length of 1140 bp. Lane 1: DNA marker; Lane 2: the target gene. *D–F*, screening for potential activation of peptide ligands on putative receptors using two concentrations: 10^−10^ M and 10^−5^ M (n = 3). At 10^−10^ M, a peptide minimally activates a receptor, serving as a control. apTKRPs significantly increased IP1 concentration when acting on apTKRPR-A and apTKRPR-B, suggesting that apTKRPs are ligands for apTKRPR-A and apTKRPR-B. In contrast, apTKRPG-DP did not activate either apTKRPR-A or apTKRPR-B. Moreover, the putative neuropeptide FF receptor did not respond significantly to any of the peptides. Paired two-tailed *t* test: n.s. *p* > 0.05; ∗*p* < 0.05; and ∗∗*p* < 0.01. Error bars, SD. *G–J*, activation of apTKRPRs by three apTKRPs. *G* and *H*, representative examples of dose–response curves showing the ability of apTKRP-1, apTKRP-2a, and apTKRP-2b to activate apTKRPR-A (*G*) or apTKRPR-B (*H*) transfected in CHO-K1 cells, as determined by IP1 accumulation assay. Each data point is from two wells. *I*, comparison of log [EC_50_] is shown in (*J*). *J*, sequences of three apTKRPs and summary of the average log [EC_50_] and EC_50_ on apTKRPRs, log [EC_50_] values are reported as the mean ± SD from at least three independent experiments (apTKRP-1, n = 9; apTKRP-2a, n = 11; apTKRP-2b, n = 19). One-way ANOVA, F (5, 72) = 88.24, *p* < 0.0001. Bonferroni post hoc test: n.s. *p* > 0.05; ∗*p* < 0.05; ∗∗∗*p* < 0.001; and ∗∗∗∗*p* < 0.0001. Error bars, SD. apTKRPR, *Aplysia* tachykinin–related peptide receptor; apTKRP, *Aplysia* tachykinin–related peptide; apTKRPG-DP, apTKRP gene–derived peptide; CHO-K1, Chinese hamster ovary-K1; IP1, inositol monophosphate.

To characterize the bioactivity of the three *Aplysia* GPCRs, we used our sequence information to design primers for them (Table S1). We then performed PCR on *Aplysia* cDNA and successfully cloned mRNAs for all three receptors (Figs. 6, *A–C* and S1 for the complete gels). These data together with two independent transcriptome data from NCBI and Aplysiatools (Table S4) all provide strong support for the expression of both of the two receptor isoform mRNAs, that is, *apTKRPR-A* and *apTKRPR-B*, in *Aplysia*. Next, we inserted all three receptors into the pcDNA3.1(+) vector and then transiently transfected them in Chinese hamster ovary-K1 (CHO-K1) cells. Finally, we used the inositol monophosphate (IP1) accumulation assay (see Experimental procedures) to determine whether peptides could activate putative receptors.

We first measured IP1 responses to all four peptides, that is, the three apTKRPs (apTKRP-1, apTKRP-2a, and apTKRP-2b) and apTKRPG-DP. Peptides were applied at two concentrations (10^-10^ M and 10^-5^ M) to all three GPCRs (Fig. 6, *D–F*). At 10^-10^ M, peptide activation was minimal or undetectable, serving as a control. Both apTKRPR-A and apTKRPR-B responded to all three apTKRPs (Fig. 6, *D* and *E*) but not to apTKRPG-DP. In contrast, the putative apNPFFR did not respond to either the apTKRPs or apTKRPG-DP (Fig. 6*F*). Given the negative results for apNPFFR, we conducted one further experiment. We cotransfected it with a promiscuous Gαq-family protein also referred to as Gα16, but see (84), which binds to most GPCRs. This setup ensures that a receptor will respond if there is a ligand interaction regardless of the downstream signaling pathways it normally activates. We found that Gαq cotransfection had no effect on apNPFFR responsiveness (Fig. S8*C*). Similarly, the responses of apTKRPR-A (Fig. S8*A*) and apTKRPR-B (Fig. S8*B*) with Gαq cotransfection were identical to those shown in Figure 6, *D* and *E*. Taken together, we conclude that apTKRPR-A and apTKRPR-B are apTKRP receptors capable of associating with native Gαq proteins in CHO-K1 cells. In contrast, the putative apNPFFR is not a TKRPR.

To further characterize peptide dose–response curves, we tested multiple concentrations of the three peptides that had a significant effect on IP1 in initial screening experiments, that is, we tested apTKRP-1, apTKRP-2a, and apTKRP-2b. All three peptides were tested on both receptors (*i.e.*, apTKRPR-A and apTKRPR-B) (Fig. 6, *D* and *E*). In all cases, responses were dose-dependent. In comparing the responses of the two receptors, we found that apTKRPR-B generally had a lower EC_50_ for a given ligand (Fig. 6, *G–J*).

### The roles of specific residues and PTMs of the apTKRPs to receptor activation

**Figure 7:**
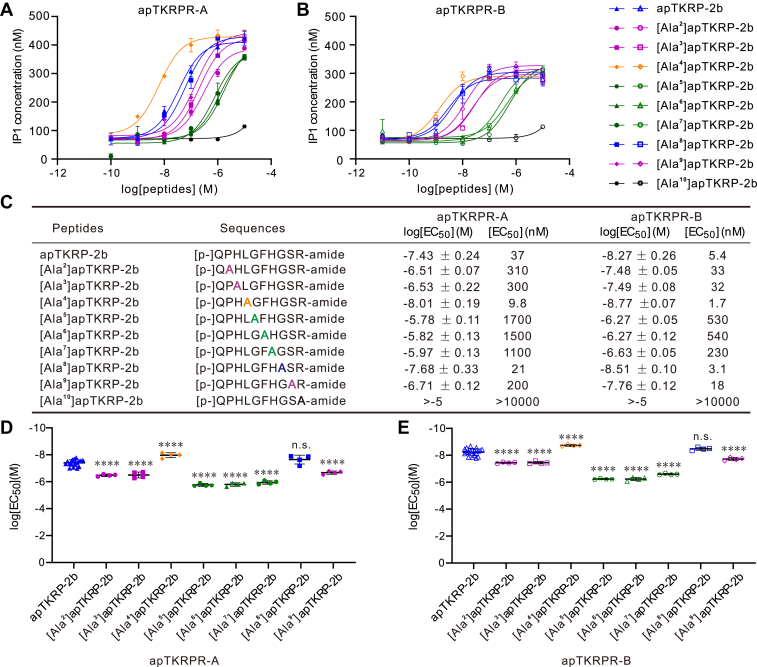
**Activation of apTKRPRs by apTKRP-2b and its analogs with Ala substitution.***A* and *B*, representative examples of dose–response curves showing the ability of apTKRP-2b analogs to activate apTKRPR-A (*A*) or apTKRPR-B (*B*). apTKRP-2b was used as a control. Each data point is from two wells. *C*, sequences of apTKRP-2b analogs and summary of the average log [EC_50_] and EC_50_ on apTKRPRs, log [EC_50_] values are reported as the mean ± SD from at least three independent experiments (apTKRP-2b, n = 19; apTKRP-2b analogs, n = 4). *D* and *E*, comparison of log [EC_50_] shown in (*C*). One-way ANOVA, F (8, 42) = 79.36, *p* < 0.0001 (*D*); F (8, 42) = 127.1, *p* < 0.0001. Bonferroni post hoc test (all compared with apTKRP-2b): n.s. *p* > 0.05; ∗∗∗∗*p* < 0.0001. Error bars, SD. apTKRPR, *Aplysia* tachykinin–related peptide receptor; apTKRP, *Aplysia* tachykinin–related peptide.

To determine specific residues that might be critical for receptor activation, we conducted further experiments with apTKRP-2b, the peptide with the lowest EC_50_. We generated nine analogs of it, each had a different alanine substitution, for example, one peptide was [Ala^2^]apTKRP-2b, a second peptide was [Ala^3^]apTKRP-2b, *etc.* (Fig. 7). All peptides were tested on both receptors. We found that analogs could be divided into five groups based on their EC_50_ values. [Ala^10^]apTKRP-2b was in the first group. It was unable to activate either apTKRPR-A or apTKRPR-B. This indicates that a C-terminal Arg is necessary for the activity of apTKRP-2b. The second group consisted of [Ala^5^]apTKRP-2b, [Ala^6^]apTKRP-2b, and [Ala^7^]apTKRP-2b. These peptides had EC_50_ values that were similar to each other and were significantly higher than the EC_50_ of the original peptide. The third group consisted of [Ala^2^]apTKRP-2b, [Ala^3^]apTKRP-2b, and [Ala^9^]apTKRP-2b. These peptides had EC_50_ values that were increased but not as much as the peptides in the second group. [Ala^8^]apTKRP-2b was in the fourth group. Its potency was only slightly reduced and the effect was not statistically significant. [Ala^4^]apTKRP-2b was in the fifth group. Its potency was higher than that of the original peptide. Except for [Ala^10^]apTKRP-2b, all of the analogs tested could reach similar maximum concentrations of IP1 (E_max_), that is, they displayed a similar efficacy.

That there was no significant difference between the EC_50_ of [Ala^8^]apTKRP-2b and the original peptide was somewhat surprising since the Gly at position 8 is highly conserved in both protostomes and deuterostomes (Fig. 1*F*). Gly and Ala are similar in that they are both small and hydrophobic. To determine whether these properties are important for bioactivity, we conducted experiments in which we generated analogs with three additional amino acid substitutions (85). Instead of Gly, one analog had Leu at position eight (which is hydrophobic but has a larger molecular weight). A second analog had Asp (which is small but is negatively charged), and a third had Lys (which is also small but is positively charged). We found that all of these analogs had dramatically increased EC_50_ values for both receptor isoforms, that is, EC_50_ values exceeded 10^−5^ M (Fig. S9). These data indicate that small and hydrophobic amino acid at position 8 is necessary for receptor activation.

**Figure 8:**
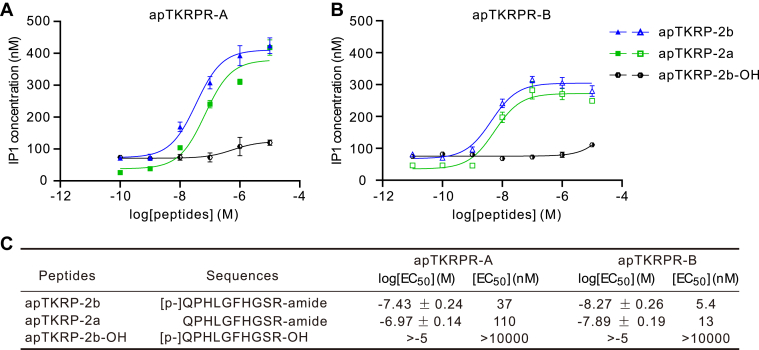
**Activation of apTKRPRs by apTKRP-2b, apTKRP-2a (without the N-terminal pyroglutamate cyclization), and apTKRP-2b-OH (without the C-terminal amidation).***A* and *B*, representative examples of dose–response curves showing the ability of apTKRP-2b, apTKRP-2a, and apTKRP-2b-OH to activate apTKRPR-A (*A*) or apTKRPR-B (*B*). apTKRP-2b was used as a control. Each data point is from two wells. *C*, sequences of apTKRP-2b, apTKRP-2a, and apTKRP-2b-OH and summary of the average log [EC_50_] and EC_50_ on apTKRPRs, log [EC_50_] values are reported as the mean ± SD from at least three independent experiments (apTKRP-2b, n = 19; apTKRP-2a, n = 11; apTKRP-2b-OH, n = 4). apTKRP, *Aplysia* tachykinin–related peptide; apTKRPR, *Aplysia* tachykinin–related peptide receptor.

To determine the potential influence of PTMs, that is, the C-terminal amidation and N-terminal pyroglutamate cyclization, we compared the bioactivity of three peptides. The first, the native apTKRP-2b, had both PTMs. The second, the apTKRP-2b analog apTKRP-2b-OH, had the N-terminal pyroglutamate cyclization but was not amidated. The third peptide, apTKRP-2a, was amidated but did not have the N-terminal pyroglutamate cyclization (Fig. 8). apTKRP-2b-OH was virtually inactive indicating that the C-terminal amidation is necessary for bioactivity. apTKRP-2a was less potent than apTKRP-2b (for statistical comparison between log[EC_50_] of apTKRP-2b and apTKRP-2a, Fig. 6*I*), which indicates that the N-terminal pyroglutamate cyclization increases bioactivity.

Overall, the above results indicate that the highly conserved C-terminal arginine residue and amidation are necessary for the activity of apTKRP-2b. Other modifications impact bioactivity but are less critical.

### Agonist-induced desensitization of two apTKRPR isoforms: phosphorylation-dependent and phosphorylation-independent mechanisms

**Figure 9:**
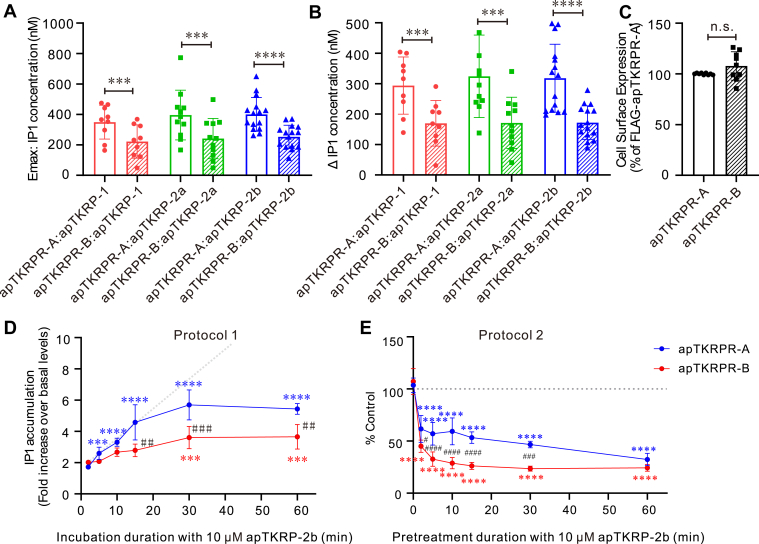
**Identification of apTKRPR desensitization.***A* and *B*, comparison of IP1 concentration of apTKRPR-A and apTKRPR-B, including the maximum concentration (E_max_) (*A*) and concentration difference (ΔIP1) (*B*) (apTKRP-1, n = 9; apTKRP-2a, n = 11; apTKRP-2b, n = 15). Paired two-tailed *t* test: ∗∗∗*p* < 0.001; ∗∗∗∗*p* < 0.0001. The statistical data are derived from the experimental results shown in Figure 6. For comparison of IP1 concentrations, we used only data from the paired experiments. *C*, cell surface expression of apTKRPRs. Data are presented as a percentage of the maximal expression measured for the FLAG-apTKRPR-A (set at 100%) (n = 9). Paired two-tailed *t* test: n.s. *p* = 0.13. *D* and *E*, desensitization of IP1 production in CHO-K1 cells. *D*, time-course for apTKRP-2b-induced IP1 accumulation in CHO-K1 cells transiently expressing apTKRPRs. Data are presented as a fold increase over basal IP1 concentration (n = 3). We performed a two-way ANOVA. The two main factors in this analysis were the receptor types and incubation duration. There was a significant effect of receptor types (F (1, 4) = 8.691, *p* = 0.0420), incubation duration (F (5, 20) = 54.29, *p* < 0.0001), and interaction of these two main factors (F (5, 20) = 9.199, *p* = 0.0001). Bonferroni post hoc test: comparison of incubation duration (the control group is the 2 min incubation duration), ∗∗∗*p* < 0.001, ∗∗∗∗*p* < 0.0001; comparison of receptor types, ## *p* < 0.01, ### *p* < 0.001. *E*, desensitization of apTKRPRs as measured by IP1 accumulation in response to different pretreatment duration (2 min, 10 min, 15 min, 30 min, or 60 min) with apTKRP-2b. Data are presented as a percentage of control apTKRP-2b stimulation in the absence of pretreatment (pretreatment duration = 0 min, n = 5). We performed a two-way ANOVA. The two main factors in this analysis were the receptor types and pretreatment duration. There was a significant effect of receptor types (F (1, 8) = 70.78, *p* < 0.0001), pretreatment duration (F (6, 48) = 110.9, *p* < 0.0001), and interaction of these two factors (F (6, 48) = 6.170, *p* < 0.0001). Bonferroni post hoc test: comparison of pretreatment duration, ∗∗∗∗ *p* < 0.0001; comparison of receptor types, # *p* < 0.05, ### *p* < 0.001, and #### *p* < 0.0001. *Gray dotted lines* represent receptors not showing desensitization. Error bars, SD. apTKRP, *Aplysia* tachykinin–related peptide; apTKRPR, *Aplysia* tachykinin–related peptide receptor; CHO-K1, Chinese hamster ovary-K1; IP1, inositol monophosphate.

In comparing the two TKRP receptors, we noted that the E_max_ produced by the activation of apTKRPR-B appears to be lower for apTKRPR-A (Fig. 6, *G* and *H*). However, EC_50_ values for apTKRPR-B also appear to be lower (Fig. 6, *I* and *J*). To further study these differences, we compared the accumulation of intracellular IP1 after the activation of apTKRPRs. The results showed that both the E_max_ and Δ IP1 (the difference between the lowest and highest IP1 concentration) of apTKRPR-B were significantly lower than those of apTKRPR-A (Fig. 9, *A* and *B*). To determine whether the difference in efficacy was due to a difference in the level of expression of the two receptors, we constructed apTKRPRs with a FLAG tag on the N terminus, transiently transfected CHO-K1 cells, and measured receptor expression levels using ELISA. The mean expression level of apTKRPR-B on the cell membrane was slightly higher, but the difference was not statistically significant (Fig. 9*C*).

Interestingly, the two receptors differ structurally in the intracellular region near the C terminus (Fig. 3*C*). Namely, the C terminus of apTKRPR-B contains more Ser/Thr residues. This is of interest since phosphorylation of C-terminal Ser/Thr residues is one of the main mechanisms of GPCR desensitization (25, 86). Therefore, we speculated that apTKRPR-B might undergo a higher degree of desensitization due to its higher number of Ser/Thr residues. If this were true, it might explain why the total IP1 accumulation of apTKRPR-B was less despite the lower EC_50_ value.

To further probe this idea, we used two protocols (see “Experimental procedures” section). The first (protocol 1) was used to test effects of long term agonist exposure (Fig. S10) (87, 88). Specifically, we exposed the two receptor isoforms to 10 μM apTKRP-2b for different periods of time and measured the IP1 accumulation rate (Fig. 9*D*). For both receptors, we observed that IP1 levels initially increased and then reached a plateau indicating that both receptors underwent agonist-induced desensitization. At 15, 30, and 60 min, IP1 accumulation was greater for cells transfected with apTKRPR-A, indicating that apTKRPR-B had a higher degree of desensitization. The second protocol (protocol 2) (89) was used to test the effects of repeated agonist exposure (Fig. S11). Specifically, we exposed the two receptor isoforms to 10 μM apTKRP-2b for different pretreatment durations and then examined their response to a subsequent challenge (15 min exposure with the agonist) (Fig. 9*E*). In both cases, IP1 accumulation was less after the second exposure indicating that desensitization had occurred. Again, IP1 accumulation was less for cells expressing the apTKRPR-B receptor indicating more significant or rapid desensitization. Thus, the results of both sets of experiments support the idea that apTKRPR-A and apTKRPR-B both undergo desensitization, and that desensitization is greater for apTKRPR-B.

**Figure 10:**
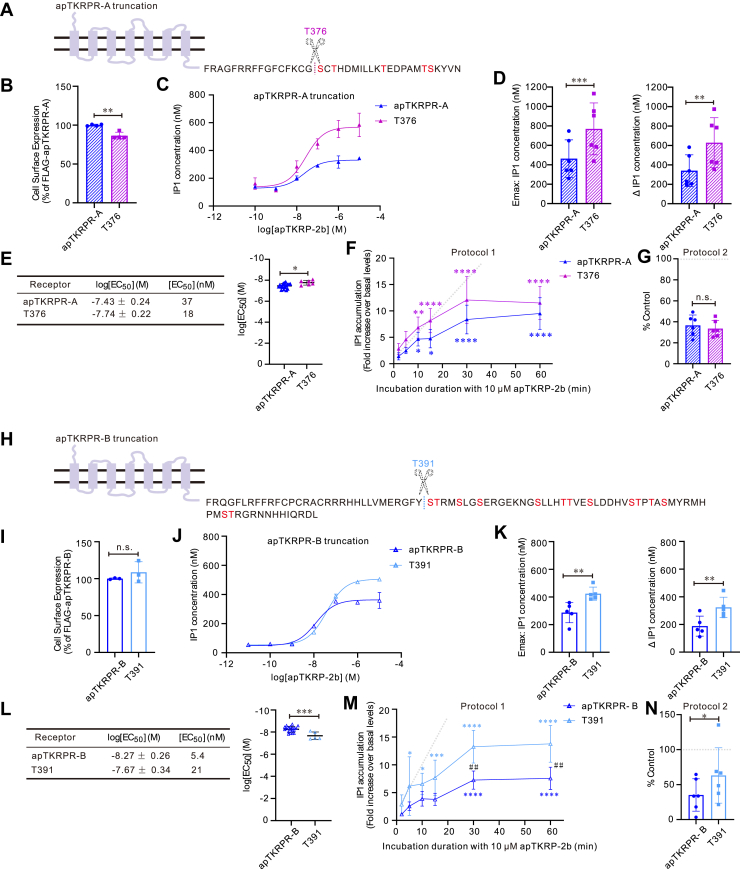
**The effect of C-terminal Ser/Thr residue truncation on apTKRPR-A and apTKRPR-B.***A* and *H*, schematic diagrams illustrating T376 or T391 truncates with all ser/thr residues removed. *B* and *I*, cell surface expression of apTKRPRs and truncates. Data are presented as a percentage of the maximal expression measured for the FLAG-apTKRPRs (set at 100%) (apTKRPR-A and apTKRPR-A T376; n = 4; apTKRPR-B and apTKRPR-B T391, n = 3). Paired two-tailed *t* test: ∗∗*p* = 0.006 (*B*); n.s. *p* = 0.401 (*I*). *C* and *J*, representative examples of dose–response curves showing the ability of apTKRP-2b to activate apTKRPRs and truncates. Each data point is from two wells. *D* and *K*, comparison of IP1 concentration of apTKRPRs and truncates, including the maximum concentration (E_max_) and concentration difference (ΔIP1) (apTKRPR-A and apTKRPR-A T376, n = 6; apTKRPR-B and apTKRPR-B T391, n = 5). Paired two-tailed *t* test: ∗∗*p* < 0.01 and ∗∗∗*p* < 0.001. For comparison of IP1 concentration, we used only data from the paired experiments. *E* and *L*, summary of the average log [EC_50_] and EC_50_ on apTKRPRs and truncates, log [EC_50_] values are reported as the mean ± SD from at least three independent experiments (apTKRPR-A, n = 19; apTKRPR-A T376, n = 6; apTKRPR-B, n = 19; apTKRPR-B T391, n = 5). Unpaired two-tailed *t* test: ∗*p* = 0.01 (*E*) and ∗∗∗*p* = 0.0003 (*L*). *F* and *M*, time-course for apTKRP-2b–induced IP1 accumulation in CHO-K1 cells transiently expressing apTKRPRs and truncates. Data are presented as a fold increase over basal IP1 concentration (n = 5). We performed a two-way ANOVA. The two main factors in this analysis were the receptor types and incubation duration. *F*, there was a significant effect of incubation duration (F (5, 40) = 53.49, *p* < 0.0001), but no significant effect of receptor types (F (1, 8) = 4.337, *p* = 0.0708). *M*, there was a significant effect of receptor types (F (1, 8) = 10.13, *p* = 0.0129), incubation duration (F (5, 40) = 44.37, *p* < 0.0001), and interaction of these two main factors (F (5, 40) = 2.984, *p* = 0.0221). Bonferroni post hoc test: comparison of incubation duration (the control group is 2 min incubation duration), ∗*p* < 0.05, ∗∗*p* < 0.01, ∗∗∗*p* < 0.001, and ∗∗∗∗*p* < 0.0001; comparison of receptor types ## *p* < 0.01. *G* and *N*, desensitization of apTKRPRs and truncates as measured by IP1 accumulation in response to pretreatment (60 min) with apTKRP-2b. Data are presented as a percentage of control apTKRP-2b stimulation in the absence of pretreatment (n = 6). Paired two-tailed *t* test: n.s. *p* = 0.32 (*G*); ∗ *p* = 0.038 (*N*). *Gray dotted lines* represent receptor responses as expected for those without desensitization. Error bars, SD. apTKRP, *Aplysia* tachykinin–related peptide; apTKRPR, *Aplysia* tachykinin–related peptide receptor; CHO-K1, Chinese hamster ovary-K1; IP1, inositol monophosphate.

In previous studies, researchers used truncated receptors to study the effects of C-terminal phosphorylation on desensitization (27, 29). We took a similar approach (Fig. 10, *A* and *H*) and tested the effect of removing C-terminal sequences on desensitization using the two protocols described above (protocols 1 and 2). For apTKRPR-A, the truncation had no significant effect on desensitization (Fig. 10, *F* and *G*). However, the truncation increased receptor activity (Fig. 10, *C–E*) and reduced the surface expression level (Fig. 10*B*), making results difficult to interpret. For apTKRPR-B, truncation decreased both the activation potency (Fig. 10, *J–L*) and desensitization (Fig. 10, *M* and *N*) without affecting surface expression (Fig. 10*I*). To investigate whether ligand exposure affects the expression of the truncated apTKRPR-B receptor (T391) and consequently reduces its activation potency, we conducted ELISA experiments. These experiments assessed the expression levels of the truncated receptor after 60 min of ligand exposure. The results indicated no significant change in receptor expression (Fig. S12*A*), suggesting that a change in receptor expression due to ligand exposure is not a major factor.

**Figure 11:**
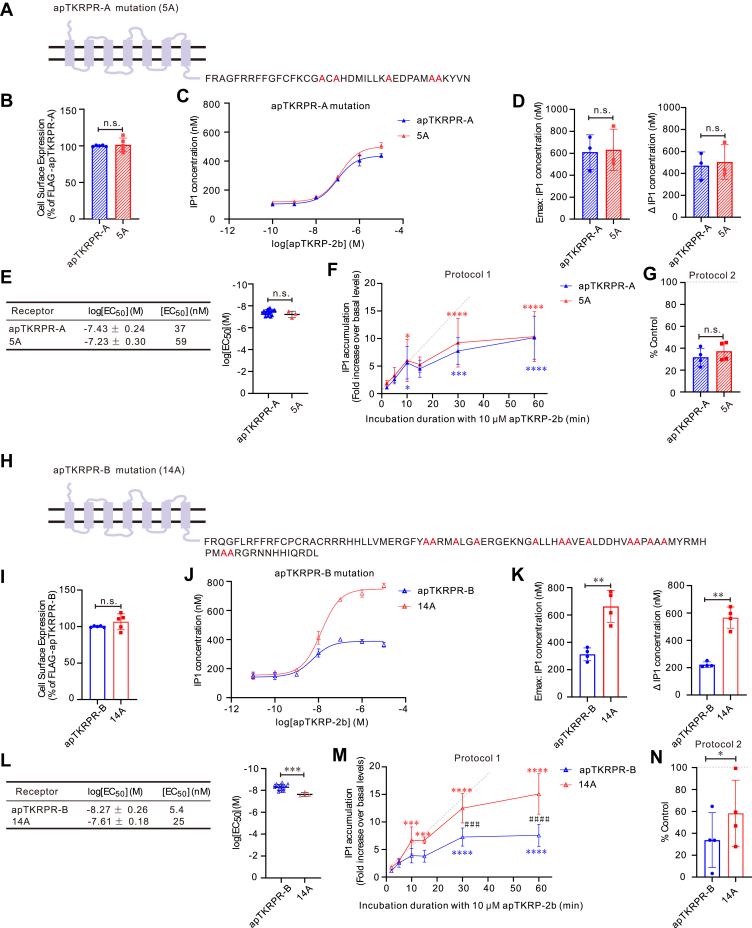
**The effect of C-terminal Ser/Thr residue mutation on apTKRPR-A and apTKRPR-B.***A* and *H*, schematic diagrams illustrating apTKRPR-A 5A or apTKRPR-B 14A mutants with all ser/thr residues mutated to ala. *B* and *I*, cell surface expression of apTKRPRs and mutants. Data are presented as a percentage of the maximal expression measured for the FLAG-apTKRPRs (set at 100%) (n = 5). Paired two-tailed *t* test: n.s. *p* = 0.73 (*B*); n.s. *p* = 0.25 (*I*). *C* and *J*, representative examples of dose–response curves showing the ability of apTKRP-2b to activate apTKRPRs and mutants. Each data point is from two wells. *D* and *K*, comparison of IP1 concentration of apTKRPRs and mutants, including the maximum concentration (E_max_) and concentration difference (ΔIP1) (apTKRPR-A and apTKRPR-A 5A, n = 3; apTKRPR-B and apTKRPR-B 14A, n = 4). Paired two-tailed *t* test: n.s. *p* > 0.05 and ∗∗*p* < 0.01. For comparison of IP1 concentration, we used only data from the paired experiments. *E* and *L*, summary of the average log [EC_50_] and EC_50_ on apTKRPRs and mutants, log [EC_50_] values are reported as the mean ± SD from at least three independent experiments (apTKRPR-A, n = 19; apTKRPR-A 5A, n = 3; apTKRPR-B, n = 19; apTKRPR-B 14A, n = 4). Unpaired two-tailed *t* test: n.s. *p* = 0.19 (*E*); ∗∗∗ *p* < 0.001 (*L*). *F* and *M*, time-course for apTKRP-2b–induced IP1 accumulation in CHO-K1 cells transiently expressing apTKRPRs and mutants. Data are presented as a fold increase over basal IP1 concentration (n = 5). We performed a two-way ANOVA. The two main factors in this analysis were the receptor types and incubation duration. *F*, there was a significant effect of incubation duration (F (5, 40) = 25.51, *p* < 0.0001), but no significant effect of receptor types (F (1, 8) = 0.3199, *p* = 0.5872). *M*, there was a significant effect of receptor types (F (1, 8) = 20.52, *p* = 0.0019), incubation duration (F (5, 40) = 67.97, *p* < 0.0001), and interaction of these two main factors (F (5, 40) = 8.441, *p* < 0.0001). Bonferroni post hoc test: comparison of incubation duration (the control group is 2 min incubation duration), ∗*p* < 0.05, ∗∗∗*p* < 0.001, and ∗∗∗∗*p* < 0.0001; comparison of receptor types, ###, *p* < 0.001, and ####, *p* < 0.0001. *G* and *N*, desensitization of apTKRPRs and mutants as measured by IP1 accumulation in response to pretreatment (60 min) with apTKRP-2b. Data are presented as a percentage of control apTKRP-2b stimulation in the absence of pretreatment (n = 4). Paired two-tailed *t* test: n.s. *p* = 0.086 (*G*); ∗*p* = 0.012 (*N*). *Gray dotted lines* represent receptor response as expected for those without desensitization. Error bars, SD. apTKRP, *Aplysia* tachykinin–related peptide; apTKRPR, *Aplysia* tachykinin–related peptide receptor; CHO-K1, Chinese hamster ovary-K1; IP1, inositol monophosphate.

To further probe the role of C-terminal Ser/Thr residues on receptor desensitization, we conducted an additional set of experiments in which we mutated all C-terminal Ser/Thr residues with the lengths of the receptors remaining the same (Fig. 11, *A* and *H*). For both receptor isoforms, mutations did not change receptor surface expression (Fig. 11, *B* and *I*). For apTKRPR-A, mutations also did not affect receptor activity (Fig. 11, *C–E*) or desensitization (Fig. 11, *F* and *G*) indicating that desensitization is not dependent on C-terminal phosphorylation. In contrast, for apTKRPR-B, mutations decreased the activation potency (Fig. 11, *J–L*) and desensitization (Fig. 11, *M* and *N*). These latter results are similar to those obtained in the truncation experiments (Fig. 10, *M* and *N*). Thus, the desensitization of apTKRPR-B may be at least partially dependent on C-terminal phosphorylation. However, the truncates and mutants of apTKRPR-B displayed a slower rate of IP1 accumulation as incubation duration increased in protocol 1 (Figs. 10*M*, and 11*M*) and ∼60% IP1 accumulation of the control in protocol 2 (Figs. 10*N*, and11*N*). This suggests that even the truncated or mutated apTKRPR-B still had a residue level of desensitization, which is C-terminal phosphorylation-independent. Thus, the higher degree of desensitization of apTKRPR-B is likely not attributable to its more Ser/Thr residues, but, instead, attributable to its phosphorylation-dependent and phosphorylation-independent mechanisms, whereas desensitization of apTKRPR-A only has the phosphorylation-independent component.

Finally, to examine the potential contribution of receptor internalization to desensitization, we conducted ELISA experiments to assess changes in the expression of apTKRPR-A and apTKRPR-B receptors following exposure to apTKRP-b (10 μM) for various durations. The results, illustrated in Fig. S11, *B* and *C*, suggest that receptor internalization likely occurs during the initial stages of ligand*–*receptor interaction, particularly after approximately 10 min of exposure for both receptor types. However, the rate of internalization appears to diminish with prolonged exposure.

## Discussion

In this work, we characterized the apTKRP signaling system through bioinformatics, molecular biology, MS, and a cell-based assay and provided the first evidence for the presence of two TKRP receptor isoforms in a protostome. We further explored the role of individual residues and PTMs of the TK peptides in receptor activation. More interestingly, we demonstrated that both receptor isoforms appear to undergo C-terminal phosphorylation-independent desensitization and that one receptor isoform additionally undergoes desensitization that is phosphorylation-dependent.

### The apTKRP signaling system and alternative splicing of the *apTKRPR*

The TKRP signaling system has been studied in a number of protostome species, but the existing research has mainly focused on arthropods (4). In the superphylum lophotrochozoan, the TKRP signaling system has only been successfully identified in the annelid *U. unicinctus* (35, 36, 37, 38) and the molluscs *O. vulgaris* (39, 40) and *C. gigas* (41). One precursor and one receptor were characterized in each species. Previous studies that screened databases for potential peptides suggested that TKRPs were likely to be present in *Aplysia* (8). In the present work, we experimentally demonstrated this by successfully characterizing one apTKRP precursor and two apTKRPRs.

Most protostome species have one TKRP precursor gene. There are, however, exceptions to this observation, that is, two mollusks (*Deroceras reticulatum* and *L. gigantea*) and two annelids (*Platynereis dumerilii* and *Tetranychus urticae*) (90, 91, 92, 93) have two precursors. We tried to determine whether there is a second TKRP precursor in *Aplysia* using bioinformatics but were not able to identify one. Consistent with the idea that a second precursor does not exist, we found that all of the peptides that we detected in ganglion extracts using MS are encoded in the one precursor that we characterized (Figs. 1*E*, S4, and Table S3). Taken together, this suggests that *Aplysia* only has one TKRP precursor.

We localized the apTKRP precursor to specific neurons using ISH (Fig. 2). Similar pattern of staining was also obtained with immunohistochemistry (Fig. S2). We found that apTKRP-postive neurons are primarily expressed in the cerebral ganglion. This ganglion contains command-like neurons that project their axons to other ganglia, such as the buccal ganglion or the pedal ganglion. These other ganglia directly control feeding (50, 52, 53, 72, 73, 74, 75, 76) or locomotor (51, 71, 94) behaviors. We therefore suggest that the TKRPs serve a variety of modulatory functions in *Aplysia*. Mapping studies of putative TKRPs have been conducted in other molluscs (*i.e.*, *Lymnaea stagnalis* and *Helix pomatia*) using LomTK-I antisera from the insect *Locusta migratoria* (95, 96, 97). In *Lymnaea*, positive neurons were mainly distributed in the cerebral and pedal ganglion. About 1000 (809–971) positive neurons were observed in *Helix* with about 88.25% of these located in the cerebral ganglion (the procerebrum is the major site). Although these findings in *Lymnaea* and *Helix* need to be verified with ISH or more specific antibodies, the overall distributions described are consistent with our results in that the cerebral ganglion is one of the main sources of the TKRPs.

We also identified two receptors that can be effectively activated by three apTKRPs (Fig. 6). These two receptors are likely derived from alternative splicing of the same gene (Fig. 3, *A* and *B*), and the two isoforms differ only at the intracellular C terminus (Fig. 3*C*). Alternative splicing is an important mechanism for the production of multiple proteins from a single gene and isoforms produced in this manner can vary in their tissue distribution and function (11, 98). About 50% of GPCR genes are believed to undergo alternative splicing, producing variants that differ in their N terminus or C terminus or intracellular or extracellular loops (10, 12). Indeed, alternative splicing has been described in the TK signaling system in several vertebrate species, and in these species only C-terminal isoforms have been identified so far (81, 82, 83). For example, TK receptors in mammals have been divided into three major types (from different genes), that is, NK1R, NK2R, and NK3R (4, 33). Among them, NK1R and NK2R genes can produce different isoforms (NK1R: NK1R long and NK1R short; NK2R: NK2R-α and NK2R-β) through alternative splicing (23, 99), and the C termini of these isoforms largely overlap, with one longer than the other. These isoforms have different tissue distributions, different biological activity, and signal transduction properties (19, 20, 21, 22). Thus, the two isoforms of apTKRPR identified here likely increase the diversity and complexity of the apTKRP signaling system (see sections below). Notably, the C termini of the two *Aplysia* isoforms differ not only in their lengths but also in their sequences, which is most similar to the two NK1 receptor isoforms of *Bufo marinus*, that is, bNK1-B receptor and bNK1-C receptor (100). Moreover, in all protostome species investigated so far, only one gene encoding a single TKRP receptor in each species has been characterized. Our study is the first to show that a protostome TKRPR gene can produce two functional isoforms through alternative splicing. That said, our bioinformatic analysis predicts that there might be TKRP receptor isoforms in three additional protostome species (*e.g.*, sequences marked with ∗ in Figs. 5, and S7), although this prediction needs to be confirmed in future bioinformatic and experimental studies.

### Actions of single residues and PTMs of apTKRPs on receptor activity

The fact that only the apTKRPs are active whereas apTKRPG-DP is not (Fig. 6) suggests that the conserved C-terminal FXGXR-amide motif is critical for receptor activity. To formally determine the amino acid residues of apTKRPs essential for receptor activity, we performed a structure-activity relationship analysis using a series of synthetic analogs of apTKRP-2b in which each amino acid was sequentially substituted by Ala (Fig. 7). Considering the conserved C-terminal FXGXR-amide motif of protostome TKRPs, the substitution of the terminal Arg and the first Phe did have a drastic negative impact on receptor activity, consistent with the highly conserved motif of these two residues. In contrast, the substitution of the middle Gly residue did not significantly alter apTKRP-2b activity. This suggests that the middle Gly is not crucial. Consistent with this idea, it is not conserved in all species. In some mollusks (*i.e.*, *L. gigantea*, *C. gigas*, *O. vulgaris,* and *Anodonta cygnea*) (40, 41) and in an arthropod (*D. melanogaster*) (101), this residue is either Ala or Pro (see residues highlighted in blue in Table S2). It is, however, also possible that an Ala (or Pro) substitution for Gly has little effect partly because Ala and Pro are like Gly in that they are hydrophobic and relatively small. When we replaced the middle Gly with three additional amino acids that differed from Gly in either polarity or size (85) (Fig. S9), we found that all three substitutions dramatically increased EC_50_ values. This indicates that the chemical nature of the amino acid in the middle position is important. Interestingly, in addition to these highly conserved residues, the substitution of other less conserved amino acid residues in apTKRP-2b also has a significant impact on the activity of apTKRP-2b albeit with a somewhat smaller effect. In the future, it will be interesting to determine if our findings also apply to other TKs or TKRPs.

We also studied the effects of PTMs of the apTKRPs (Fig. 8). The apTKRP precursor can produce a single copy of three apTKRPs: apTKRP-1, apTKRP-2a, and apTKRP-2b (Fig. 1*E*). apTKRP-2a differs from apTKRP-2b in that the N terminus of apTKRP-2b contains pyroglutamic acid, that is, has a cyclization modification. The EC_50_ value of apTKRP-2b was lower than that of apTKRP-2a (Fig. 6, *I* and *J*), indicating that the N-terminal pyroglutamate cyclization modification enhances bioactivity. Although N-terminal pyroglutamic acid cyclization is a common form of PTM in general, TKs/TKRPs with this modification have only been described in a few protostome species (92, 101, 102, 103). In these species, there were no data indicating whether or not the cyclization impacts bioactivity. Therefore, our finding is the first to show that it increases the potency of TKs/TKRPs. Cyclization of gonadotropin-releasing hormone can also occur, and in this context, it can impact aminopeptidase-mediated degradation, which is likely to prolong the action time of gonadotropin-releasing hormone (104). It remains to be determined if this is also the case for apTKRP-2b.

We also determined the effect of the C-terminal amidation on the activity of apTKRPs by removing it (Fig. 8). Our data showed that amidation is necessary for the activity of apTKRP-2b. C-terminal amidation is a relatively common PTM, and a large number of studies have demonstrated that it often plays a critical role in determining bioactivity in other contexts (64, 67, 69, 105, 106). To our knowledge, we are the first to show that this is also the case for TKs/TKRPs.

### Agonist-induced desensitization of apTKRPRs

Agonist-induced TKR desensitization has been demonstrated in several vertebrate species (19, 21, 27, 28, 29, 30, 31, 32), especially NK1R, but the mechanism of desensitization remains controversial. In general, most studies have suggested that it is primarily a consequence of the phosphorylation of C-terminal Ser/Thr residues (27, 28, 29, 30, 31, 32). However, other studies have suggested that this may not be the case, that is, they have found that protein kinase inhibitors or partial truncation of the C terminal can have no impact on receptor desensitization (19, 21). The differences in these results could be partly due to the fact that different studies have used different cell lines or methods. Alternatively, other systems, including the vertebrate TK systems, may be like the *Aplysia* system in that there is more than one mechanism for receptor desensitization (see below). In addition, we note that desensitization has also been demonstrated in a single TKRPR of an arthropod, *B. mori*, which showed a phosphorylation-dependent mechanism (42).

To show different mechanisms of TKRPR desensitization, we took advantage of the fact that the *Aplysia* receptor has two isoforms, that is, apTKRPR-A and apTKRPR-B. These two isoforms only differ in their C-terminal intracellular segment. We studied the desensitization mechanisms of both isoforms by constructing C-terminal truncates and mutants with alanine substitution in the same experimental system. We used two protocols to apply agonists. In the first, agonist application was prolonged (protocol 1, Fig. S10) (87, 88); in the second, agonist application was repeated (protocol 2, Fig. S11) (89). Notably, results obtained using the two protocols were similar. For apTKRPR-A, the C-terminal truncation did not affect desensitization (Fig. 10, *F* and *G*). However, it had a paradoxical effect in that it reduced receptor surface expression, but at the same time increased receptor activity (Fig. 10, *A*–*F*). In other contexts, an effect of the C terminus has been demonstrated on receptor membrane localization (107), ligand binding (108), and biological activity (109). For example, after the C terminus of either glucagon-like peptide-2 receptor or chemoattractant receptor homologous molecule expressed on T helper type 2 cells is truncated, activation signals increase despite the fact that receptor expression levels decrease. This suggests that in these systems, the C terminus may have an inhibitory effect on receptor signaling (109, 110). This may also be the case for apTKRPR-A. Regardless, mutations with the C-terminal ala substitution of apTKRPR-A did not affect receptor activity, desensitization, or receptor expression (Fig. 11, *A–F*). This unequivocally demonstrates that desensitization of apTKRPR-A is not dependent on C-terminal phosphorylation.

For apTKRPR-B, both truncation (Fig. 10, *G–L*) and Ala substitution (Fig. 11, *G–L*) had similar effects, that is, both manipulations decreased potency and desensitization without affecting the receptor surface expression. Thus, the data suggest that desensitization of apTKRPR-B appears to be at least partially dependent on C-terminal phosphorylation. Importantly, truncates and mutants of apTKRPR-B still showed some level of desensitization, indicating that the desensitization of this receptor also has a phosphorylation-independent component. Indeed, the phosphorylation-dependent and phosphorylation-independent components are apparently additive, which could partly explain the higher degree of desensitization of apTKRPR-B than apTKRPR-A (Fig. 9, *D–E*). It is possible that vertebrate NK1Rs are like apTKRPRs and have multiple mechanisms for desensitization. If so, this could explain why there are apparently conflicting reports that describe the role of phosphorylation.

C-terminal phosphorylation-independent desensitization has been described in other receptors, such as the somatostatin receptor (111), the endotoxin B receptor (112), and mGluR1a (113). Possible mechanisms for densitization in these systems may include (1) phosphorylation of Ser/Thr residues on receptor intracellular loop 3 (114, 115); Indeed, there is one Ser residue at intracellular loop 3 of apTKRPR-A/apTKRPR-B (Fig. S6). (2) ligand binding, GPCR kinase binding, and arrestins phosphorylation–independent binding all may cause receptor transport (*e.g.*, endocytosis), leading to receptor uncoupling with G protein, and ultimately desensitization (111, 113).

Overall, our study provides a comprehensive description of the apTKRP signaling system in that it identifies the peptide precursor and two peptide receptor isoforms. We conducted structure-function analyses that highlight the importance of peptide PTMs and identified crucial amino acid residues for peptide bioactivity. Additionally, we examined mechanisms for apTKRPR desensitization, demonstrating that desensitization can occur through both phosphorylation-dependent and phosphorylation-independent pathways. This dual mechanism is likely conserved across species. Our findings on alternative splicing and desensitization of TKRPs in mollusk *Aplysia*, along with similar properties in vertebrate TK receptors, provides critical evidence for the evolutionary conservation of TKs and TKRP signaling systems.

Future research could continue this line of investigation and elucidate the molecular mechanisms underlying desensitization in more detail. For example, we could precisely map the phosphorylation sites of the receptors and investigate their specific roles in desensitization through detailed mutagenesis. We could also characterize potential GPCR kinases and arrestins in *Aplysia* (supporting results, Figs. S13–S20 and Table S7–S10) that might be involved in receptor desensitization. The roles of ser/thr residues in intracellular loops (Fig. S6) in desensitization, and possible mechanisms of C terminus of apTKRPR-A in inhibiting receptor activation as demonstrated in receptor truncations (Fig. 10, *C–E*), could be also explored. Finally, determining the specific distribution of each receptor type and identifying neural circuits containing receptors with different desensitization mechanisms could provide insights into functional impacts of desensitization, a poorly studied area in any system.

## Experimental procedures

### Subjects and reagents

Experiments were performed on *A. californica* (100–350 g) obtained from Marinus Scientific. Animals were maintained in an aquarium with a circulating artificial seawater at 14 to 16 °C, and the room was equipped with a 12:12 h light-dark cycle, with 6 AM to 6 PM as the daylight. Before dissection, animals were anesthetized by injection of isotonic 333 mm MgCl_2_ (about 50% of body weight) into the body cavity. All reagents were purchased from Sigma-Aldrich unless otherwise stated. Neuropeptides were synthesized in ChinaPeptides Co., Ltd and GuoPing Pharmaceutical Co, Ltd (Fig. S21).

### Bioinformatic analysis of peptide precursors and receptors

We used NCBI and AplysiaTools databases (Dr Thomas Abrams, University of Maryland) (70) to search specific sequences of interests. These latter databases (http://aplysiatools.org) include databases for *Aplysia* transcriptome and genome. The ORFs of the TKRP precursor and putative receptor cDNA sequences were obtained using ORF Finder (https://www.ncbi.nlm.nih.gov/orfnder/). For the TKRP precursor, the signal peptide was predicted using SignalP, 5.0 (http://www.cbs.dtu.dk/services/SignalP/), and the putative peptides encoded by the TKRP precursor were predicted using NeuroPred (http://stagbeetle.animal.uiuc.edu/cgi-bin/neuropred.py). For the putative TKRP receptors, TM domains and conserved sites were predicted using TMHMM Server, v. 2.0 (http://www.cbs.dtu.dk/services/TMHMM/) and NCBI Conserved Domain Database (http://www.ncbi.nlm.nih.gov/Structure/cdd/cdd.shtml). The phylogenetic trees were constructed by MEGA X software (https://www.megasofware.net/) using an alignment by Clustal W and the maximum likelihood method with 1000 replicates. The other parameters were set as default.

### RNA extraction and cDNA cloning

RNA was prepared from the *Aplysia* ganglia using the TRIzol reagent method. RNA quantity and purity were assessed by a Nanodrop ND-1000 spectrophotometer (Thermo Fisher Scientific). Using the above-extracted RNA as a template, cDNA was synthesized by reverse transcription using PrimeScript RT Master Mix Kit (TaKaRa) according to the instructions and then stored at −20 °C until use. The synthesized first-strand cDNA serves as a template for PCR. Gene-specific primers for PCR were designed based on protein-coding sequences for the apTKRP precursor and putative receptors (Table S1). The PCR reaction was performed with 98 °C/2 min predenaturing; 98 °C/10 s denaturing; ∼ 60 °C (depending on the specific primers: see Table S1)/15 s annealing; 72 °C/30 s extension; and 72 °C/5 min re-extension for 35 cycles. The PCR products were run on Agarose Gels, which were imaged with a Gel Image System (Tanon 1600). The expected PCR products were then purified using the MiniBEST Agarose Gel DNA Extraction Kit (TaKaRa) and subcloned into the pcDNA3.1(+) vector according to the manufacturer’s instructions and then were sequenced for confirmation.

### *In situ* hybridization

pSPT18 vector containing the partial sequence of apTKRP precursor was constructed for synthesizing sense or antisense RNA probes depending on the sequence insertion direction. PCR was performed to linearize the plasmid and amplify the insert, and then the amplified PCR product was purified using the MiniBEST Agarose Gel DNA Extraction Kit (TaKaRa). Sense and antisense digoxigenin (DIG)-labeled RNA probes were synthesized using a DIG RNA Labeling Kit (SP6/T7) (Roche).

The ganglia of *Aplysia* were dissected and fixed overnight at 4 °C with 4% paraformaldehyde in PBS. Ganglia were washed and dehydrated in an ascending ethanol series. After rehydration in a descending ethanol series, the ganglia were prehybridized at 50 °C for 6 h and hybridized overnight at 50 °C in hybridization buffer (50% formamide, 5 mM EDTA, 5× saline sodium citrate buffer, 1× Denhardt’s solution, 0.1% Tween 20, and 0.5 mg/ml yeast tRNA) containing 2 μg/ml DIG-labeled RNA probes. After washout of the probes, ganglia were then incubated overnight at 4 °C with a 1:200 dilution of alkaline phosphatase-conjugated anti-DIG antibody (Roche) in PBS containing 0.1% Tween 20, 0.2% bovine serum albumin (BSA), and 1% normal goat serum. After washing with PBS with Triton to remove unbound antibodies, ganglia were washed with detection buffer (100 mM NaCl, 50 mM MgCl_2_, 0.1% Tween 20, 1 mM levamisole, and 100 mM Tris–HCl, pH 9.5) and developed with 4.5 μl of nitro blue tetrazolium and 3.5 μl of 5-bromo-4-chloro-3-indolyl phosphate (Roche) in 1 ml of detection buffer. The staining reaction was monitored visually and stopped by washing with PBS with Triton when the level of staining was adequate. The stained ganglia were observed and photographed using a fluorescence microscope (Olympus) with epi-illumination against a white background. Photographs were taken with a Nikon CoolPix 990 digital camera.

### Immunohistochemistry

Immunohistochemistry was performed as described previously (116, 117). The primary antibody was raised against apTKRPG-DP in rabbit made by ChinaPeptides Co, Ltd, and to generate an immune response, apTKRPG-DP was coupled to keyhole limpet hemocyanin. For immunostaining, the tissue was fixed in a buffer (4% paraformaldehyde, 0.2% picric acid, 25% sucrose, and 0.1 M NaH_2_PO_4_, pH 7.6), for either 3 h at room temperature or overnight at 4 °C. All subsequent incubations were done at room temperature. The tissue was washed with PBSe (154 mM NaCl，20 mM PB (pH 7.6), and 1 mM EDTA-2Na, pH 8.0) and was permeabilized and blocked by overnight incubation in blocking buffer (10% normal goat serum, 2% Triton X-100, 1% BSA, 154 mM NaCl, 50 mM EDTA-2Na, and 10 mM Na_2_HPO_4_, pH 7.5). The primary antibody was diluted 1:500 in blocking buffer and incubated with the tissue for 4 to 7 days. The tissue was then washed twice per day for 2 to 3 days with washing buffer (2% Triton X-100, 1% BSA, 154 mM NaCl, 50 mM EDTA-2Na, and 10 mM Na_2_HPO_4_, pH 7.5). After washing, the tissue was incubated with a 1:500 dilution of secondary antibody (Alexa Fluor 594 goat anti-rabbit; Invitrogen: A-11012) in blocking buffer for 2 to 3 days and then washed again three times with washing buffer for 1 day and four times with storage buffer (1% BSA, 154 mM NaCl, 50 mM EDTA, and 10 mM Na_2_HPO_4_, pH 7.5) for 1 day. Finally, the tissue was observed and photographed under an Olympus fluorescence microscope.

To validate the specificity of the antibody, we performed preabsorption experiments with apTKRPG-DP following the same procedure above, with one exception. Before the primary antibody was incubated with the tissue, it was first incubated with 10^-4^ M apTKRPG-DP for 24 h.

### MS-based peptide sequencing and identification

Individual ganglia from the *Aplysia* central nervous system were homogenized in acidified methanol and processed for endogenous peptide extraction in preparation for MS similar to our prior approach (118, 119). Extracts were analyzed by LC-MS/MS using a Bruker nanoElute liquid chromatography hyphenated to a Bruker timsTOF Pro mass spectrometer *via* Bruker CaptiveSource. Extracts were separated using acetonitrile and water as solvents A and B, both with 0.1% formic acid, at a uniform flow rate of 300 nl/min at 40 °C. Solvent B gradient 2 to 40% was developed over 80 min, followed by wash and equilibration steps. Eluted peptides were measured in positive ion mode using the parallel accumulation serial fragmentation method with 1.1 s cycle time and 1/K_0_ mobility range of 0.6 to 1.6 V·s·cm^–2^.

Raw spectra data were imported into Peaks Online 11 (Bioinformatics Solutions) and peptide sequence tags were deduced *de novo* from the tandem mass spectra. Deduced tags were queried against a custom database of 131 *Aplysia* secreted proteins available on the NCBI website to map detected peptides to the precursor proteins. Matched peptides were scored by decoy-fusion method and reported with 1% false discovery rate cut-off (120, 121).

### Cell culture and transfection

CHO-K1 cells (Procell, CL-0062, validated by PCR) were cultured in F-12K medium (Gibco, 21127-022) with 10% fetal bovine serum (FBS) (Genial, G11-70500) at 37 °C in a humidified incubator containing 5% CO_2_. To express putative *Aplysia* receptors transiently in CHO-K1 cells, the cDNAs were subcloned into the pcDNA3.1(+) or pcDNA3.1(+)-FLAG (for ELISA experiments only, see “Cell surface expression analysis by ELISA” section) expression vector. On the day before transfection, the CHO-K1 cells were passaged into 60 mm diameter tissue culture–treated dishes, and the transfection experiments were performed when the cells were grown to 70 to 90% confluence. CHO-K1 cells were transfected with the cDNA plasmid constructs using Turbofect Transfection reagent (Thermo Fisher Scientific, R0531), following the manufacturer’s instructions. In brief, 4 μg of the putative receptor plasmids [in pcDNA3.1(+) or pcDNA3.1(+)-FLAG] were mixed with 400 μl of Opti-MEM (Gibco, 11058021), followed by the addition of 15 μl of TurboFect Transfection reagent and incubated at room temperature for 15 min. The DNA/TurboFect mixture dropwise was then added to the dish, and the cells were incubated at 37 °C in a humidified incubator containing 5% CO_2_ overnight.

### IP1 accumulation assay

The next day, transfected cells were trypsinized and reseeded in an opaque white 96-well half-area (Corning, 3688) or 384-well tissue culture–treated plates (Corning, 3570) at a density of 20,000 cells/well in F-12K and 10% FBS and incubated at 37 °C in a humidified incubator containing 5% CO_2_ overnight. On the third day, the activation of the putative receptors was detected by monitoring intracellular IP1 concentration using an IP-One Gq kit (Cisbio, 62IPAPEB) in Tecan Spark. Except for using 0.5x reagent, all other procedures were performed following the manufacturer’s instructions. IP1 accumulation assay measures the accumulation of IP1, which is hydrolyzed from the second messenger, and inositol triphosphate. Inositol triphosphate is generated by the Gαq pathway when a GPCR is activated by an appropriate ligand.

### Cell surface expression analysis by ELISA

Cell surface expression determination of apTKRPRs and mutant receptors, all with FLAG tags, was performed using a procedure modified from previous work (64, 122, 123, 124). Specifically, CHO-K1 cells were cultured in F-12K medium (Gibco, 21127-022) with 10% FBS (Genial, G11-70500) at 37 °C in a humidified incubator containing 5% CO_2_. When the cells were grown to 70 to 90% confluence, transfection experiments were performed (see “Cell culture and transfection” section). After 24 h, transfected cells were plated in 96-well white clear-bottom cell culture plates (Corning, 3610) at a density of 20,000 cells in 100 μl per well and incubated overnight. The following day, culture media was aspirated and cells were washed twice with 200 μl of 1× PBS (Procell, PB180327). Then, 100 μl of 1× PBS containing 5% (w/v) BSA was added to each well and incubated at room temperature. After 30 min, 100 μl of 1:10,000 anti-FLAG M2-horseradish peroxidase conjugate (Sigma-Aldrich, Cat A8592) was added to each well and incubated for 30 min at 37 °C. Cells were washed twice with 200 μl of 1× PBS and then incubated with 200 μl of TMB Chromogen Solution (Sangon Biotech, E661007) for 30 min at 37 °C in the dark. Finally, 50 μl of ELISA Stopping Solution (Sangon Biotech, E661006) was added to each well to stop the reaction. The absorbance at 450 nm was measured using a Tecan Spark microplate reader.

To assess cell surface expression of receptors postligand exposure (Fig. S12), we modified the above protocol as follows: after aspirating the culture media and washing the cells twice with 200 μl of 1× PBS (Procell, PB180327), the cells were cultured in F-12K media without FBS for 1 h. Subsequently, apTKRP-2b at a concentration of 10 μM was introduced to the wells of the experimental groups for varying durations. The cells were then washed twice with 200 μl of 1× PBS. Following this, each well received 100 μl of 1× PBS containing 5% (w/v) BSA and was incubated at room temperature. The subsequent procedures remained consistent with those described above.

### Receptor desensitization assay

A number of GPCRs exhibit homologous desensitization upon prolonged or repeated stimulation with an agonist. Therefore, we adopted two protocols to study the desensitization of apTKRPRs. The protocols were performed using a procedure modified from previous work (87, 88, 89).

Protocol 1 (87, 88): Under continuous (prolonged) stimulation with an agonist, we examined agonist-induced desensitization by quantifying the rate of IP1 accumulation. Specifically, CHO-K1 cells transiently expressing pcDNA3.1-apTKRPRs were incubated in the absence or presence of 10 μM (saturating concentration) apTKRP-2b for the different duration (2, 5, 10, 15, 30, and 60 min) at 37 °C, and intracellular concentrations of IP1 accumulation were measured. Data are presented as fold increase over basal IP1 concentration (Fig. S10).

Protocol 2 (89): To simulate repeated stimulation with an agonist, we examined agonist-induced desensitization by quantifying the accumulation level of IP1 after two applications of the agonist/ligand. Because the IP1 accumulation assay involves using LiCl to stop the degradation of IP1, in this protocol, LiCl was applied only during the second application of the agonist. Specifically, to elicit desensitization, cells were first exposed to 10 μM apTKRP-2b in the absence of LiCl for various pretreatment durations (pretreatment stage/phase Ⅰ). After this agonist exposure, cells were washed (three times) rapidly with PBS (phase Ⅱ), and the wash was consistently performed in each experiment. Responsiveness of the receptor was then assessed by measuring intracellular concentrations of IP1 accumulation in response to a second application of 10 μM apTKRP-2b for 15 min in the presence of LiCl (phase Ⅲ) (Fig. S11).

### Mutagenesis of apTKRPRs

Specifically, construction of the apTKRPR truncates and mutants were performed employing the full-length apTKRPR cDNAs cloned into the pcDNA3.1(+) (for IP1 accumulation and receptor desensitization assay) or pcDNA3.1(+)-FLAG (for cell surface expression analysis by ELISA) plasmid. Receptor truncation was performed using the premature termination in the C terminus. The length of tail deletion was selected to include clusters of Ser/Thr residues and possible phosphorylation sites. Site-directed mutagenesis was performed using the site-directed mutagenesis kit (Sangon Biotech), following the manufacturer’s instructions. Briefly, forward and reverse primers (Table S1) containing the expected mutation were mixed with kit components, and 10 ng of pcDNA3.1(+)-apTKRPR-A or pcDNA3.1(+)-apTKRPR-B (or with FLAG tag) was used as the mutation template. After 14 to 18 rounds of PCR amplification, 1 μl of DpnI was added and incubated at 37 °C for 1 h to digest the template. Mutants were confirmed *via* cDNA sequencing.

### Data and statistical analyses

Dose–response curves and bar graphs for experimental data were plotted using Prism software (GraphPad, https://www.graphpad.com/). Data are presented as mean ± SD of at least three independent experiments. Statistical tests were performed using Prism software. They included Student’s *t* test, and one-way or two-way ANOVA, as appropriate. Data that showed significant effects in ANOVA were further analyzed in individual comparisons with Bonferroni’s correction.

## Data availability

All data are included in this article and the Supporting information. The nucleotide sequences reported in this paper have been submitted to the GenBank/EBI Data Bank with accession number(s): PP823910 (*apTKRPR-A*), PP823911 (*apTKRPR-B*), PP823912 (*apTKRP* precursor).

## Supporting information

This article contains supporting information (24, 25, 26, 125, 126, 127).

## Conflict of interest

The authors declare that they have no conflicts of interest with the contents of this article.
