# Supplementary material for: Two C-terminal isoforms of Aplysia tachykinin–related peptide receptors exhibit phosphorylation-dependent and phosphorylation-independent desensitization mechanisms
Source: J Biol Chem. 2024 Jul 11;300(8):107556. doi: 10.1016/j.jbc.2024.107556 (PMC11365428; doi:10.1016/j.jbc.2024.107556)
Supplement: Figure S21 [file mmc11.pdf]

Contents

apTKRP-1 1

apTKRP-2a 4

apTKRP-2b 7

apTKRPG-DP 10

[Ala^{2}]apTKRP-2b 13

[Ala^{3}]apTKRP-2b 16

[Ala^{4}]apTKRP-2b 19

[Ala^{5}]apTKRP-2b 22

[Ala^{6}]apTKRP-2b 25

[Ala^{7}]apTKRP-2b_A6 28

[Ala^{8}]apTKRP-2b 31

[Ala^{9}]apTKRP-2b 34

[Ala^{10}]apTKRP-2b 37

apTKRP-2b-OH 40

apTKRP-1

安徽省国平药业有限公司

CERTIFICATE OF ANALYSIS

**Order ID**	GP120789-1
**Name**	N/A
**Lot No.**	GP120789-1-0818
**Sequence**	FKPSGFMGSR-NH2
**Dissolution condition**	100%H2O
**Length**	10AA
**Modification**	N/A
**Molecular Weight (MW)**	1112.30
**Storage**	-20°C

**Test Items**	**Specifications**	**Results**
**MW by MS**	1112.25	Conforms
**Purity by HPLC**	>95%	98.565%
**Peptide Content**	N/A	N/A
**Moisture content**	N/A	N/A
**Acetic acid content**	N/A	N/A
**Appearance**	White to off-white lyophilized powder	Conforms
**Quantity**	10mg	2.0mg*5

Certified by: LiuHui　　Date 08/28/2022

Quality Assurance Department

Note: this product is intended for research use only; not for diagnostic or human use.

Guoping Pharmaceutical Co., LTD

地址:合肥市经开区桃花工业园拓展区工投立恒工业广场A2西F1,电话:0551-62841987 传真:0551-62841765 www.guopingyaoye.com

Sample Information

Order ID :GP120789-1
Name :N/A
Sequence :FKPSGFMGSR-NH2
Lot.No :GP120789-1-0818
Pump A :0.1%Trifluoroacetic in 100% water
Pump B :0.1%Trifluoroacetic in 100% acetonitrile
Total Flow :1ml/min
Wavelength :220nm
Analytical column type :SHIMADZU Inertsil ODS-SP(4.6*250mm*5um)
Dissolution method :100%H2O
Inj. Volume :9 uL

Time	Module	Action	Value
0.01	Pumps	B.Conc	5
20.00	Pumps	B.Conc	45
23.00	Pumps	B.Conc	100
38.00	Pumps	B.Conc	100
40.00	Pumps	B.Conc	5
50.00	Controller	Stop	

Chromatogram

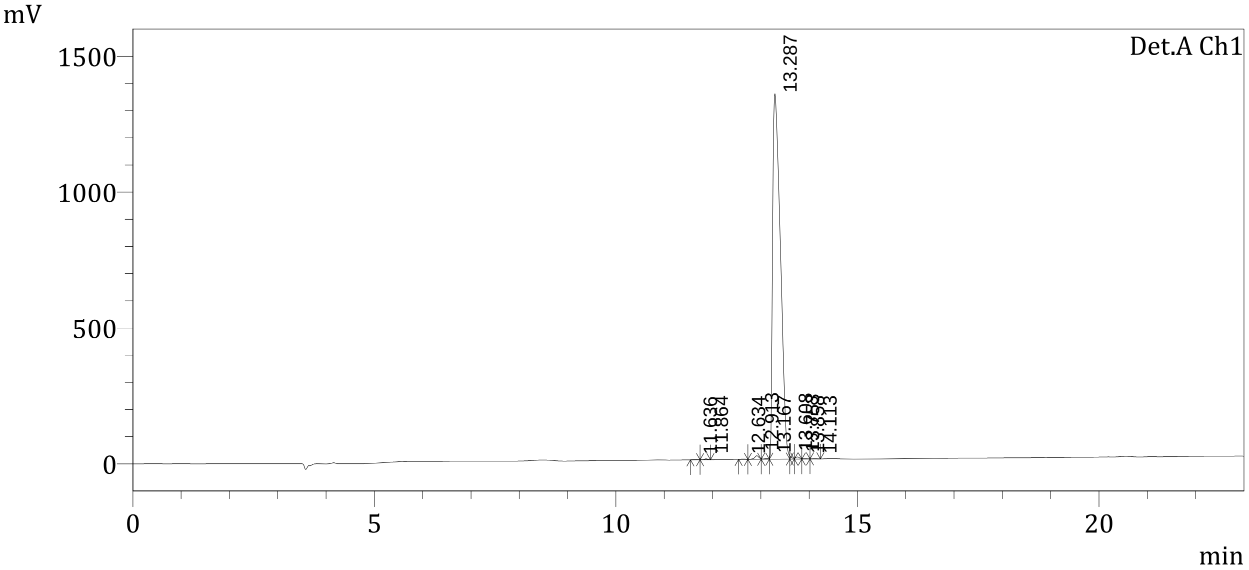

1 Det.A Ch1/220nm

PeakTable

Detector A Ch1 220nm

Peak#	Ret. Time	Area	Height	Area %	Height %
1	11.636	7230	1102	0.048	0.079
2	11.864	15535	3106	0.102	0.224
3	12.634	8047	1223	0.053	0.088
4	12.913	80184	12941	0.527	0.933
5	13.167	20759	3670	0.137	0.265
6	13.287	14982643	1344728	98.565	96.999
7	13.608	29425	10180	0.194	0.734
8	13.753	45216	7483	0.297	0.540
9	13.858	6412	1081	0.042	0.078
10	14.113	5351	822	0.035	0.059
Total		15200801	1386337	100.000	100.000

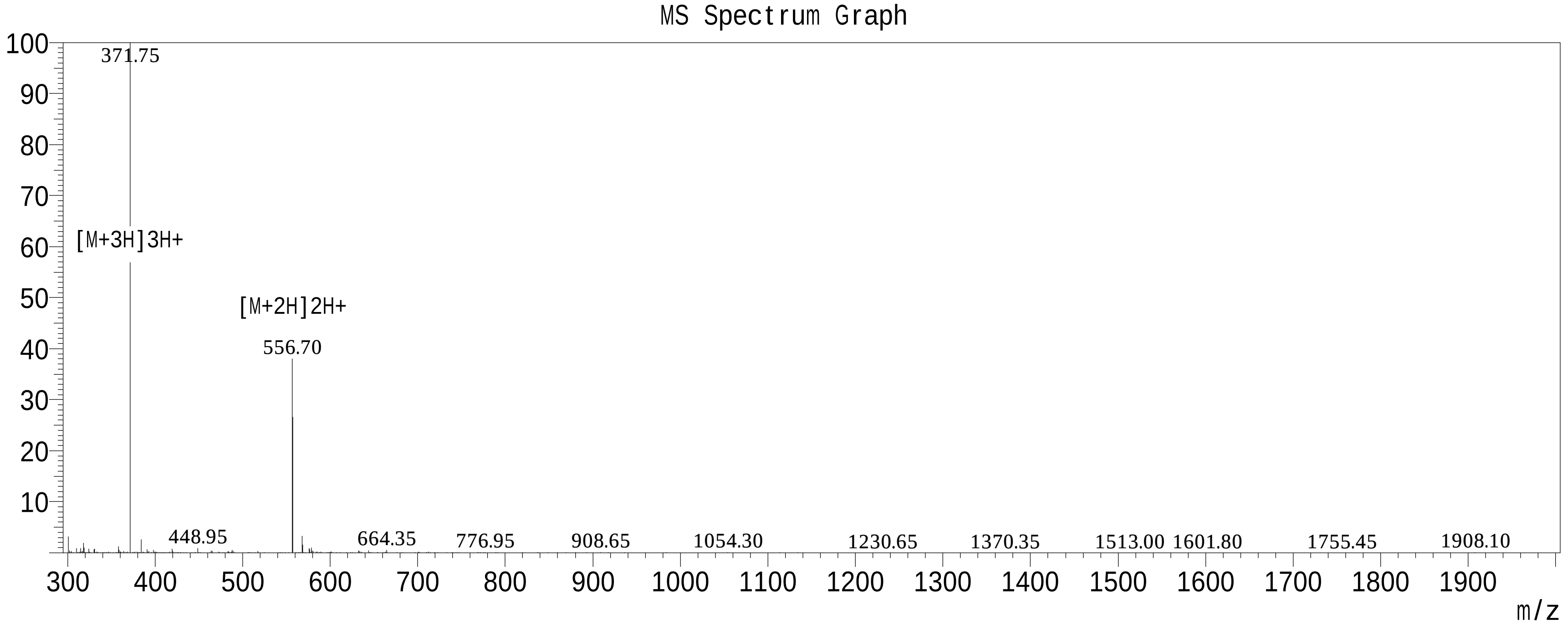

Sample Information

Dissolution method	:5%HAC+8%ACN+87%H2O	Interface	:ESI	Prerod Bias	:+1.5kv
Modified Date	:2022/08/28	Nebulizing Gas Flow	:1.50L/min	Detector	:-0.2kv
Injection Volume	:1ul	CDL Temp	:250C	T.Flow	:0.2ml/min
Heat Block Temp	:200	CDL Volt	:0v	B.conc	:50%H2O/50%MEOH

Order ID :GP120789-1
Name :N/A
Sequence :FKPSGFMGSR-NH2
Lot.No :GP120788-1-0817
Theoretical :1112.30
Observed :1112.25

apTKRP-2a

安徽省国平药业有限公司

CERTIFICATE OF ANALYSIS

**Order ID**	GP120789-2
**Name**	N/A
**Lot No.**	GP120789-2-0818
**Sequence**	QPHLGFHGSR-NH2
**Dissolution condition**	100%H2O
**Length**	10AA
**Modification**	N/A
**Molecular Weight (MW)**	1134.26
**Storage**	-20°C

**Test Items**	**Specifications**	**Results**
**MW by MS**	1133.80	Conforms
**Purity by HPLC**	>95%	96.659%
**Peptide Content**	N/A	N/A
**Moisture content**	N/A	N/A
**Acetic acid content**	N/A	N/A
**Appearance**	White to off-white lyophilized powder	Conforms
**Quantity**	10mg	2.0mg*5

Certified by: LiuHui　　Date 08/28/2022

Quality Assurance Department

Note: this product is intended for research use only; not for diagnostic or human use.

Guoping Pharmaceutical Co., LTD

地址:合肥市经开区桃花工业园拓展区工投立恒工业广场A2西F1,电话:0551-62841987 传真:0551-62841765 www.guopingyaoye.com

Sample Information

Order ID	:GP120789-2		
Name	:N/A		
Sequence	:QPHLGFHGSR-NH2		
Lot.No	:GP120789-2-0818		
Pump A	:0.1%Trifluoroacetic in 100% water		
Pump B	:0.1%Trifluoroacetic in 100% acetonitrile		
Total Flow	:1ml/min		
Wavelength	:220nm		
Analytical column type	:SHIMADZU Inertsil ODS-SP(4.6*250mm*5um)		
Dissolution method	:100%H2O		
Inj. Volume	:11 uL		
Time	Module	Action	Value
0.01	Pumps	B.Conc	5
20.00	Pumps	B.Conc	45
23.00	Pumps	B.Conc	100
38.00	Pumps	B.Conc	100
40.00	Pumps	B.Conc	5
50.00	Controller	Stop	

Chromatogram

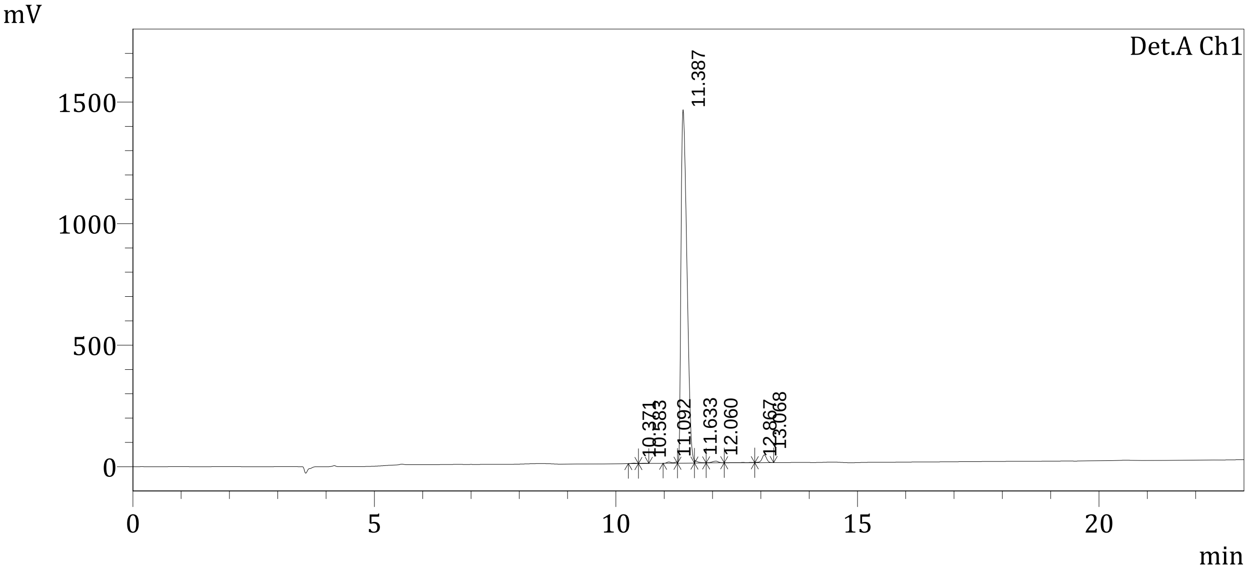

1 Det.A Ch1/220nm

PeakTable

Detector A Ch1 220nm

Peak#	Ret. Time	Area	Height	Area %	Height %
1	10.371	11352	1815	0.089	0.120
2	10.583	7585	1117	0.060	0.074
3	11.092	52419	5373	0.411	0.355
4	11.387	12314067	1453778	96.659	96.129
5	11.633	49697	10086	0.390	0.667
6	12.060	64569	7240	0.507	0.479
7	12.867	24177	1057	0.190	0.070
8	13.068	215824	31850	1.694	2.106
Total		12739690	1512316	100.000	100.000

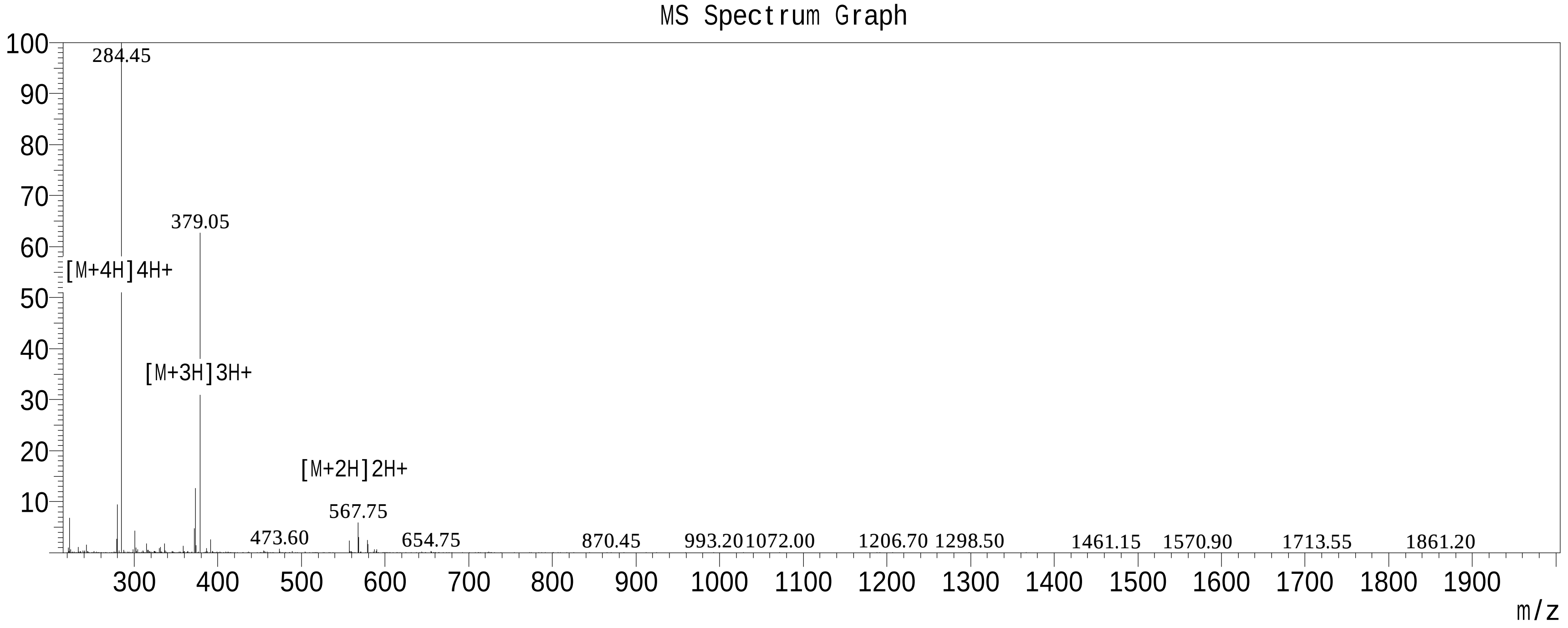

Sample Information

Dissolution method	:5%HAC+8%ACN+87%H2O	Interface	:ESI	Prerod Bias	:+1.5kv
Modified Date	:2022/08/28	Nebulizing Gas Flow	:1.50L/min	Detector	:-0.2kv
Injection Volume	:1ul	CDL Temp	:250C	T.Flow	:0.2ml/min
Heat Block Temp	:200	CDL Volt	:0v	B.conc	:50%H2O/50%MEOH

Order ID	:GP120789-2
Name	:N/A
Sequence	:QPHLGFHGSR-NH2
Lot.No	:GP120789-2-0818
Theoretical	:1134.26
Observed	:1133.80

安徽省国平药业有限公司

CERTIFICATE OF ANALYSIS

**Order ID**	GP120789-3
**Name**	N/A
**Lot No.**	GP120789-3-0818
**Sequence**	[p-]QPHLGFHGSR-NH2
**Dissolution condition**	100%H2O
**Length**	10AA
**Modification**	N/A
**Molecular Weight (MW)**	1117.23
**Storage**	-20°C

**Test Items**	**Specifications**	**Results**
**MW by MS**	1117.20	Conforms
**Purity by HPLC**	>95%	99.401%
**Peptide Content**	N/A	N/A
**Moisture content**	N/A	N/A
**Acetic acid content**	N/A	N/A
**Appearance**	White to off-white lyophilized powder	Conforms
**Quantity**	10mg	2.0mg*5

Certified by: LiuHui Date 08/28/2022

Quality Assurance Department

Note: this product is intended for research use only; not for diagnostic or human use.

Guoping Pharmaceutical Co., LTD

地址:合肥市经开区桃花工业园拓展区工投立恒工业广场A2西F1,电话:0551-62841987 传真:0551-62841765 www.guopingyaoye.com

Sample Information

Order ID :GP120789-3
Name :N/A
Sequence :[p-]QPHLGFHGSR-NH2
Lot.No :GP120789-3-0818
Pump A :0.1%Trifluoroacetic in 100% water
Pump B :0.1%Trifluoroacetic in 100% acetonitrile
Total Flow :1ml/min
Wavelength :220nm
Analytical column type :SHIMADZU Inertsil ODS-SP(4.6*250mm*5um)
Dissolution method :100%H2O
Inj. Volume :12 uL

Time	Module	Action	Value
0.01	Pumps	B.Conc	10
20.00	Pumps	B.Conc	50
23.00	Pumps	B.Conc	100
38.00	Pumps	B.Conc	100
40.00	Pumps	B.Conc	10
50.00	Controller	Stop	

Chromatogram

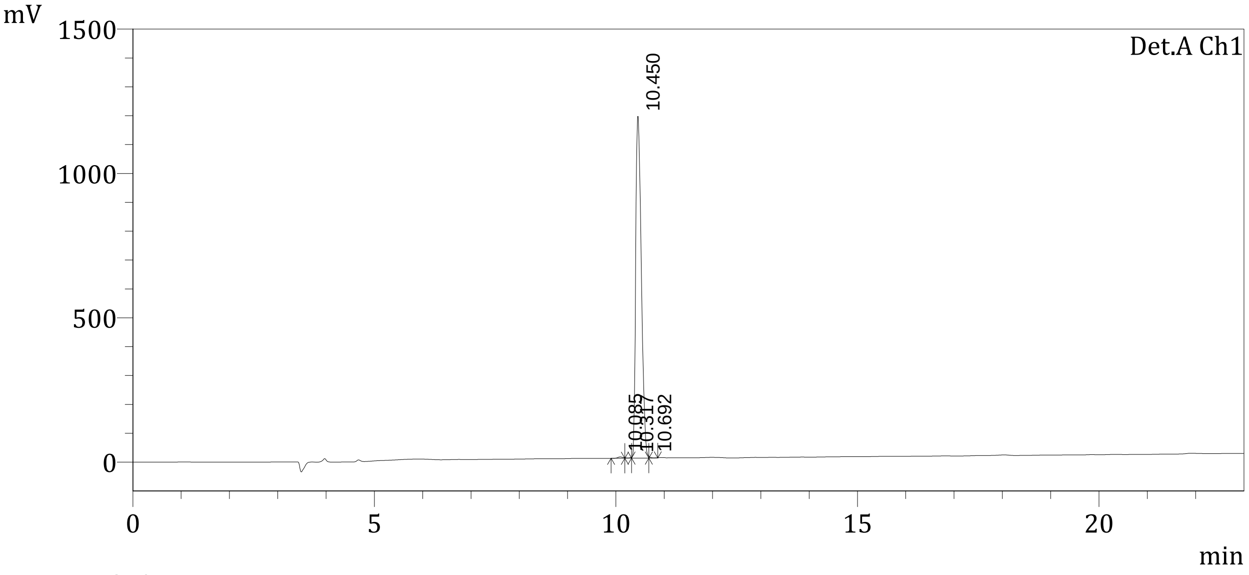

1 Det.A Ch1/220nm

PeakTable

Detector A Ch1 220nm

Peak#	Ret. Time	Area	Height	Area %	Height %
1	10.085	36100	4949	0.378	0.414
2	10.317	11817	2160	0.124	0.181
3	10.450	9484593	1183913	99.401	99.083
4	10.692	9276	3843	0.097	0.322
Total		9541786	1194866	100.000	100.000

MS Spectrum Graph

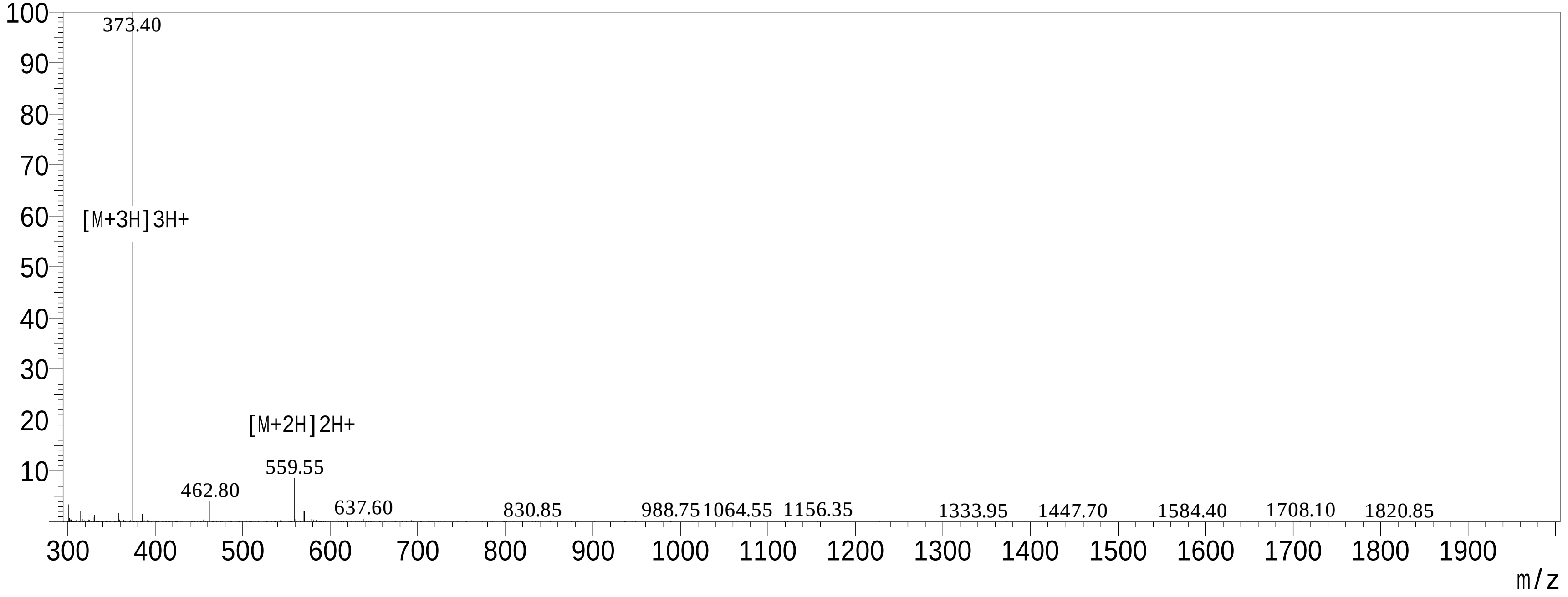

Sample Information

Dissolution method	:5%HAC+8%ACN+87%H2O	Interface	:ESI	Prerod Bias	:+1.5kv
Modified Date	:2022/08/28	Nebulizing Gas Flow	:1.50L/min	Detector	:-0.2kv
Injection Volume	:1ul	CDL Temp	:250C	T.Flow	:0.2ml/min
Heat Block Temp	:200	CDL Volt	:0v	B.conc	:50%H2O/50%MEOH

Order ID	:GP120789-3
Name	:N/A
Sequence	:[p-]QPHLGFHGSR-NH2
Lot.No	:GP120789-3-0818
Theoretical	:1117.23
Observed	:1117.20

Certificate of Analysis

Product Name: 04010054501

Sequence: FMPGLESLLLAKYYQDAKW

Sequence(Three Letters Code): Phe-Met-Pro-Gly-Leu-Glu-Ser-Leu-Leu-Leu-Ala-Lys-Tyr-Tyr-Gln-Asp-Ala-Lys-Trp

Purity: 96.34%

Molecular Weight: 2273.68

Solubility: 1mg/ml in 25%ACN/75%H_2O

Test	Specification	Result
**Purity:**	HPLC	Conforms
	(See attached RP-HPLC chromatogram)	
**MS Analysis:**	ESI-MS	Conforms
	(See attached MS spectrum)	
**Counter Ion:**	Trifluoroacetate	Conforms
**Appearance:**	Lyophilized powder or Crystallization	Conforms

Quality Assurance By: ____________ **Position**: Manager **Date**: 2020-09-10

Important: The peptides can be used for research only. Most of the peptides are lyophilized white or faint yellow powder while fluorescent modified ones have special colors. The state of peptides with strong hydrophilic properties may be crystalline or liquid which does not affect for use. Before experiment, please choose proper solvent for your experiment to dissolve peptides. If peptides cannot be dissolved under harsh conditions, we can carry out feasibility study. Storage conditions:-20ºC, seal, avoid light, dry.

Please test the sample within two weeks after receiving it.

注意：本品仅供科研，生产用途，不得直接用于人体。

HPLC Analysis Report

Measurement:	Peak Area	Run Time:	20min
Calculation Type:	Percent	Wavelength :	220nm
Flow Rate :	1.0ml/min	Inj.Vol:	10uL
Column:	Kromasil 100-5C18,4.6mmX250mm,5 micron	Column Temp:	30℃
Buffer A :	0.1%TFA in Acetonitrile	Buffer B:	0.1%TFA in water

Gradient(linear):		A	B
	0.0min	31%	69%
	20min	56%	44%
	20.1min	100%	0%

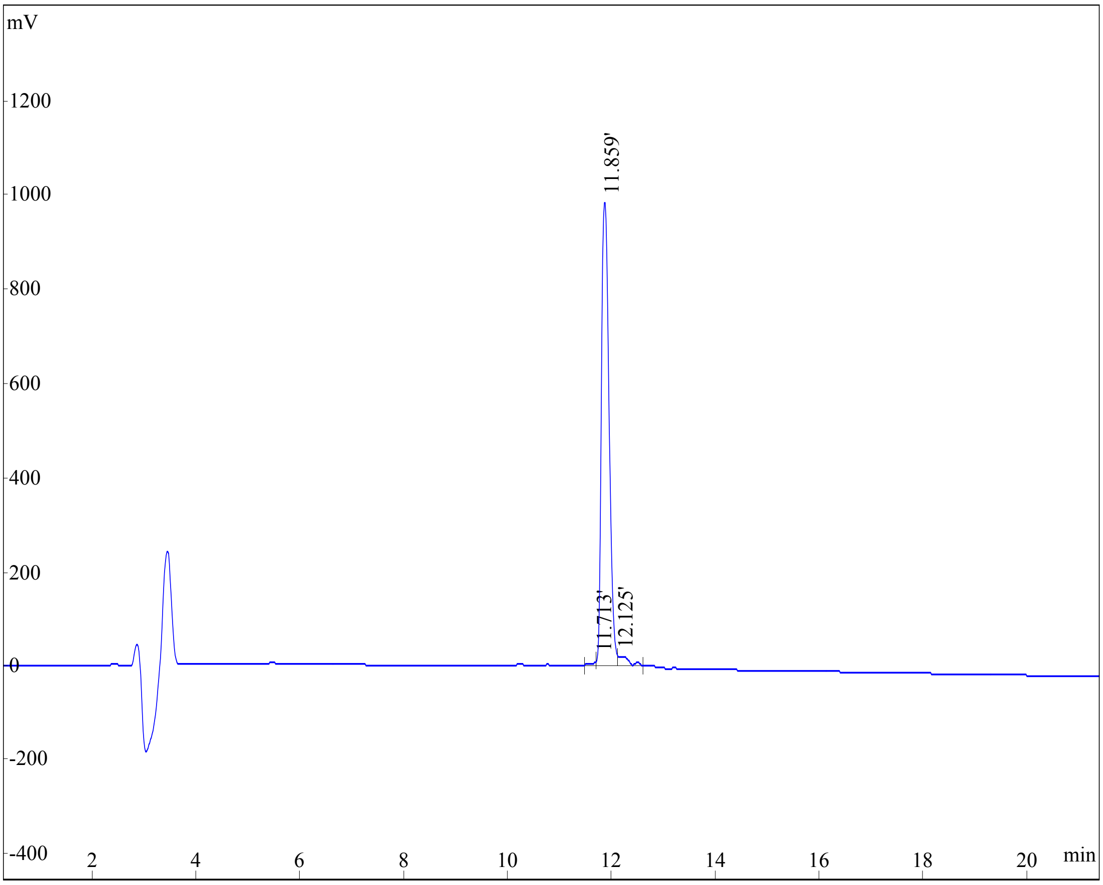

Rank	Time	Name	Conc.	Area
1	11.713		0.4236	40579
2	11.859		96.34	9227514
3	12.125		3.238	310157
Total			100	9578250

MS Analysis Report

Ion Source: ESI   Capillary(KV):±(2500~3000)

Desolvation(L/hr):800   Desolvation Temp:450℃

Cone(V): 30~50   Run Time: 1min

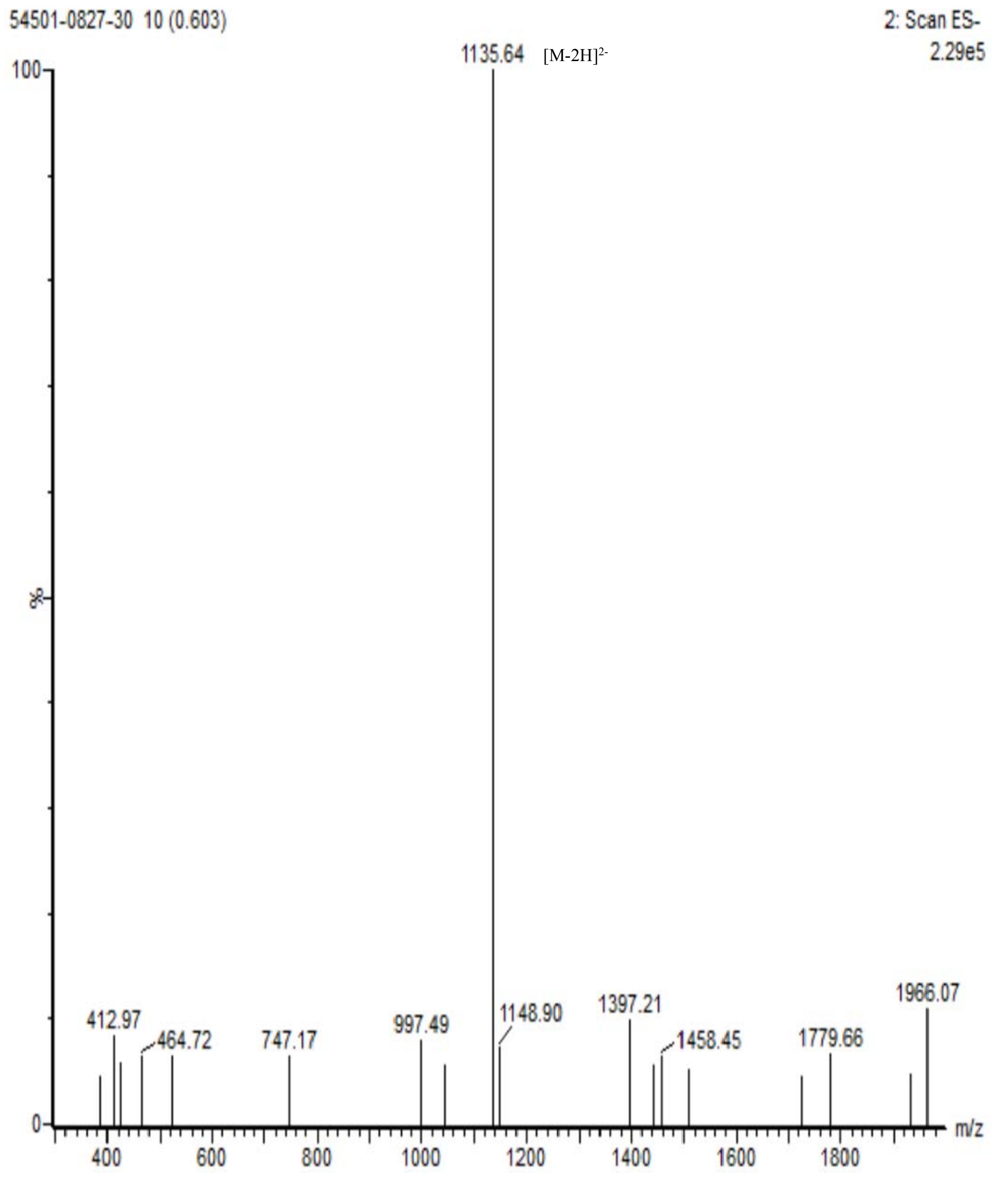

Certificate of Analysis

Product Name: [Ala²]apTKRP-2b

Sequence: [p-]QAHLGFHGSR-NH2

Sequence(Three Letters Code): [p-]Gln-Ala-His-Leu-Gly-Phe-His-Gly-Ser-Arg-NH2

Purity: 98.39%

Molecular Weight: 1091.21

Solubility: 1mg/ml in 15%ACN/85%H_2O

Test	Specification	Result
**Purity:**	HPLC	Conforms
	(See attached RP-HPLC chromatogram)	
**MS Analysis:**	ESI-MS	Conforms
	(See attached MS spectrum)	
**Counter Ion:**	Trifluoroacetate	Conforms
**Appearance:**	Lyophilized powder or Crystallization	Conforms

Quality Assurance By: ____________ **Position**: Manager **Date**: 2022-06-17

Important: The peptides can be used for research only. Most of the peptides are lyophilized white or faint yellow powder while fluorescent modified ones have special colors. The state of peptides with strong hydrophilic properties may be crystalline or liquid which does not affect for use. Before experiment, please choose proper solvent for your experiment to dissolve peptides. If peptides cannot be dissolved under harsh conditions, we can carry out feasibility study. Storage conditions:-20ºC, seal, avoid light, dry.

Please test the sample within two weeks after receiving it.

注意：本品仅供科研，生产用途，不得直接用于人体。

HPLC Analysis Report

Measurement:	Peak Area	Run Time:	20min
Calculation Type:	Percent	Wavelength :	220nm
Flow Rate :	1.0ml/min	Inj.Vol:	10uL
Column:	Kromasil 100-5C18,4.6mmX250mm,5 micron	Column Temp:	25℃
Buffer A :	0.1%TFA in Acetonitrile	Buffer B:	0.1%TFA in water

Gradient(linear):		A	B
	0.0min	17%	83%
	20min	42%	58%
	20.1min	100%	0%

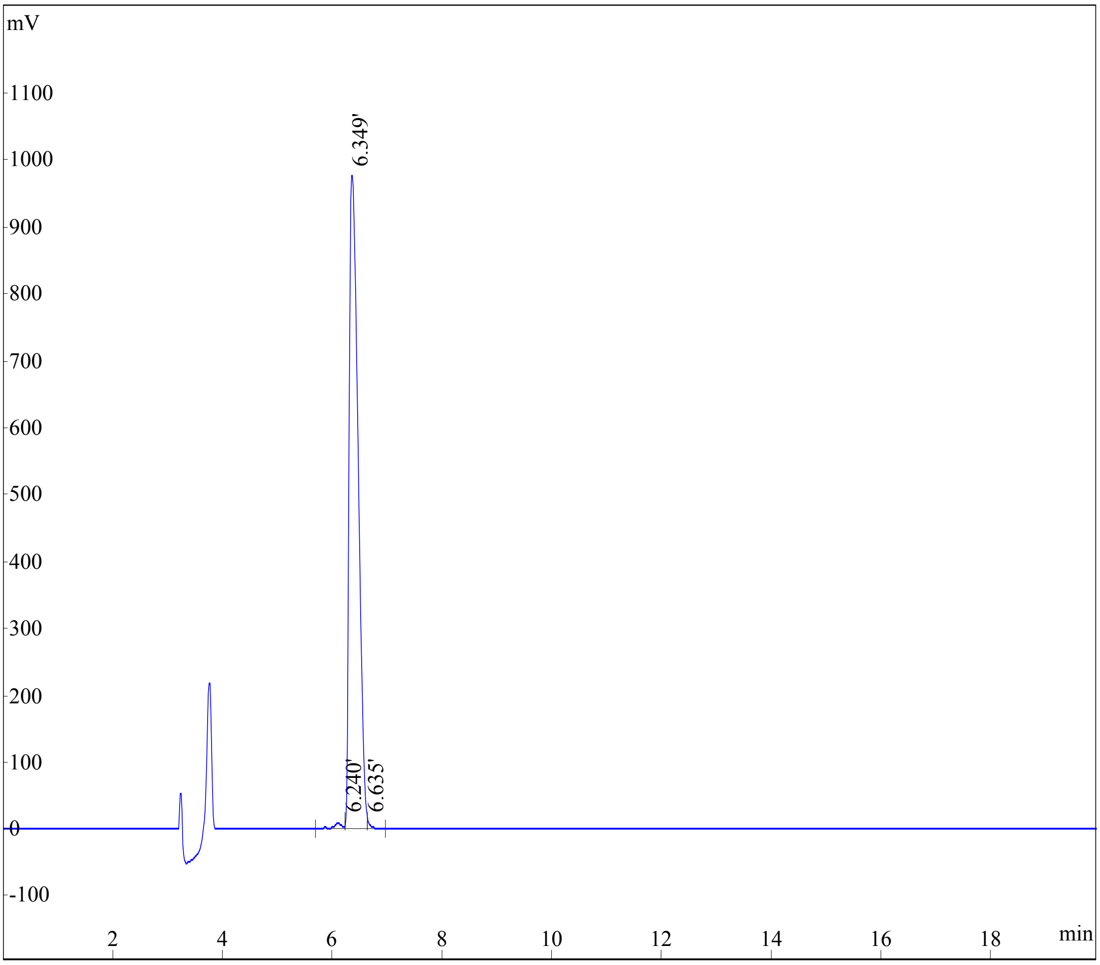

Rank	Time	Name	Conc.	Area
1	6.240		1.144	119790
2	6.349		98.39	10301319
3	6.635		0.4669	48880
Total			100	10469989

MS Analysis Report

Ion Source: ESI Capillary(KV):±(2500~3500)

Desolvation(L/hr):800 Desolvation Temp:450℃

Cone(V): 15~30 Run Time: 1min

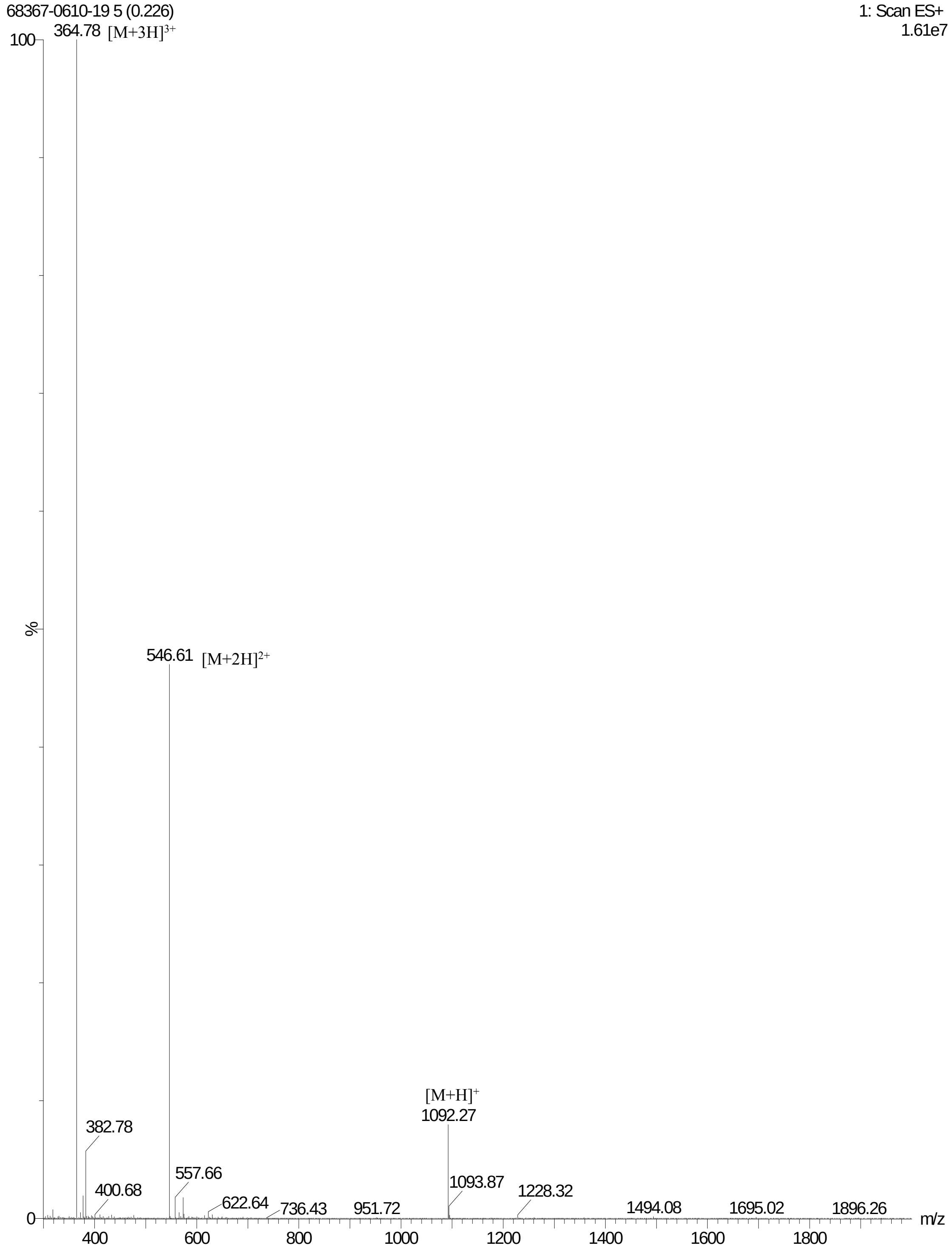

Certificate of Analysis

Product Name: [Ala^3]apTKRP-2b

Sequence: [p-]QPALGFHGSR-NH2

Sequence(Three Letters Code): [p-]Gln-Pro-Ala-Leu-Gly-Phe-His-Gly-Ser-Arg-NH2

Purity: 97.31%

Molecular Weight: 1051.18

Solubility: 1mg/ml in 15%ACN/85%H_2O

**Test**	**Specification**	**Result**
**Purity:**	HPLC	Conforms
	(See attached RP-HPLC chromatogram)	
**MS Analysis:**	ESI-MS	Conforms
	(See attached MS spectrum)	
**Counter Ion:**	Trifluoroacetate	Conforms
**Appearance:**	Lyophilized powder or Crystallization	Conforms

Quality Assurance By: ____________ **Position**: Manager **Date**: 2022-06-17

Important: The peptides can be used for research only. Most of the peptides are lyophilized white or faint yellow powder while fluorescent modified ones have special colors. The state of peptides with strong hydrophilic properties may be crystalline or liquid which does not affect for use. Before experiment, please choose proper solvent for your experiment to dissolve peptides. If peptides cannot be dissolved under harsh conditions, we can carry out feasibility study. Storage conditions:-20ºC, seal, avoid light, dry.

Please test the sample within two weeks after receiving it.

注意：本品仅供科研，生产用途，不得直接用于人体。

HPLC Analysis Report

Measurement:	Peak Area	Run Time:	20min
Calculation Type:	Percent	Wavelength :	220nm
Flow Rate :	1.0ml/min	Inj.Vol:	10uL
Column:	Kromasil 100-5C18,4.6mmX250mm,5 micron	Column Temp:	25℃
Buffer A :	0.1%TFA in Acetonitrile	Buffer B:	0.1%TFA in water

Gradient(linear):		A	B
	0.0min	17%	83%
	20min	42%	58%
	20.1min	100%	0%

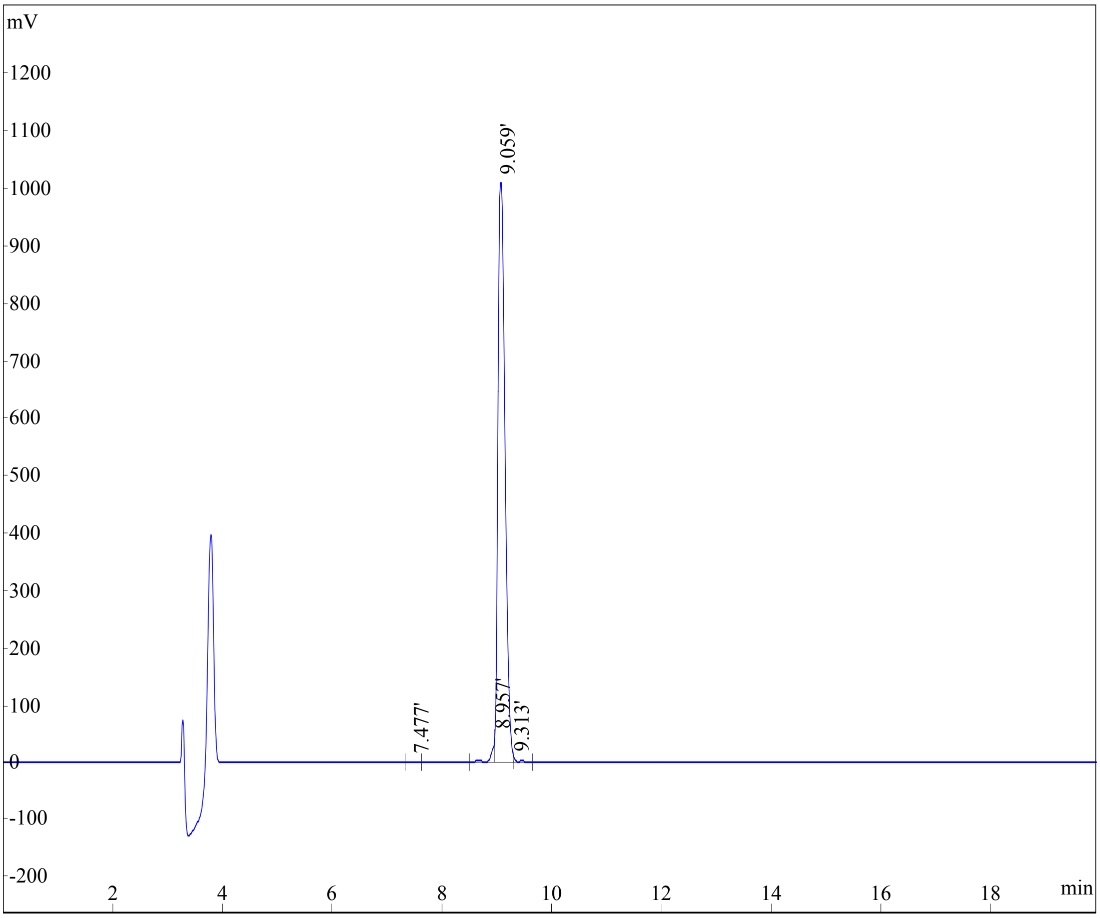

Rank	Time	Name	Conc.	Area
1	7.477		0.08925	7570
2	8.957		2.124	180131
3	9.059		97.31	8253283
4	9.313		0.4757	40347
Total			100	8481331

MS Analysis Report

Ion Source: ESI Capillary(KV):±(2500~3500)

Desolvation(L/hr):800 Desolvation Temp:450℃

Cone(V): 15~30 Run Time: 1min

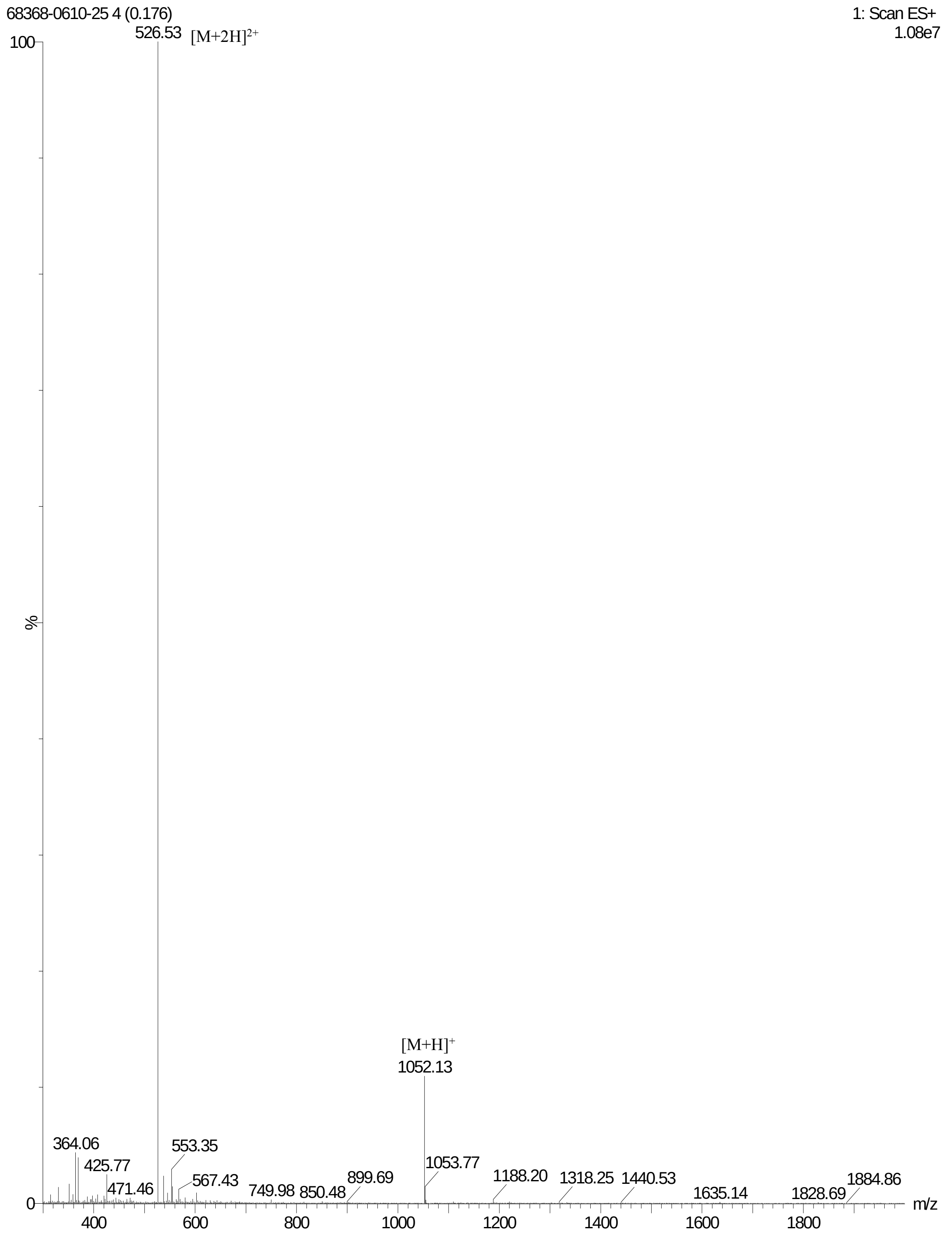

Certificate of Analysis

Product Name: [Ala4]apTKRP-2b

Sequence: [p-]QPHAGFHGSR-NH2

Sequence(Three Letters Code): [p-]Gln-Pro-His-Ala-Gly-Phe-His-Gly-Ser-Arg-NH2

Purity: 97.41%

Molecular Weight: 1075.16

Solubility: 1mg/ml in 13%ACN/87%H_2O

Test	Specification	Result
**Purity:**	HPLC	Conforms
	(See attached RP-HPLC chromatogram)	
**MS Analysis:**	ESI-MS	Conforms
	(See attached MS spectrum)	
**Counter Ion:**	Trifluoroacetate	Conforms
**Appearance:**	Lyophilized powder or Crystallization	Conforms

Quality Assurance By: ____________ **Position**: Manager **Date**: 2022-06-17

Important: The peptides can be used for research only. Most of the peptides are lyophilized white or faint yellow powder while fluorescent modified ones have special colors. The state of peptides with strong hydrophilic properties may be crystalline or liquid which does not affect for use. Before experiment, please choose proper solvent for your experiment to dissolve peptides. If peptides cannot be dissolved under harsh conditions, we can carry out feasibility study. Storage conditions:-20ºC, seal, avoid light, dry.

Please test the sample within two weeks after receiving it.

注意：本品仅供科研，生产用途，不得直接用于人体。

HPLC Analysis Report

Measurement:	Peak Area	Run Time:	20min
Calculation Type:	Percent	Wavelength :	220nm
Flow Rate :	1.0ml/min	Inj.Vol:	10uL
Column:	Kromasil 100-5C18,4.6mmX250mm,5 micron	Column Temp:	25℃
Buffer A :	0.1%TFA in Acetonitrile	Buffer B:	0.1%TFA in water

Gradient(linear):		A	B
	0.0min	12%	88%
	20min	37%	63%
	20.1min	100%	0%

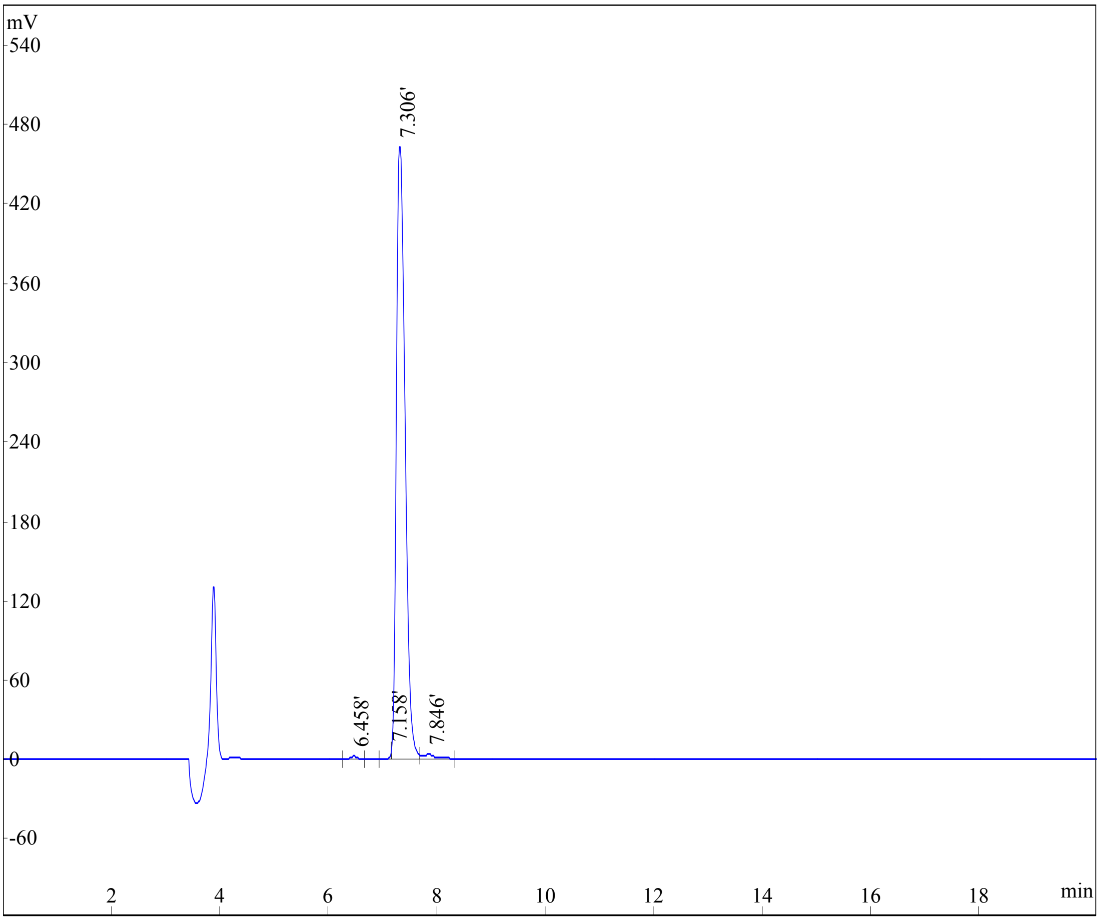

Rank	Time	Name	Conc.	Area
1	6.458		0.5063	24977
2	7.158		0.2903	14318
3	7.306		97.41	4804918
4	7.846		1.797	88622
Total			100	4932835

MS Analysis Report

Ion Source: ESI Nebulizer Gas (NEB): 12.00

Curtain Gas(CUR): 6.00 Ionspray Voltage(IS): ±4500

Temperature(TEM): 0.00 Run Time: 0.5-1min

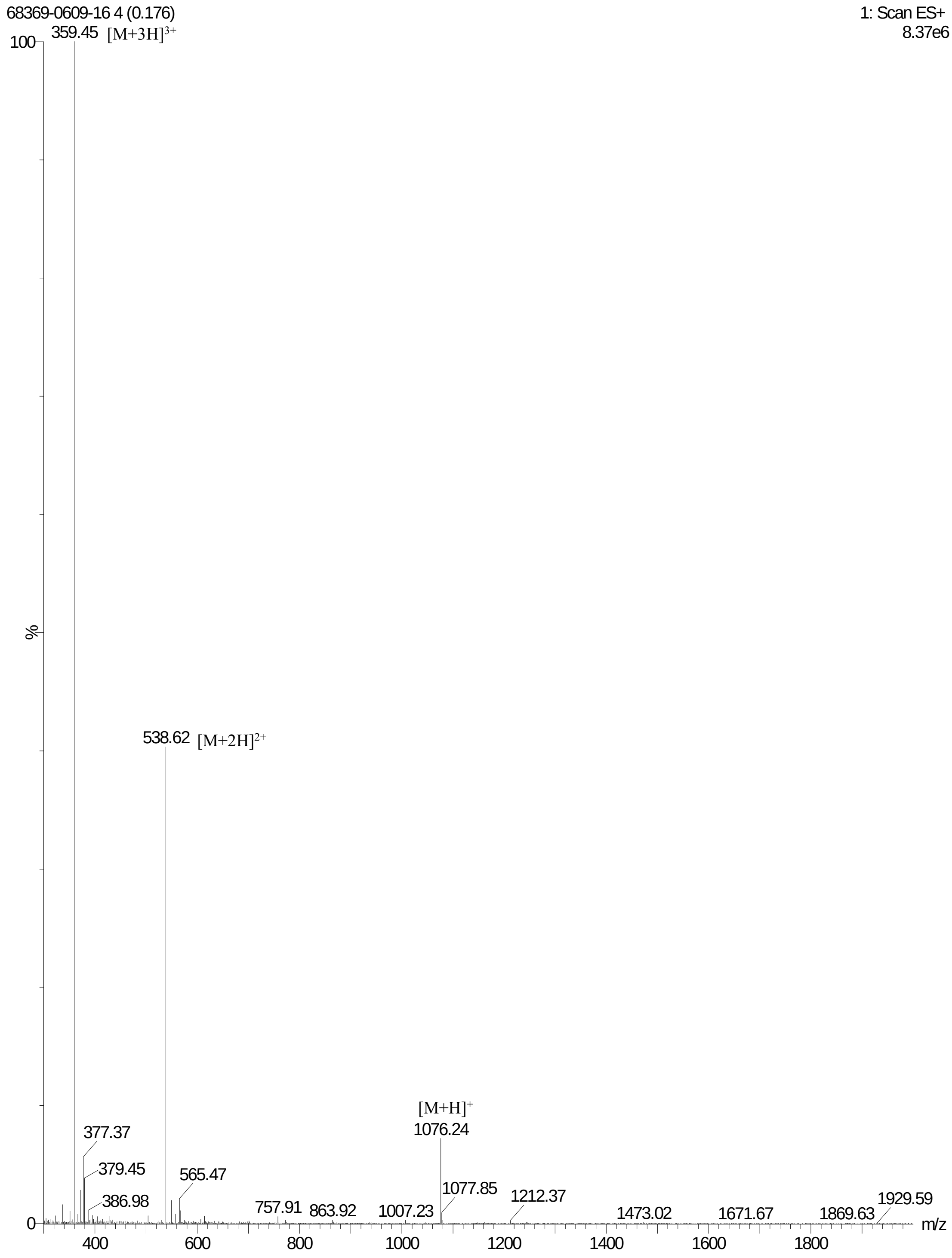

Certificate of Analysis

Product Name: [Ala^5]apTKRP-2b

Sequence: [p-]QPHLAFHGSR-NH2

Sequence(Three Letters Code): [p-]Gln-Pro-His-Leu-Ala-Phe-His-Gly-Ser-Arg-NH2

Purity: 97.64%

Molecular Weight: 1131.27

Solubility: 1mg/ml in 15%ACN/85%H_2O

Test	Specification	Result
**Purity:**	HPLC	Conforms
	(See attached RP-HPLC chromatogram)	
**MS Analysis:**	ESI-MS	Conforms
	(See attached MS spectrum)	
**Counter Ion:**	Trifluoroacetate	Conforms
**Appearance:**	Lyophilized powder or Crystallization	Conforms

Quality Assurance By: ____________ **Position**: Manager **Date**: 2022-06-17

Important: The peptides can be used for research only. Most of the peptides are lyophilized white or faint yellow powder while fluorescent modified ones have special colors. The state of peptides with strong hydrophilic properties may be crystalline or liquid which does not affect for use. Before experiment, please choose proper solvent for your experiment to dissolve peptides. If peptides cannot be dissolved under harsh conditions, we can carry out feasibility study. Storage conditions:-20ºC, seal, avoid light, dry.

Please test the sample within two weeks after receiving it.

注意：本品仅供科研，生产用途，不得直接用于人体。

HPLC Analysis Report

Measurement:	Peak Area	Run Time:	20min
Calculation Type:	Percent	Wavelength :	220nm
Flow Rate :	1.0ml/min	Inj.Vol:	10uL
Column:	Kromasil 100-5C18,4.6mmX250mm,5 micron	Column Temp:	25℃
Buffer A :	0.1%TFA in Acetonitrile	Buffer B:	0.1%TFA in water

Gradient(linear):		A	B
	0.0min	17%	83%
	20min	42%	58%
	20.1min	100%	0%

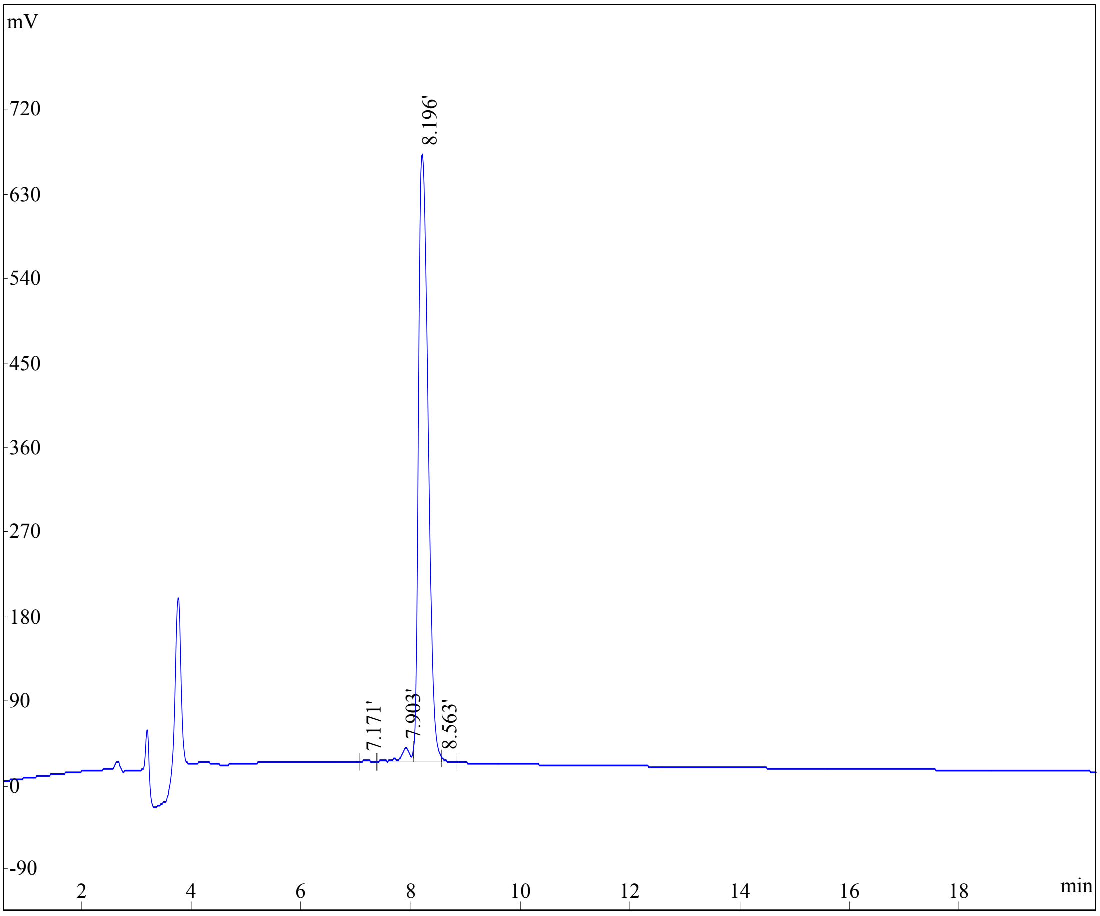

Rank	Time	Name	Conc.	Area
1	7.171		0.08702	6531
2	7.903		2.114	158702
3	8.196		97.64	7328738
4	8.563		0.1592	11946
Total			100	7505917

MS Analysis Report

Ion Source: ESI　　Capillary(KV):±(2500~3500)

Desolvation(L/hr):800　　Desolvation Temp:450℃

Cone(V): 15~30　　Run Time: 1min

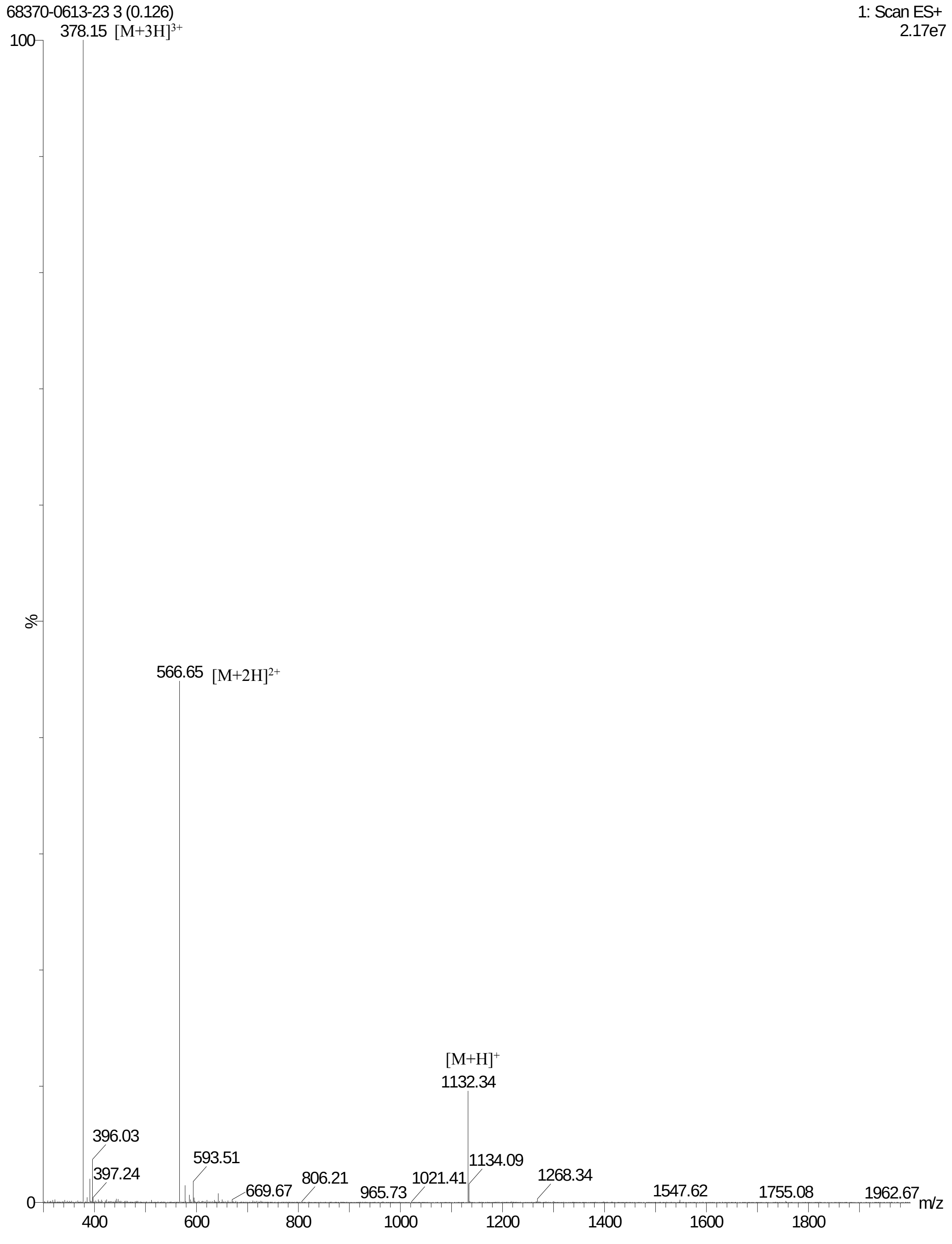

Certificate of Analysis

Product Name: [Ala^6]apTKRP-2b

Sequence: [p-]QPHLGAHGSR-NH2

Sequence(Three Letters Code): [p-]Gln-Pro-His-Leu-Gly-Ala-His-Gly-Ser-Arg-NH2

Purity: 96.20%

Molecular Weight: 1041.15

Solubility: 1mg/ml in 13%ACN/87%H_2O

Test	Specification	Result
**Purity:**	HPLC	Conforms
	(See attached RP-HPLC chromatogram)	
**MS Analysis:**	ESI-MS	Conforms
	(See attached MS spectrum)	
**Counter Ion:**	Trifluoroacetate	Conforms
**Appearance:**	Lyophilized powder or Crystallization	Conforms

Quality Assurance By: ____________ **Position**: Manager **Date**: 2022-06-17

Important: The peptides can be used for research only. Most of the peptides are lyophilized white or faint yellow powder while fluorescent modified ones have special colors. The state of peptides with strong hydrophilic properties may be crystalline or liquid which does not affect for use. Before experiment, please choose proper solvent for your experiment to dissolve peptides. If peptides cannot be dissolved under harsh conditions, we can carry out feasibility study. Storage conditions:-20℃, seal, avoid light, dry.

Please test the sample within two weeks after receiving it.

注意：本品仅供科研，生产用途，不得直接用于人体。

HPLC Analysis Report

Measurement:	Peak Area	Run Time:	20min
Calculation Type:	Percent	Wavelength :	220nm
Flow Rate :	1.0ml/min	Inj.Vol:	10uL
Column:	Kromasil 100-5C18,4.6mmX250mm,5 micron	Column Temp:	25℃
Buffer A :	0.1%TFA in Acetonitrile	Buffer B:	0.1%TFA in water

Gradient(linear):

	A	B
0.0min	12%	88%
20min	37%	63%
20.1min	100%	0%

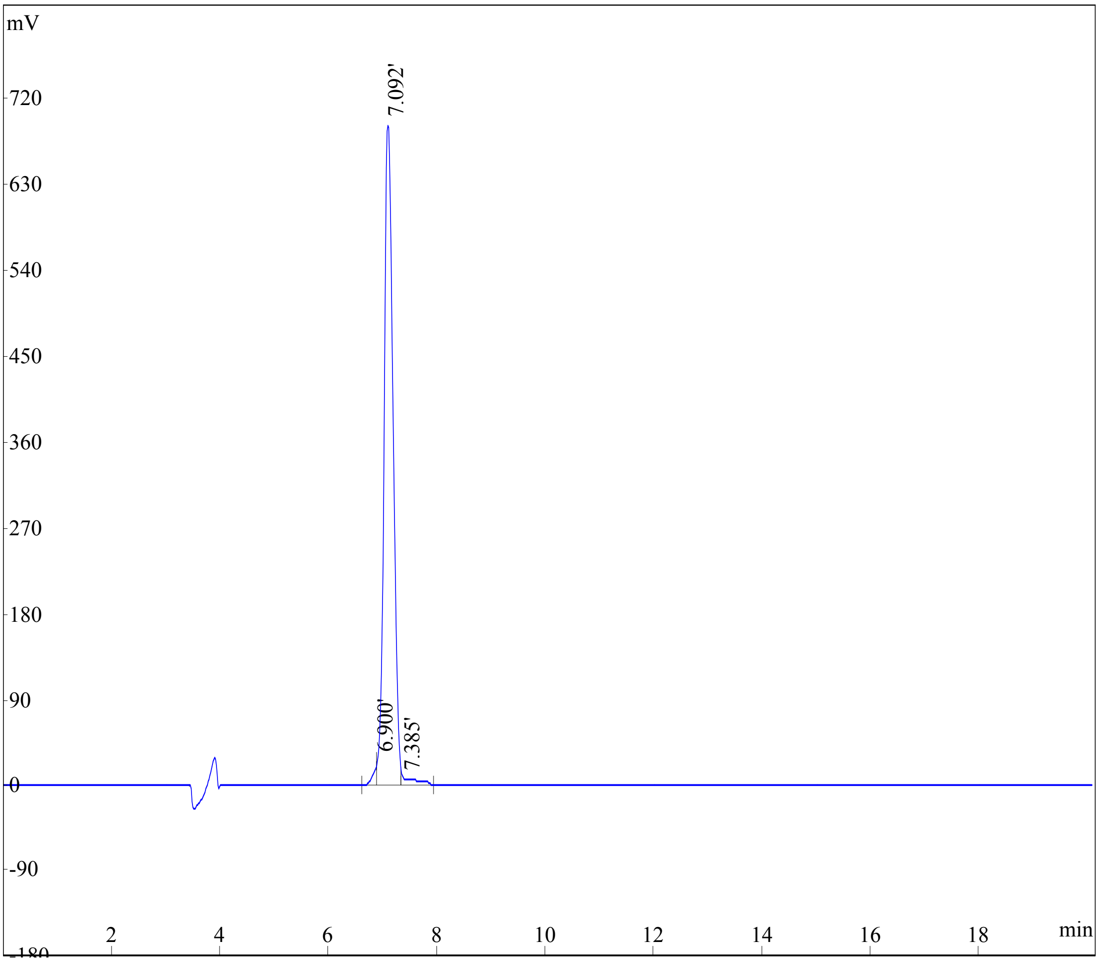

Rank	Time	Name	Conc.	Area
1	6.900		1.596	123594
2	7.092		96.2	7450498
3	7.385		2.205	170739
Total			100	7744831

MS Analysis Report

Ion Source: ESI Capillary(KV):±(2500~3500)

Desolvation(L/hr):800 Desolvation Temp:450℃

Cone(V): 15~30 Run Time: 1min

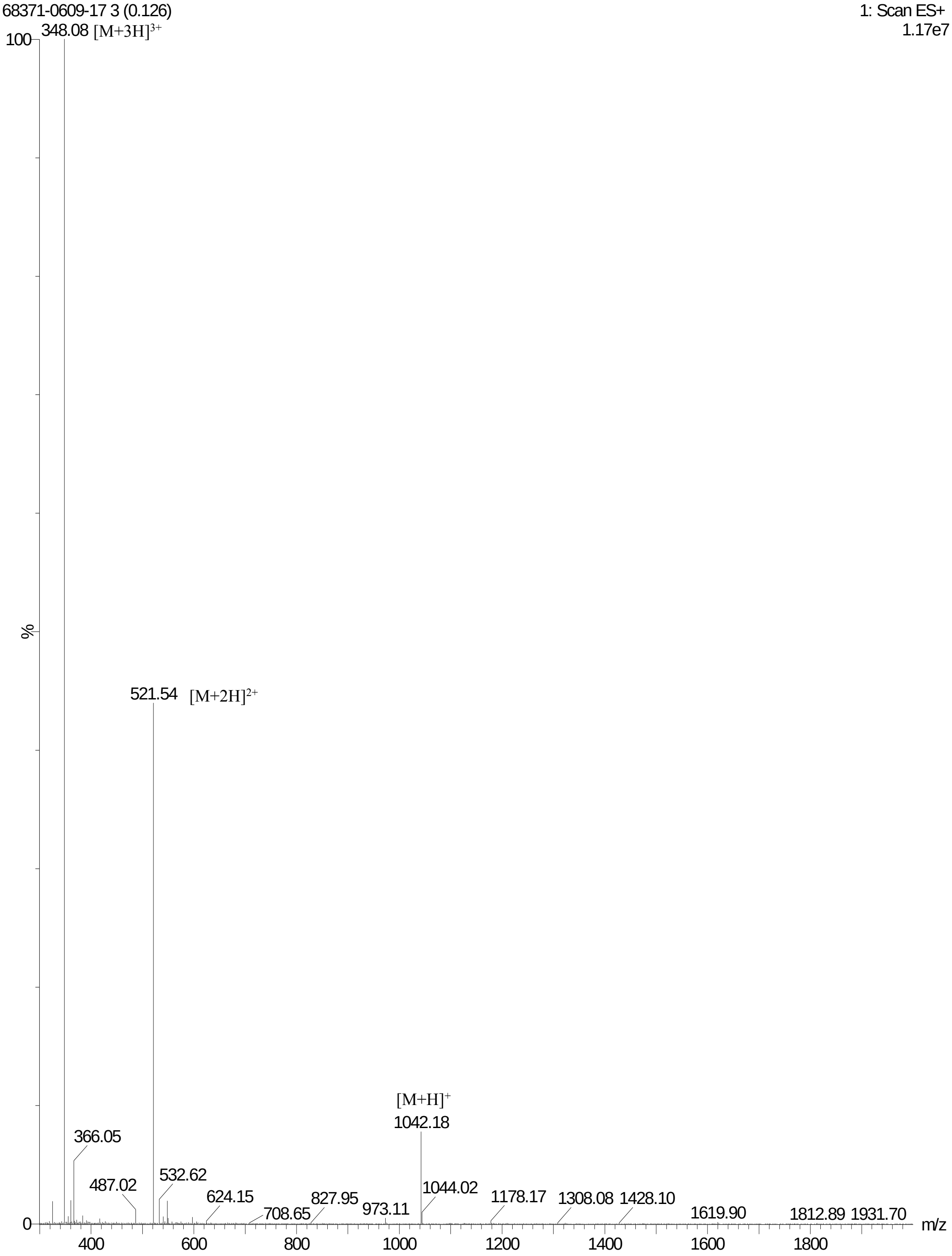

Page **Three** of **3**

Certificate of Analysis

Product Name: [Ala7]apTKRP-2b

Sequence: [p-]QPHLGFAGSR-NH2

Sequence(Three Letters Code): [p-]Gln-Pro-His-Leu-Gly-Phe-Ala-Gly-Ser-Arg-NH2

Purity: 97.88%

Molecular Weight: 1051.18

Solubility: 1mg/ml in 15%ACN/85%H_2O

Test	Specification	Result
**Purity:**	HPLC	Conforms
	(See attached RP-HPLC chromatogram)	
**MS Analysis:**	ESI-MS	Conforms
	(See attached MS spectrum)	
**Counter Ion:**	Trifluoroacetate	Conforms
**Appearance:**	Lyophilized powder or Crystallization	Conforms

Quality Assurance By: ______________ **Position**: Manager **Date**: 2022-06-17

Important: The peptides can be used for research only. Most of the peptides are lyophilized white or faint yellow powder while fluorescent modified ones have special colors. The state of peptides with strong hydrophilic properties may be crystalline or liquid which does not affect for use. Before experiment, please choose proper solvent for your experiment to dissolve peptides. If peptides cannot be dissolved under harsh conditions, we can carry out feasibility study. Storage conditions:-20ºC, seal, avoid light, dry.

Please test the sample within two weeks after receiving it.

注意：本品仅供科研，生产用途，不得直接用于人体。

HPLC Analysis Report

Measurement:	Peak Area	Run Time:	20min
Calculation Type:	Percent	Wavelength :	220nm
Flow Rate :	1.0ml/min	Inj.Vol:	10uL
Column:	Kromasil 100-5C18,4.6mmX250mm,5 micron	Column Temp:	25℃
Buffer A :	0.1%TFA in Acetonitrile	Buffer B:	0.1%TFA in water

Gradient(linear):

	A	B
0.0min	22%	78%
20min	47%	53%
20.1min	100%	0%

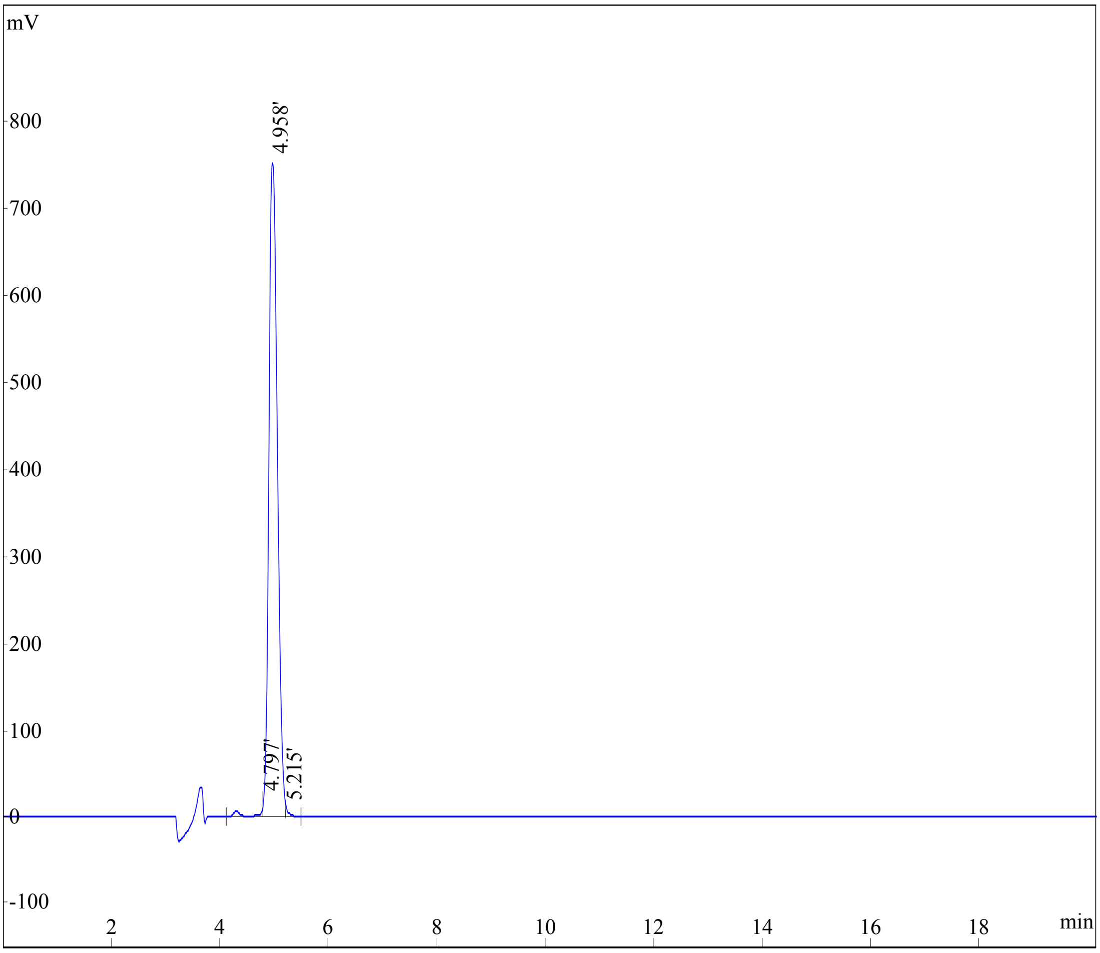

Rank	Time	Name	Conc.	Area
1	4.797		1.599	123914
2	4.958		97.88	7584111
3	5.215		0.5254	40713
Total			100	7748738

Page **Two** of **3**

MS Analysis Report

Ion Source: ESI　　Capillary(KV):±(2500~3500)

Desolvation(L/hr):800　　Desolvation Temp:450℃

Cone(V): 15~30　　Run Time: 1min

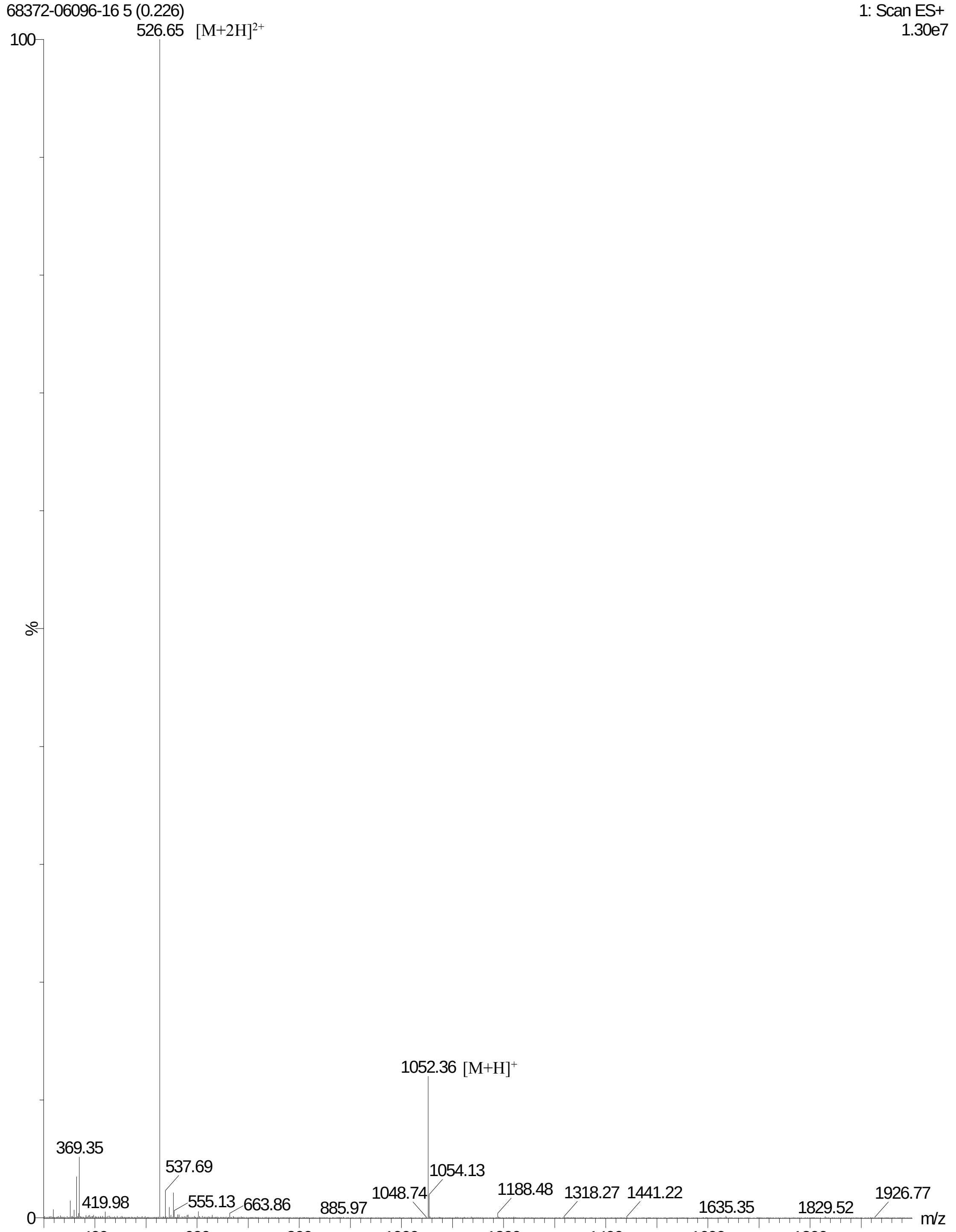

Certificate of Analysis

Product Name: [Ala8]apTKRP-2b

Sequence: [p-]QPHLGFHASR-NH2

Sequence(Three Letters Code): [p-]Gln-Pro-His-Leu-Gly-Phe-His-Ala-Ser-Arg-NH2

Purity: 96.58%

Molecular Weight: 1131.27

Solubility: 1mg/ml in 15%ACN/85%H_2O

**Test**	**Specification**	**Result**
**Purity:**	HPLC	Conforms
	(See attached RP-HPLC chromatogram)	
**MS Analysis:**	ESI-MS	Conforms
	(See attached MS spectrum)	
**Counter Ion:**	Trifluoroacetate	Conforms
**Appearance:**	Lyophilized powder or Crystallization	Conforms

Quality Assurance By: ____________ **Position**: Manager **Date**: 2022-06-17

Important: The peptides can be used for research only. Most of the peptides are lyophilized white or faint yellow powder while fluorescent modified ones have special colors. The state of peptides with strong hydrophilic properties may be crystalline or liquid which does not affect for use. Before experiment, please choose proper solvent for your experiment to dissolve peptides. If peptides cannot be dissolved under harsh conditions, we can carry out feasibility study. Storage conditions:-20ºC, seal, avoid light, dry.

Please test the sample within two weeks after receiving it.

注意：本品仅供科研，生产用途，不得直接用于人体。

HPLC Analysis Report

Measurement:	Peak Area	Run Time:	20min
Calculation Type:	Percent	Wavelength :	220nm
Flow Rate :	1.0ml/min	Inj.Vol:	10uL
Column:	Kromasil 100-5C18,4.6mmX250mm,5 micron	Column Temp:	25℃
Buffer A :	0.1%TFA in Acetonitrile	Buffer B:	0.1%TFA in water

Gradient(linear):		A	B
	0.0min	15%	85%
	20min	40%	60%
	20.1min	100%	0%

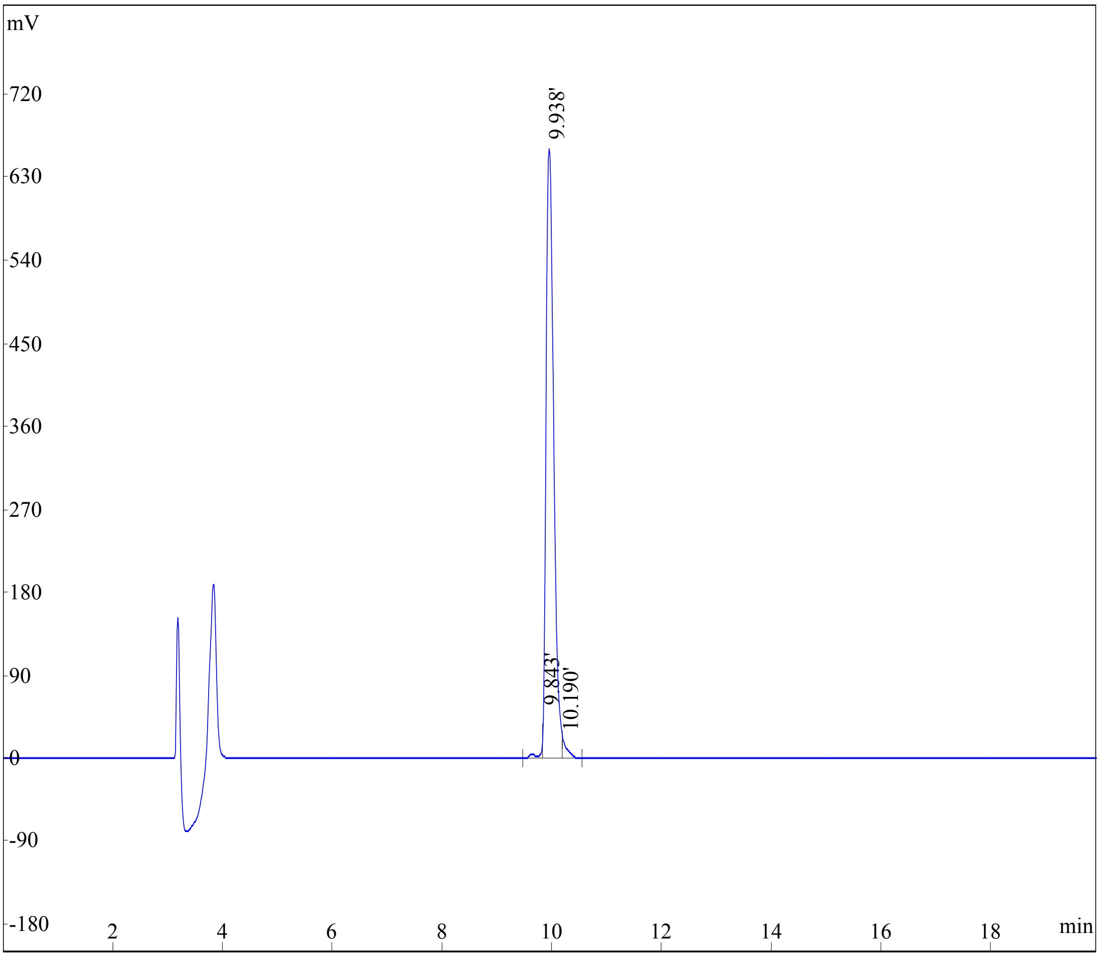

Rank	Time	Name	Conc.	Area
1	9.843		1.433	86458
2	9.938		96.58	5828605
3	10.190		1.982	119623
Total			100	6034686

MS Analysis Report

Ion Source: ESI Capillary(KV):±(2500~3500)
Desolvation(L/hr):800 Desolvation Temp:450℃
Cone(V): 15~30 Run Time: 1min

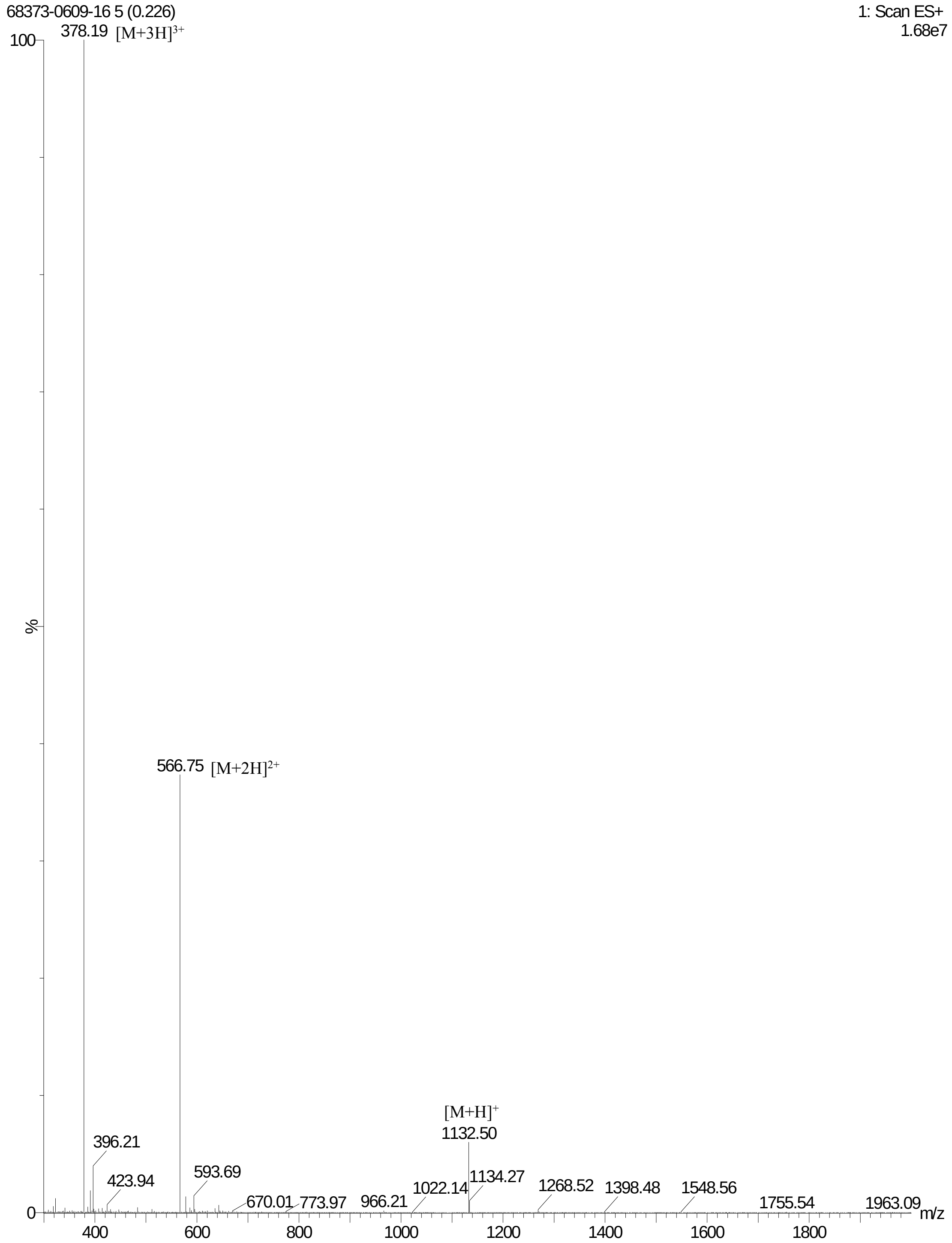

Certificate of Analysis

Product Name: [Ala^9]apTKRP-2b

Sequence: [p-]QPHLGFHGAR-NH2

Sequence(Three Letters Code): [p-]Gln-Pro-His-Leu-Gly-Phe-His-Gly-Ala-Arg-NH2

Purity: 96.66%

Molecular Weight: 1101.25

Solubility: 1mg/ml in 15%ACN/85%H_2O

Test	Specification	Result
**Purity:**	HPLC	Conforms
	(See attached RP-HPLC chromatogram)	
**MS Analysis:**	ESI-MS	Conforms
	(See attached MS spectrum)	
**Counter Ion:**	Trifluoroacetate	Conforms
**Appearance:**	Lyophilized powder or Crystallization	Conforms

Quality Assurance By: ____________ **Position**: Manager **Date**: 2022-06-17

Important: The peptides can be used for research only. Most of the peptides are lyophilized white or faint yellow powder while fluorescent modified ones have special colors. The state of peptides with strong hydrophilic properties may be crystalline or liquid which does not affect for use. Before experiment, please choose proper solvent for your experiment to dissolve peptides. If peptides cannot be dissolved under harsh conditions, we can carry out feasibility study. Storage conditions:-20ºC, seal, avoid light, dry.

Please test the sample within two weeks after receiving it.

注意：本品仅供科研，生产用途，不得直接用于人体。

HPLC Analysis Report

Measurement:	Peak Area	Run Time:	20min
Calculation Type:	Percent	Wavelength :	220nm
Flow Rate :	1.0ml/min	Inj.Vol:	10uL
Column:	Kromasil 100-5C18,4.6mmX250mm,5 micron	Column Temp:	25℃
Buffer A :	0.1%TFA in Acetonitrile	Buffer B:	0.1%TFA in water

Gradient(linear):		A	B
	0.0min	15%	85%
	20min	40%	60%
	20.1min	100%	0%

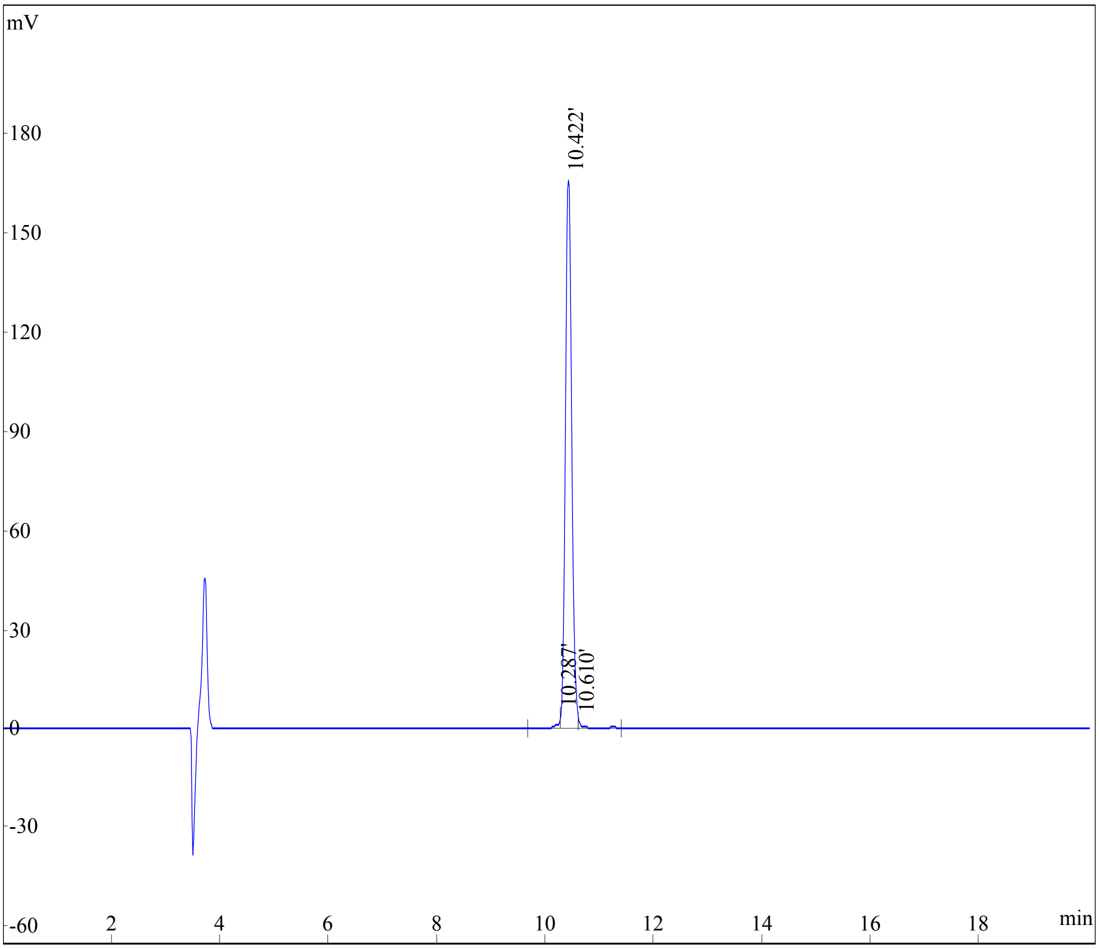

Rank	Time	Name	Conc.	Area
1	10.287		1.594	20144
2	10.422		96.66	1221933
3	10.610		1.742	22020
Total			100	1264097

MS Analysis Report

Ion Source: ESI Capillary(KV):±(2500~3500)

Desolvation(L/hr):800 Desolvation Temp:450℃

Cone(V): 15~30 Run Time: 1min

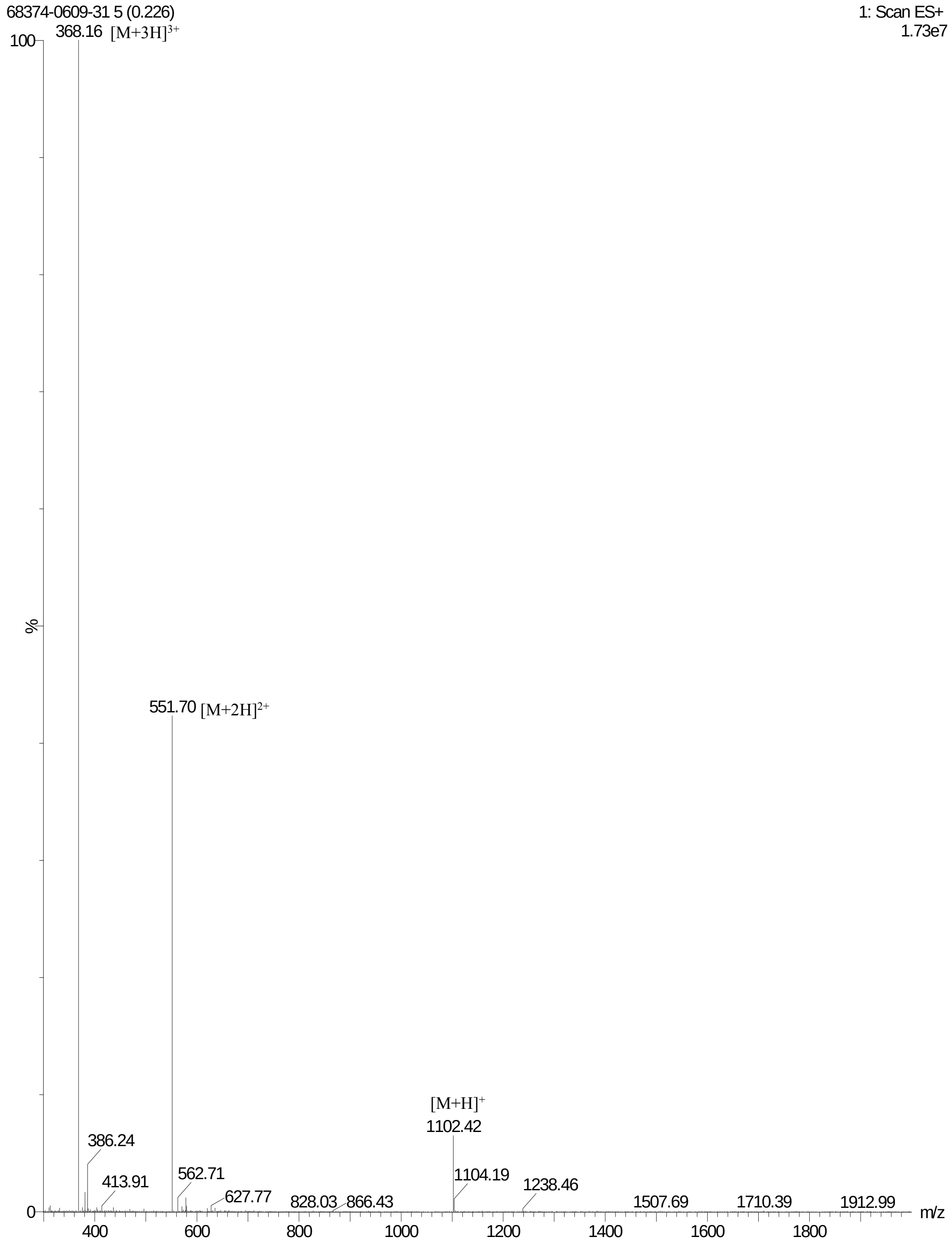

Certificate of Analysis

Product Name: [Ala^{10}]apTKRP-2b

Sequence: [p-]QPHLGFHGSA-NH2

Sequence(Three Letters Code): [p-]Gln-Pro-His-Leu-Gly-Phe-His-Gly-Ser-Ala-NH2

Purity: 98.92%

Molecular Weight: 1032.14

Solubility: 1mg/ml in 15%ACN/85%H_2O

**Test**	**Specification**	**Result**
**Purity:**	HPLC	Conforms
	(See attached RP-HPLC chromatogram)	
**MS Analysis:**	ESI-MS	Conforms
	(See attached MS spectrum)	
**Counter Ion:**	Trifluoroacetate	Conforms
**Appearance:**	Lyophilized powder or Crystallization	Conforms

Quality Assurance By: ____________ **Position**: Manager **Date**: 2022-06-17

Important: The peptides can be used for research only. Most of the peptides are lyophilized white or faint yellow powder while fluorescent modified ones have special colors. The state of peptides with strong hydrophilic properties may be crystalline or liquid which does not affect for use. Before experiment, please choose proper solvent for your experiment to dissolve peptides. If peptides cannot be dissolved under harsh conditions, we can carry out feasibility study. Storage conditions:-20ºC, seal, avoid light, dry.

Please test the sample within two weeks after receiving it.

注意：本品仅供科研，生产用途，不得直接用于人体。

HPLC Analysis Report

Measurement:	Peak Area	Run Time:	20min
Calculation Type:	Percent	Wavelength :	220nm
Flow Rate :	1.0ml/min	Inj.Vol:	10uL
Column:	Kromasil 100-5C18,4.6mmX250mm,5 micron	Column Temp:	25℃
Buffer A :	0.1%TFA in Acetonitrile	Buffer B:	0.1%TFA in water

Gradient(linear):		A	B
	0.0min	17%	83%
	20min	42%	58%
	20.1min	100%	0%

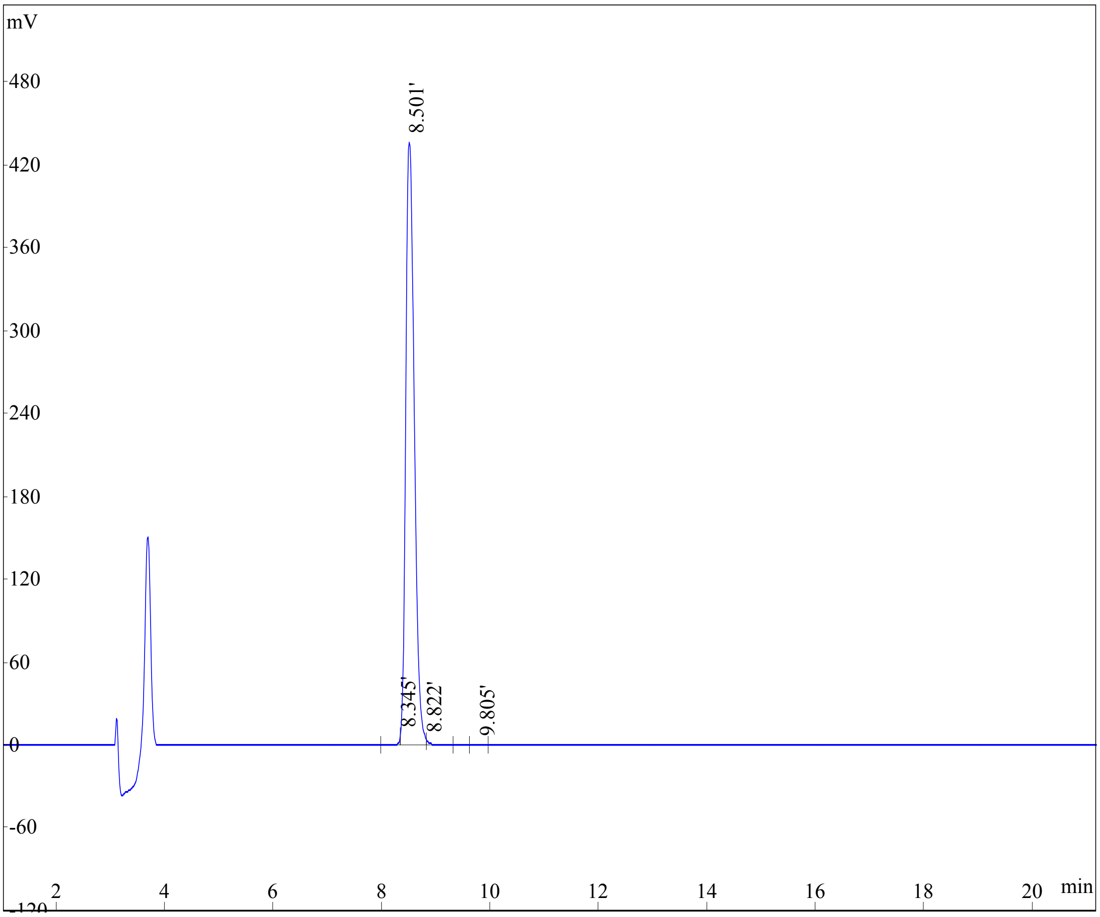

Rank	Time	Name	Conc.	Area
1	8.345		0.4896	21921
2	8.501		98.92	4429546
3	8.822		0.47	21042
4	9.805		0.116	5194
Total			100	4477703

MS Analysis Report

Ion Source: ESI　　　Capillary(KV):±(2500~3500)

Desolvation(L/hr):800　　　Desolvation Temp:450℃

Cone(V): 15~30　　　Run Time: 1min

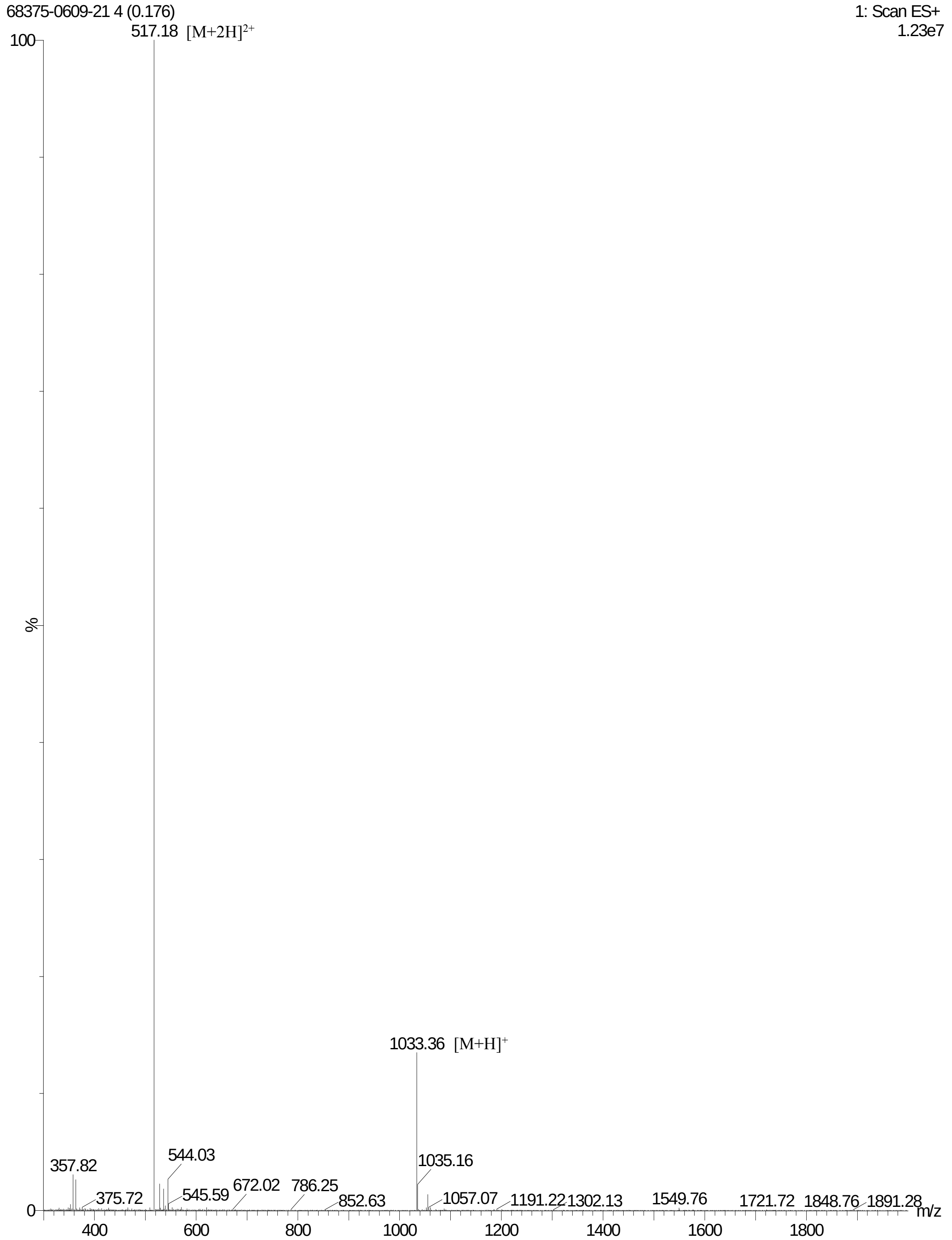

apTKRP-2b-OH

安徽省国平药业有限公司

CERTIFICATE OF ANALYSIS

**Order ID**	GP120687-2
**Name**	N/A
**Lot No.**	GP120687-2-0614
**Sequence**	[p-]QPHLGFHGSR
**Dissolution condition**	100%H2O
**Length**	10AA
**Modification**	N/A
**Molecular Weight (MW)**	1118.32
**Storage**	-20°C

**Test Items**	**Specifications**	**Results**
**MW by MS**	1118.25	Conforms
**Purity by HPLC**	>95%	95.843%
**Peptide Content**	N/A	N/A
**Moisture content**	N/A	N/A
**Acetic acid content**	N/A	N/A
**Appearance**	White to off-white lyophilized powder	Conforms
**Quantity**	10mg	2.0mg*5

Certified by: LiuHui Date 06/21/2022

Quality Assurance Department

Note: this product is intended for research use only; not for diagnostic or human use.

Guoping Pharmaceutical Co., LTD

地址:合肥市经开区桃花工业园拓展区工投立恒工业广场A2西F1,电话:0551-62841987 传真:0551-62841765 www.guopingyaoye.com

Sample Information

Order ID :GP120687-2
Name :N/A
Sequence :[p-]QPHLGFHGSR
Lot.No :GP120687-2-0614
Pump A :0.1%Trifluoroacetic in 100% water
Pump B :0.1%Trifluoroacetic in 100% acetonitrile
Total Flow :1ml/min
Wavelength :220nm
Analytical column type :SHIMADZU Inertsil ODS-SP(4.6*250mm*5um)
Dissolution method :100%H2O
Inj. Volume :8 uL

Time	Module	Action	Value
0.01	Pumps	B.Conc	10
20.00	Pumps	B.Conc	50
23.00	Pumps	B.Conc	100
38.00	Pumps	B.Conc	100
40.00	Pumps	B.Conc	10
50.00	Controller	Stop	

Chromatogram

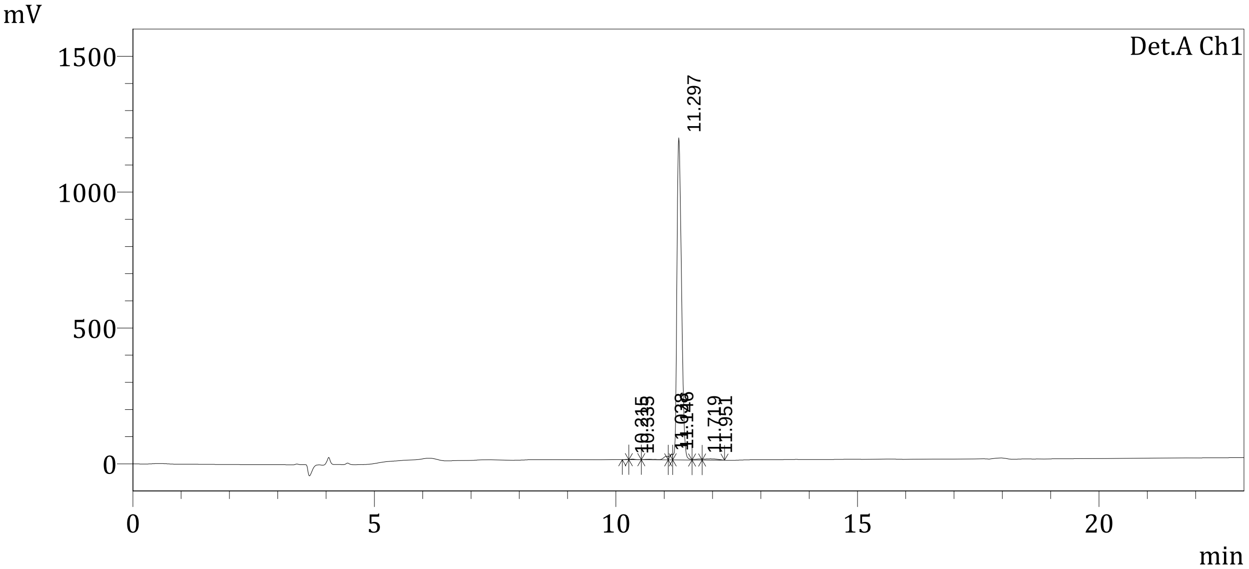

1 Det.A Ch1/220nm

PeakTable

Detector A Ch1 220nm

Peak#	Ret. Time	Area	Height	Area %	Height %
1	10.215	5984	1171	0.073	0.095
2	10.335	14515	2426	0.176	0.197
3	11.038	98796	13118	1.201	1.066
4	11.146	93122	19607	1.132	1.593
5	11.297	7884852	1185600	95.843	96.336
6	11.719	38881	4106	0.473	0.334
7	11.951	90687	4659	1.102	0.379
Total		8226837	1230687	100.000	100.000

MS Spectrum Graph

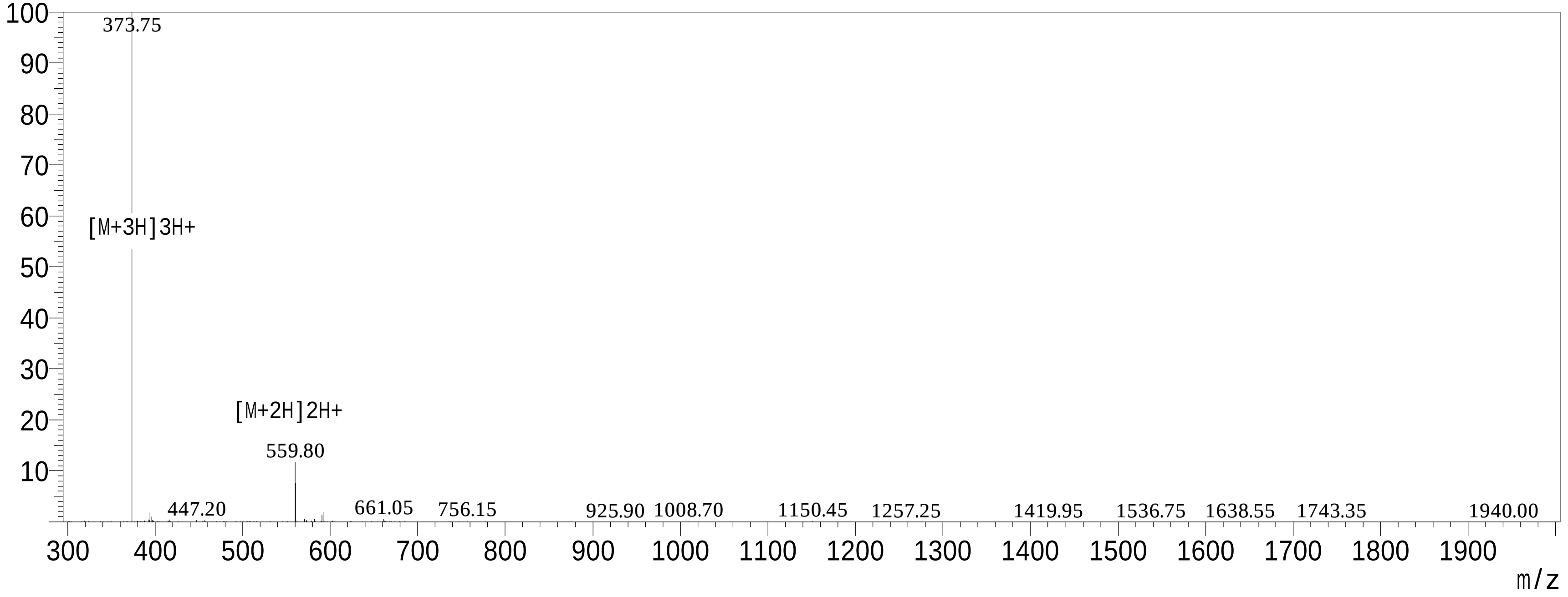

Sample Information

Dissolution method	:5%HAC+8%ACN+87%H2O	Interface	:ESI	Prerod Bias	:+1.5kv
Modified Date	:2022/06/21	Nebulizing Gas Flow	:1.50L/min	Detector	:-0.2kv
Injection Volume	:1ul	CDL Temp	:250C	T.Flow	:0.2ml/min
Heat Block Temp	:200	CDL Volt	:0v	B.conc	:50%H2O/50%MEOH

Order ID :GP120687-2
Name :N/A
Sequence :[p-]QPHLGFHGSR
Lot.No :GP120687-2-0614
Theoretical :1118.23
Observed :1118.25
