# Supplementary material for: Two C-terminal isoforms of Aplysia tachykinin–related peptide receptors exhibit phosphorylation-dependent and phosphorylation-independent desensitization mechanisms
Source: J Biol Chem. 2024 Jul 11;300(8):107556. doi: 10.1016/j.jbc.2024.107556 (PMC11365428; doi:10.1016/j.jbc.2024.107556)
Supplement: Supporting information [file mmc12.docx]

**Supporting Information**

**Two C-terminal isoforms of *Aplysia* tachykinin-related peptide receptors exhibit phosphorylation-dependent and independent desensitization mechanisms**

Rui-ting Mao^1#^, Shi-qi Guo^1#^, Guo Zhang^1*#^, Ya-dong Li^1#^, Ju-ping Xu^1^, Hui-ying Wang^1^, Ping Fu^1^, Cui-ping Liu^1^, Shao-qian Wu^1^, Ping Chen^1^, Yu-shuo Mei^1^, Qing-chun Jin^1^, Cheng-yi Liu^1^, Yan-chu-fei Zhang^1^, Xue-ying Ding^1^, Wei-jia Liu^1^, Elena V Romanova^2^, Hai-bo Zhou^3,4*^, Elizabeth C Cropper^5^, James W. Checco^6,7^, Jonathan V Sweedler^2^, Jian Jing^1,4,5*^

^1^ Department of Neurology and Medical Psychology, Nanjing Drum Tower Hospital, State Key Laboratory of Pharmaceutical Biotechnology, Institute for Brain Sciences, Chinese Academy of Medical Sciences Research Unit of Extracellular RNA, Jiangsu Engineering Research Center for MicroRNA Biology and Biotechnology, Advanced Institute for Life Sciences, Chemistry and Biomedicine Innovation Center, School of Life Sciences, Nanjing University, Nanjing, Jiangsu 210023, China

^2^Department of Chemistry and the Beckman Institute for Advanced Science and Technology, University of Illinois at Urbana-Champaign, Urbana 61801, Illinois, USA

^3^School of Electronic Science and Engineering, Nanjing University, Nanjing, Jiangsu 210023, China

^4^Peng Cheng Laboratory, Shenzhen 518000, China

^5^Department of Neuroscience and Friedman Brain Institute, Icahn School of Medicine at Mount Sinai, New York, NY 10029, USA

^6^Department of Chemistry, University of Nebraska-Lincoln, Lincoln, NE 68588, USA

^7^The Nebraska Center for Integrated Biomolecular Communication (NCIBC), University of Nebraska-Lincoln, Lincoln, NE 68588, USA

There are Supporting Results in this document. Additionally, there are a total of 21 supporting figures (Figs. S1-S21) and 10 supporting tables. Figs. S1-S20 are included in this document, whereas Fig. S21 is included as a separate PDF document with its legend provided below. The supporting tables are included as 10 separate Excel files (Table S1-S10.xlsx), whereas the legends for the tables are provided below.

Figure S21. Peptides synthesis information from commercial companies.

Table S1. List of primer sequences

Table S2. List of tachykinin (-related) peptides from different species

Table S3. List of peptides detected by mass spectrometry in the CNS of *Aplysia*

Table S4. List of predicted *Aplysia* tachykinin receptors from NCBI and AplysiaTools

Table S5. Blast with putative TKRP receptors in different animal models

Table S6. List of TK/TKRP receptors from different species used to generate Figure 5 (NCBI information)

Table S7. List of predicted *Aplysia* GRKs from NCBI and AplysiaTools

Table S8. List of GRKs from different species used to generate the phylogenetic tree

Table S9. List of predicted *Aplysia* arrestins from NCBI and AplysiaTools

Table S10. List of predicted and identified arrestins used to generate the phylogenetic tree

Supporting Results

Bioinformatic analysis of putative GRKs and arrestins in Aplysia

Desensitization of GPCRs can be mediated through phosphorylation or internalization, mainly involving G protein-coupled receptor kinases (GRKs) and arrestins (1-3), respectively. Given the absence of identified GRKs in mollusks, we utilized sequences of two *Drosophila* GRKs (M80493.1, M80494.1) (4) to conduct a BLAST search in the NCBI GenBank. This search yielded five *Aplysia* sequences (XM_013090689.1, XM_005103634.3, XM_005103633.3, XM_035971132.1, XM_005103632.3). The sequence XM_013090689.1 appeared incomplete in NCBI mRNA sequence; however, a complete sequence was found in the AplysiaTools database, alongside the other four sequences (Table S7.xlsx). Upon analysis, XM_013090689.1 was designated GRK 1, while the others, varying only at the C-terminus and likely products of alternative splicing, were named GRK 2a, 2b, 2c, and 2d (Figs. S12-S13, Table S7). A phylogenetic tree positioned the *Aplysia* GRK 1 alongside *Drosophila* GRK 1, and GRK 2a-d with *Drosophila* GRK 2 (Fig. S14, Table S8). Tissue expression profiles from NCBI’s RNA-seq database (GEO accession: GSE79231) (5) revealed the presence of these GRKs in the CNS and various peripheral tissues, as depicted in the expression level bar graphs (Fig. S15, except GRK 2c, which is unavailable in the database).

For arrestins, in the mollusk *Argopecten irradians*, two arrestins have been identified (HQ695998.1 and HQ695999.1) (6). Utilizing these sequences, a BLAST search identified four *Aplysia* sequences in NCBI and one in AplysiaTools (Table S9.xlsx). Notably, the proteins encoded by XM_013090298.1 and asmbl_21374 differ in the initial 23 amino acids at the N-terminus and were designated as arrestin 1a and 1b, respectively, with the remaining termed arrestin 2-4 (Figs. S16-S17, Table S9). A phylogenetic tree analysis showed that the *Aplysia* arrestin 1a and 1b cluster with the *Argopecten* arrestins, while other *Aplysia* arrestins align with those from *Danio* and *Mus* (Fig. S18, Table S10). Additionally, expression profiles (5) showed that the *Aplysia* arrestin 1a (the other arrestins are unavailable in the database) is present in the CNS and several peripheral tissues, as illustrated by the TPM bar graphs (Fig. 19).

This comprehensive bioinformatic and expression analysis strongly support the presence of both GRKs and arrestins in *Aplysia*. Future work is needed to validate their presence and demonstrate their functional roles.

Supporting references

1. Smith, J. S., Lefkowitz, R. J., and Rajagopal, S. (2018) Biased signalling: from simple switches to allosteric microprocessors. *Nat Rev Drug Discov* **17**, 243-260

2. Gurevich, V. V., and Gurevich, E. V. (2019) GPCR signaling regulation: The role of GRKs and Arrestins. *Frontiers in Pharmacology* **10**, 125

3. Patwardhan, A., Cheng, N., and Trejo, J. (2021) Post-translational modifications of G Protein-coupled receptors control cellular signaling dynamics in space and time. *Pharmacol Rev* **73**, 120-151

4. Cassill, J. A., Whitney, M., Joazeiro, C. A., Becker, A., and Zuker, C. S. (1991) Isolation of *Drosophila* genes encoding G protein-coupled receptor kinases. *Proc Natl Acad Sci U S A* **88**, 11067-11070

5. Gyori, J., Kohn, A. B., Romanova, D. Y., and Moroz, L. L. (2021) ATP signaling in the integrative neural center of *Aplysia californica*. *Sci Rep* **11**, 5478

6. Gomez Mdel, P., Espinosa, L., Ramirez, N., and Nasi, E. (2011) Arrestin in ciliary invertebrate photoreceptors: molecular identification and functional analysis in vivo. *J Neurosci* **31**, 1811-1819

Supporting Figures


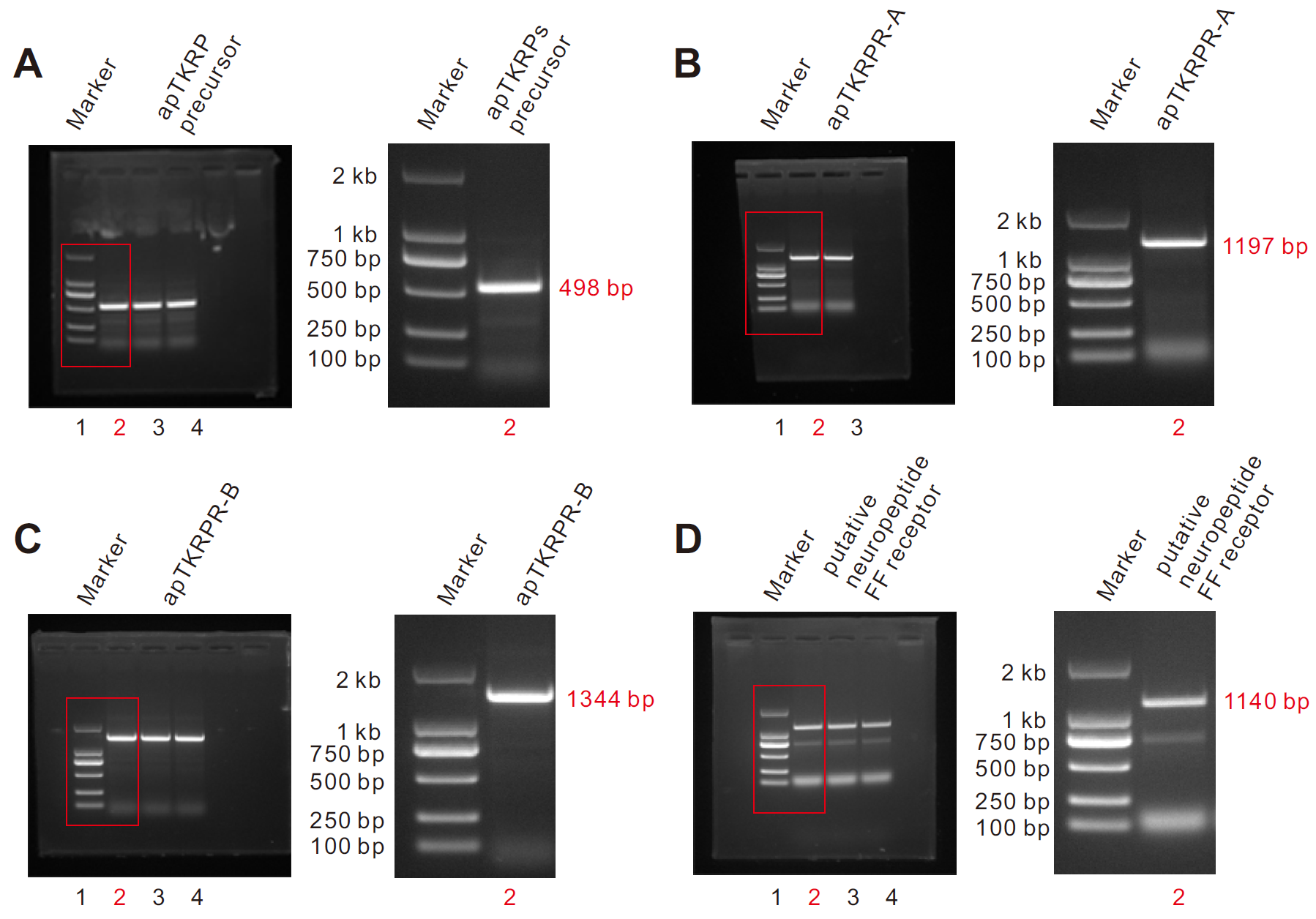


**Figure S1. Complete gels for PCR experiments.** *A*, apTKRP precursor. *B*, apTKRPR-A. *C*, apTKRPR-B. *D*, putative neuropeptide FF receptor. Left panels: complete gels with cropped areas indicated by the red boxes; Right panels, corresponding cropped gels shown in Figure 1*D* and Figure 6*A - C*. Lane 1 was DNA marker, and the other lanes were all PCR products of apTKRPR precursor or putative receptors.


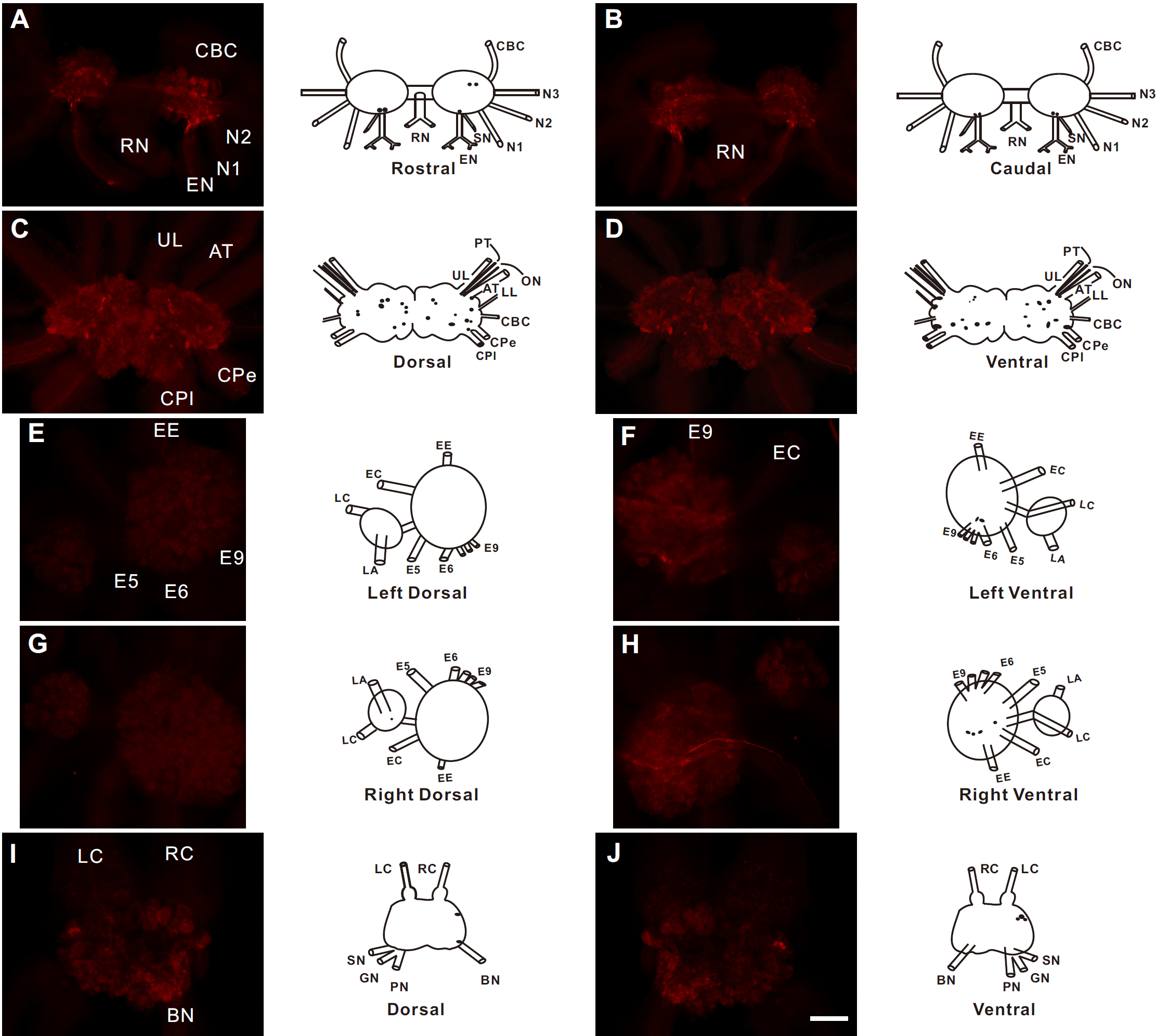


**Figure S2. Localization of apTKRPs in the *Aplysia* CNS using immunohistochemistry.** *A* and *B*, Rostral (*A*) and caudal (*B*) buccal ganglia. *C* and *D*, Dorsal (*C*) and ventral (*D*) cerebral ganglia. *E-H*, Left dorsal (*E*), left ventral (*F*), right dorsal (*G*), and right ventral (*H*) pedal-pleural ganglia. *I* and *J*, Dorsal (*I*) and ventral (*J*) abdominal ganglia. Immunohistochemistry reveals that the majority of *apTKRP*-positive neurons are distributed in the cerebral ganglia. Scale bar, 500 μm. Buccal abbreviations are as follows. N1, nerve 1; N2, nerve 2; N3, nerve 3; SN, salivary nerve; EN, esophageal nerve; RN, radula nerve. Cerebral abbreviations are as follows. UL, upper labial nerve; PT, posterior tentacular nerve; ON, optic nerve; AT, anterior tentacular nerve; LL, lower labial nerve; CBC, cerebrobuccal connective; CPe, cerebropedal connective; CPl, cerebropleural connective. Pedal-pleural abbreviations are as follows. EE, pedal commissure; EC, cerebropedal connective; LC, cerebropleural connective; LA, pleuroabdominal connective; E5, posterior tegumentary nerve (P5); E6, anterior parapodial nerve (P6); E9, posterior pedal nerve (P9). Abdominal abbreviations are as follows. LC, left pleuroabdominal connective; RC, right pleuroabdominal connective; BN, branchial nerve; PN, pericardial nerve; GN, genital nerve; SN, siphon nerve.


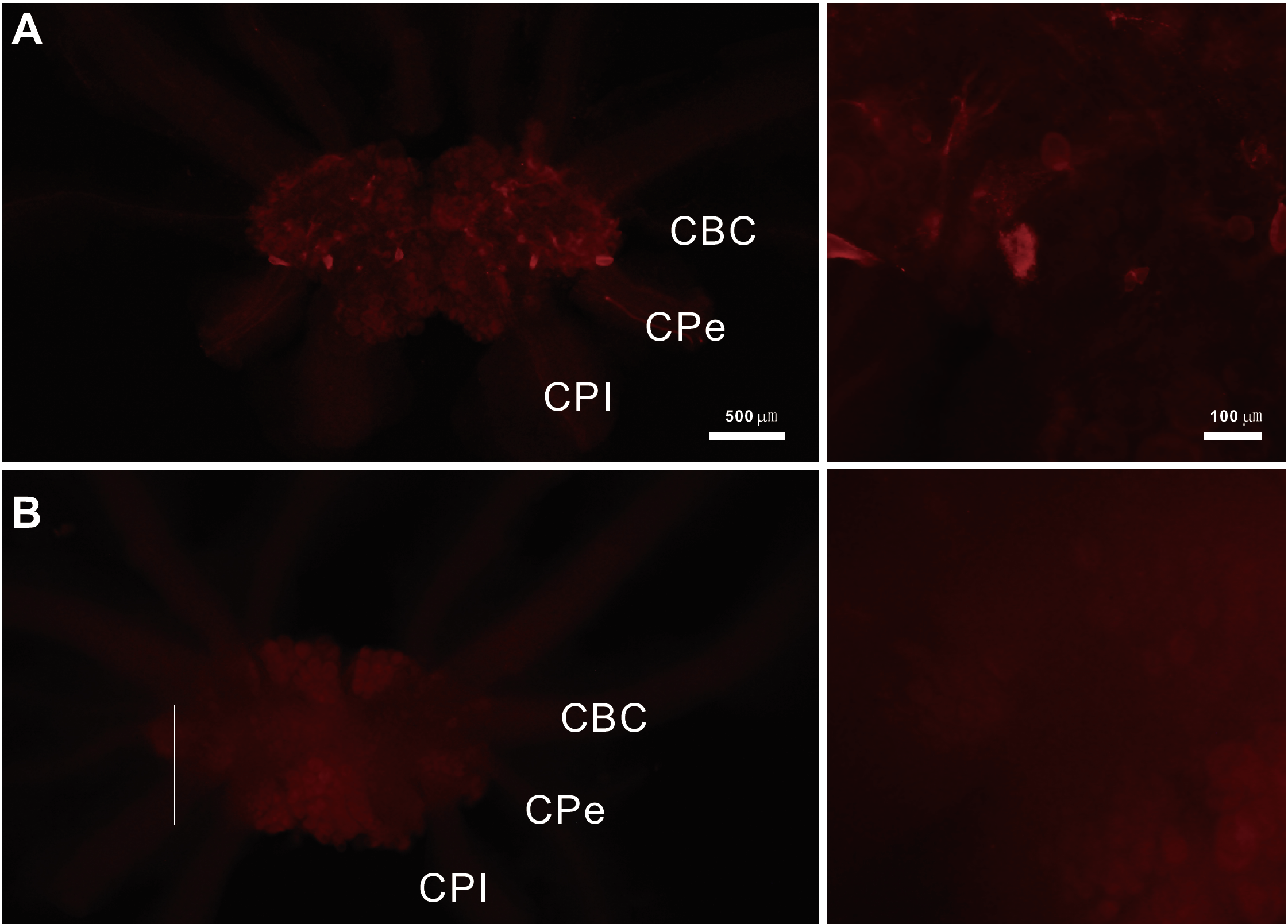


**Figure S3. Comparison of a control experiment and preabsorption experiment with two cerebral ganglia in immunohistochemistry.** *A*. A control experiment showing positively stained neurons of a cerebral ganglion (ventral). Note that the control experiment for this panel was derived from the same experiment as that shown in Fig. S2*D*, thus, this panel is a reuse of Fig. S2*D*. *B*. A preabsorption experiment showing no positively stained neurons of a different cerebral ganglion (ventral). Right panels are enlarged images showing part of the left images marked in white squares.

**A**
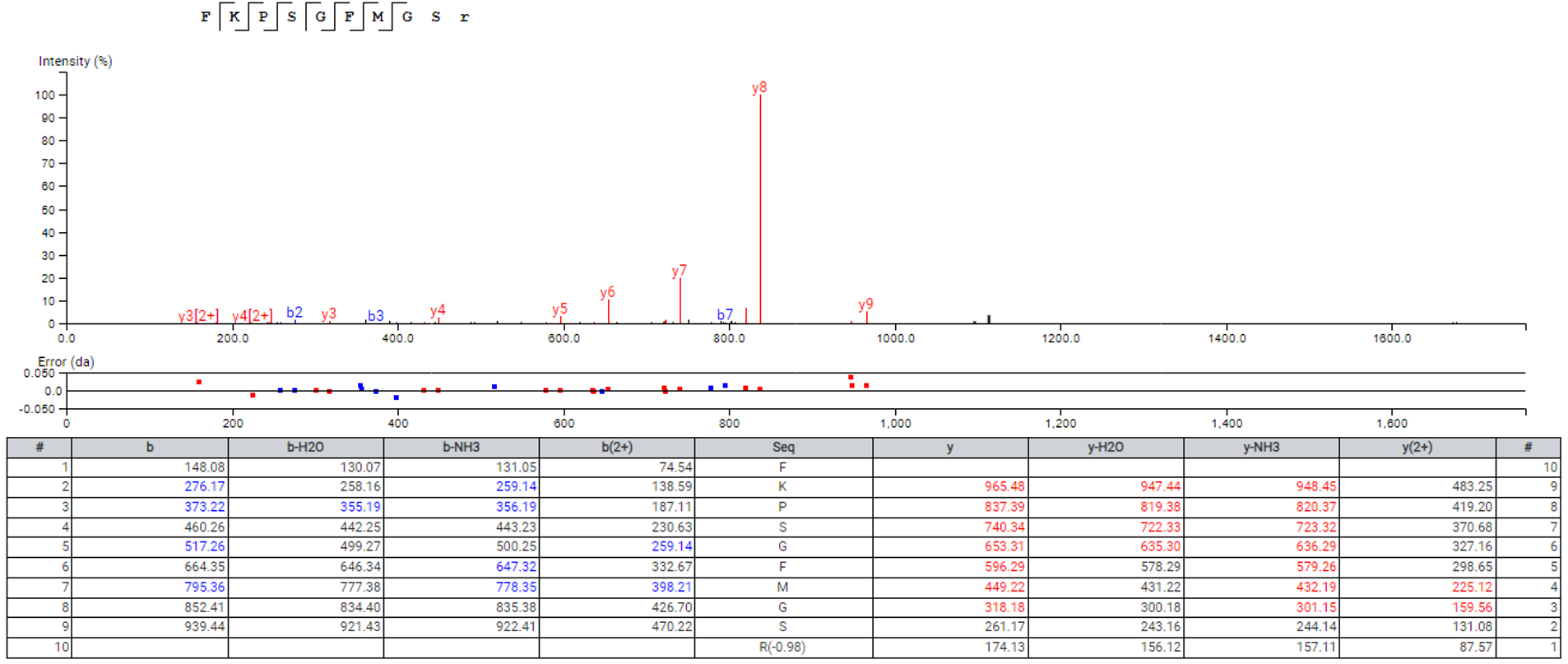


**B**
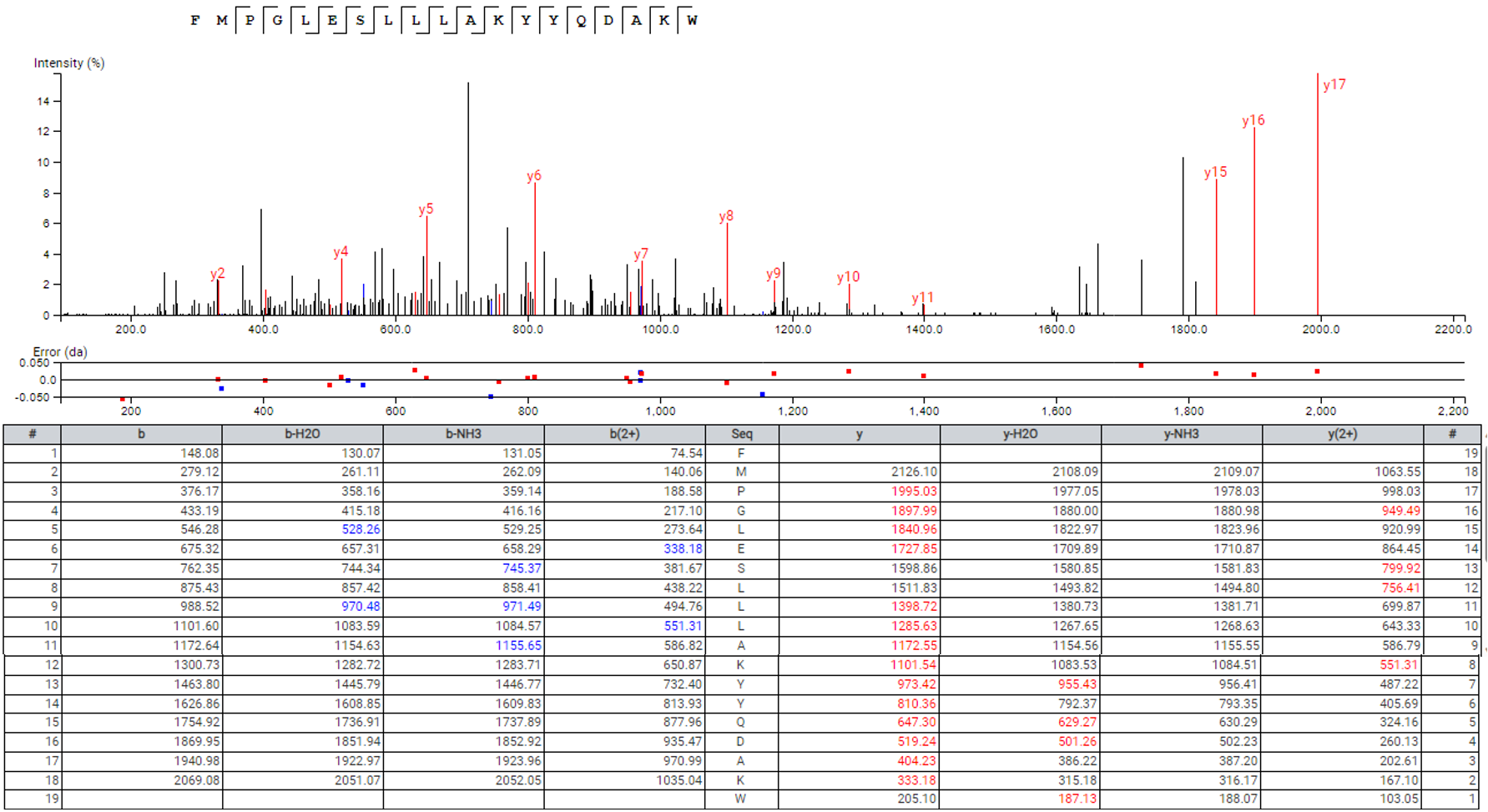


**C**
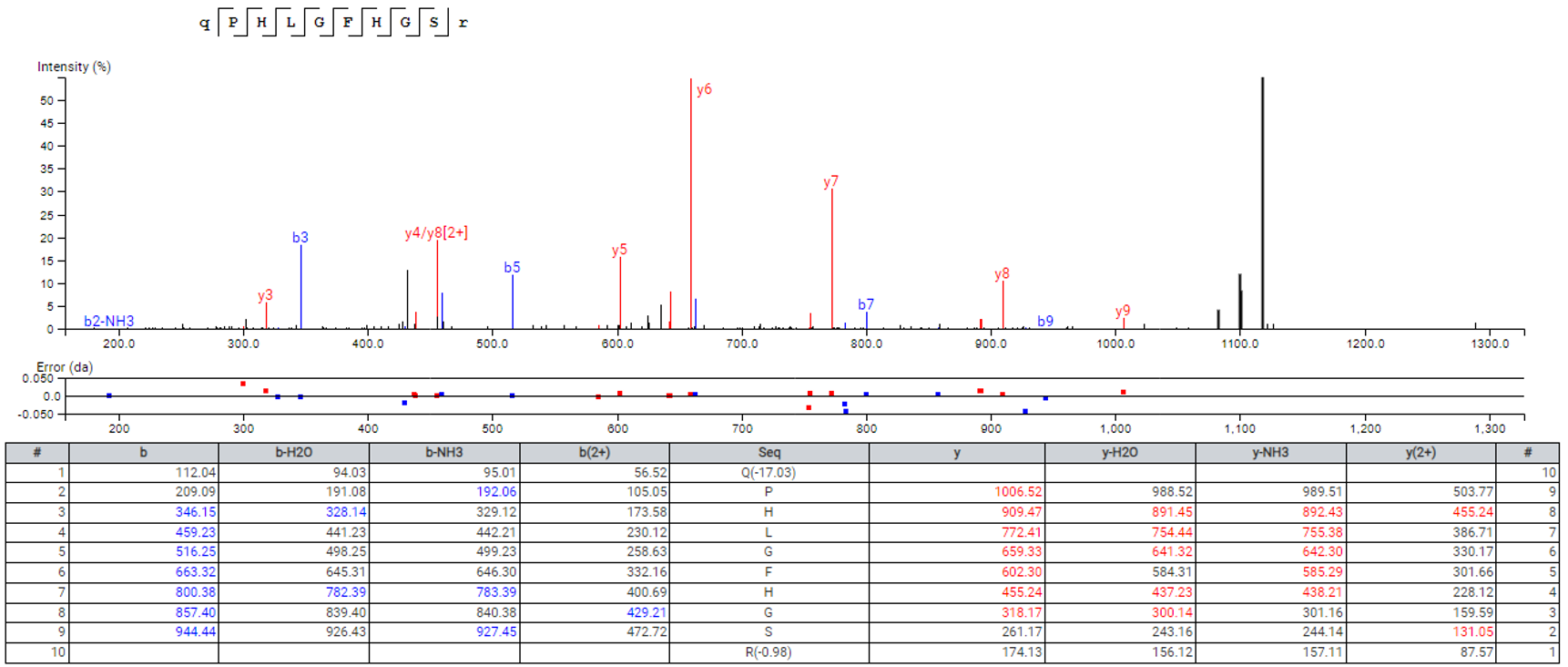


**D**
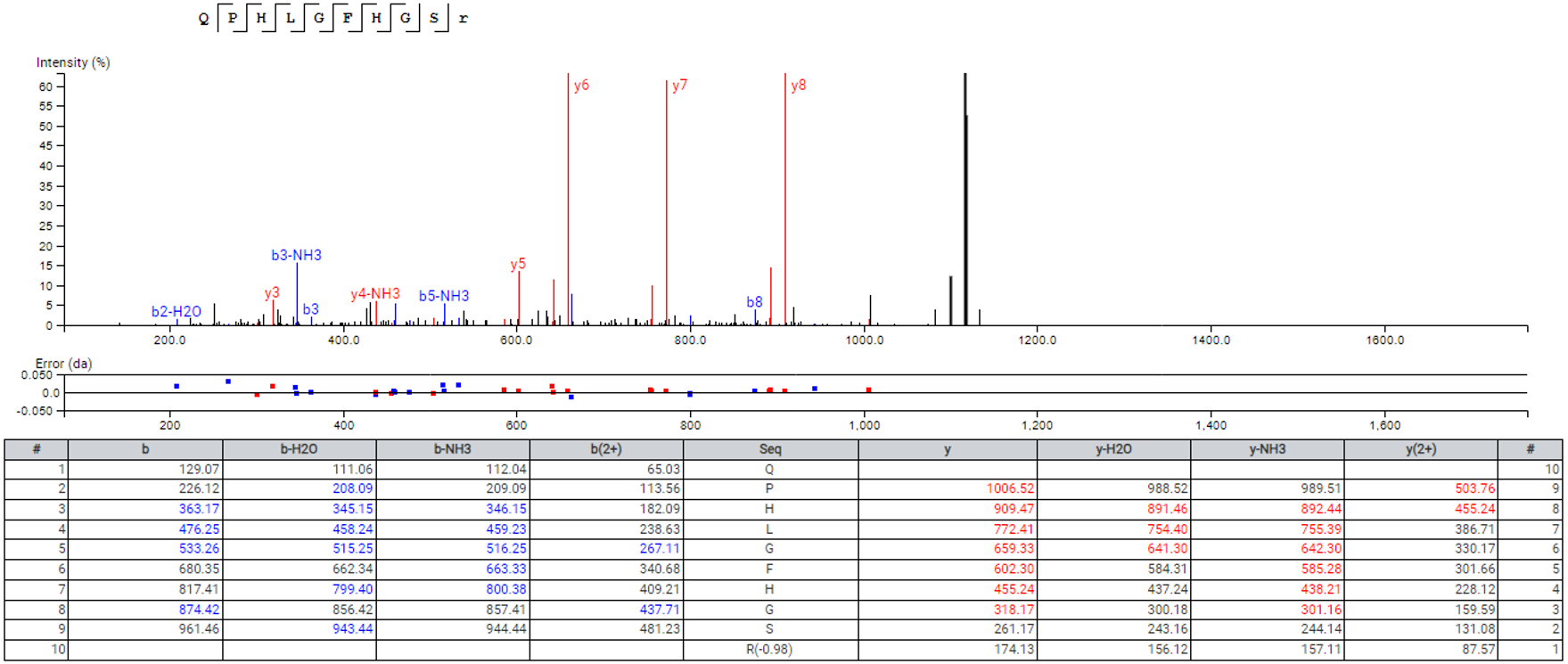


**Figure S4. Representative mass spectra for some of the predicted peptides.** *A*, apTKRP-1: FKPSGFMGSr (FKPSGFMGSR(-0.98)). *B*, apTKRPG-DP: FMPGLESLLLAKYYQDAKW. *C*, apT2KRP-2b: qPHLGFHGSr (Q(-17.03)PHLGFHGSR(-0.98)). *D*, apTKRP-2a: QPHLGFHGSr (QPHLGFHGSR(-0.98)). q or Q(-17.03) is equivalent to [p-]Q; r or R(-0.98) is equivalent to R-NH_2_. Tables below the mass spectra are corresponding ion tables.


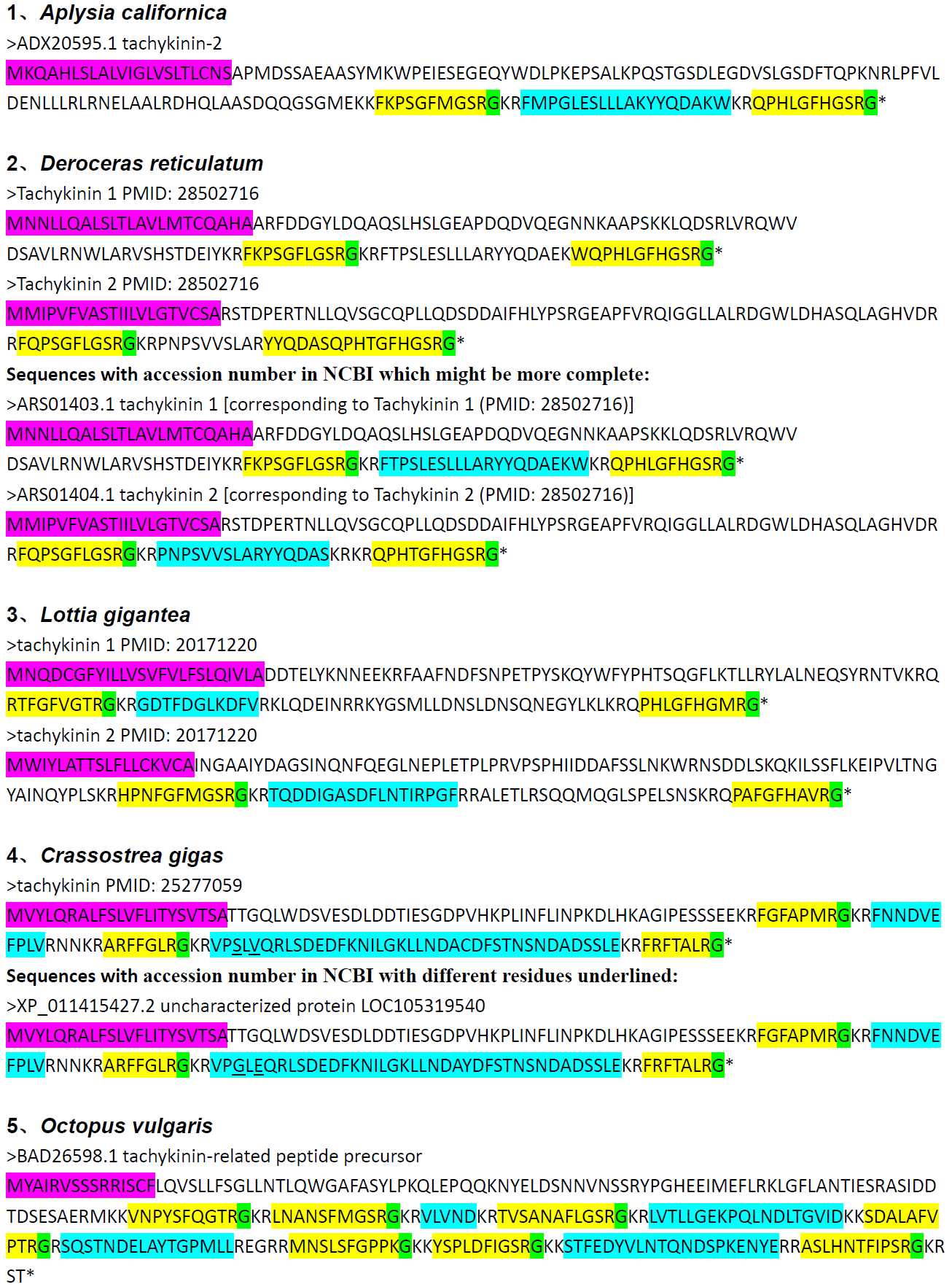


**Figure S5. Prediction of putative peptides from TKRP precursors of five molluscan species using Neuropred.** The predicted signal peptides are highlighted in purple. Glycine residues predicted to be converted in C-terminal amide are shown in green, while likely biologically active neuropeptides are highlighted in yellow. apTKRPG-DP and similar putative neuropeptides are highlighted in blue. The stop codon at the end of the coding sequence is denoted by an asterisk. For the five sequences without NCBI accession numbers, the PMIDs of the articles that predicted them are shown. Similar sequences with NCBI accession numbers for *Deroceras reticulatum* and *Crassostrea gigas* are also shown, which differ from the corresponding sequences in the articles by about 2-4 residues. In addition, in *Deroceras reticulatum*, the predicted splicing sites may be different from their corresponding sequences.


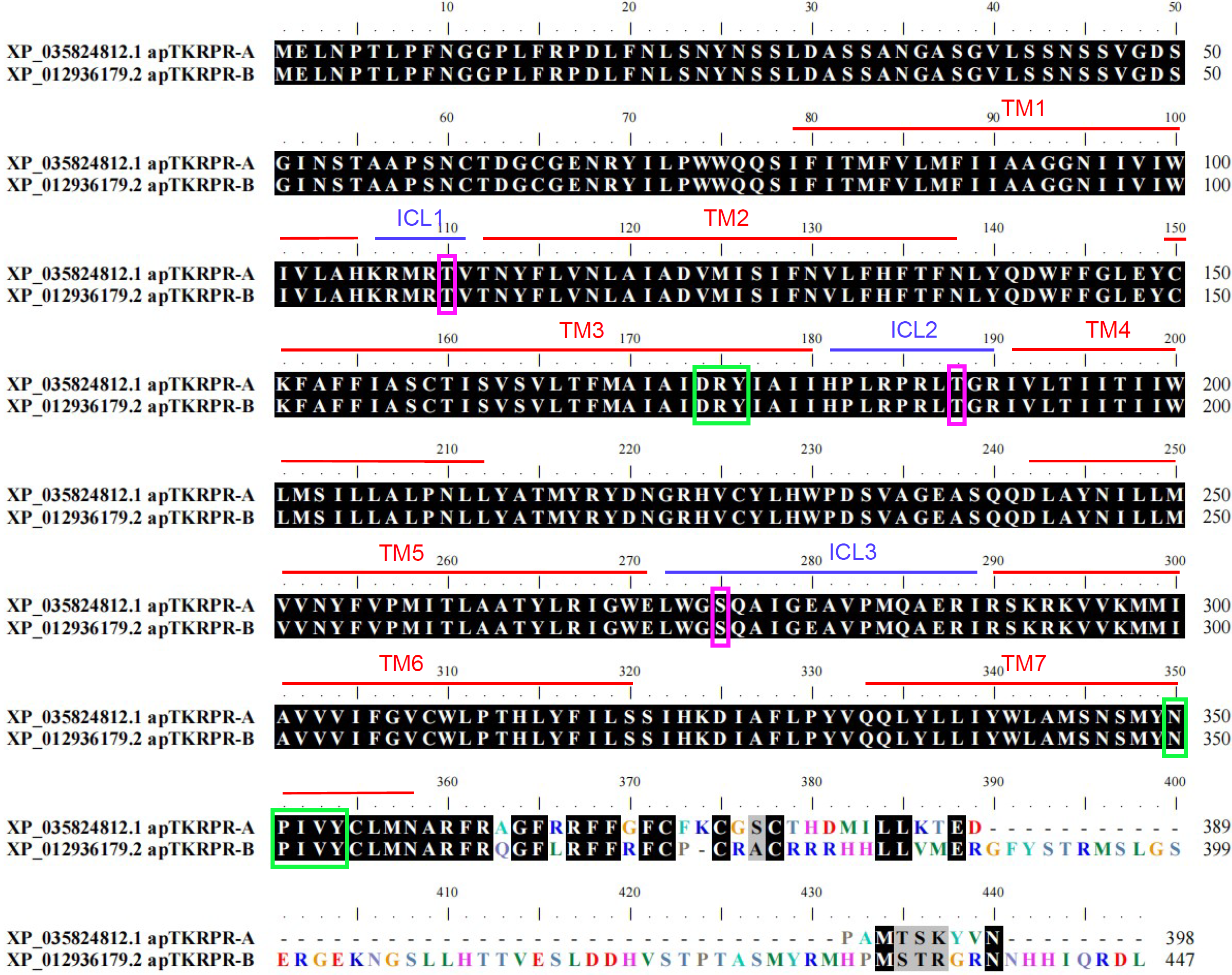


**Figure S6. Comparison of the apTKRPR-A with apTKRPR-B using BioEdit.** The 7 predicted transmembrane domains (TMs) as well as the three intracellular loops (ICLs) are indicated. The conserved motif DRY and NPXXY are marked by green boxes, whereas ser/thr residues in the three ICLs are marked by purple boxes.

**A**


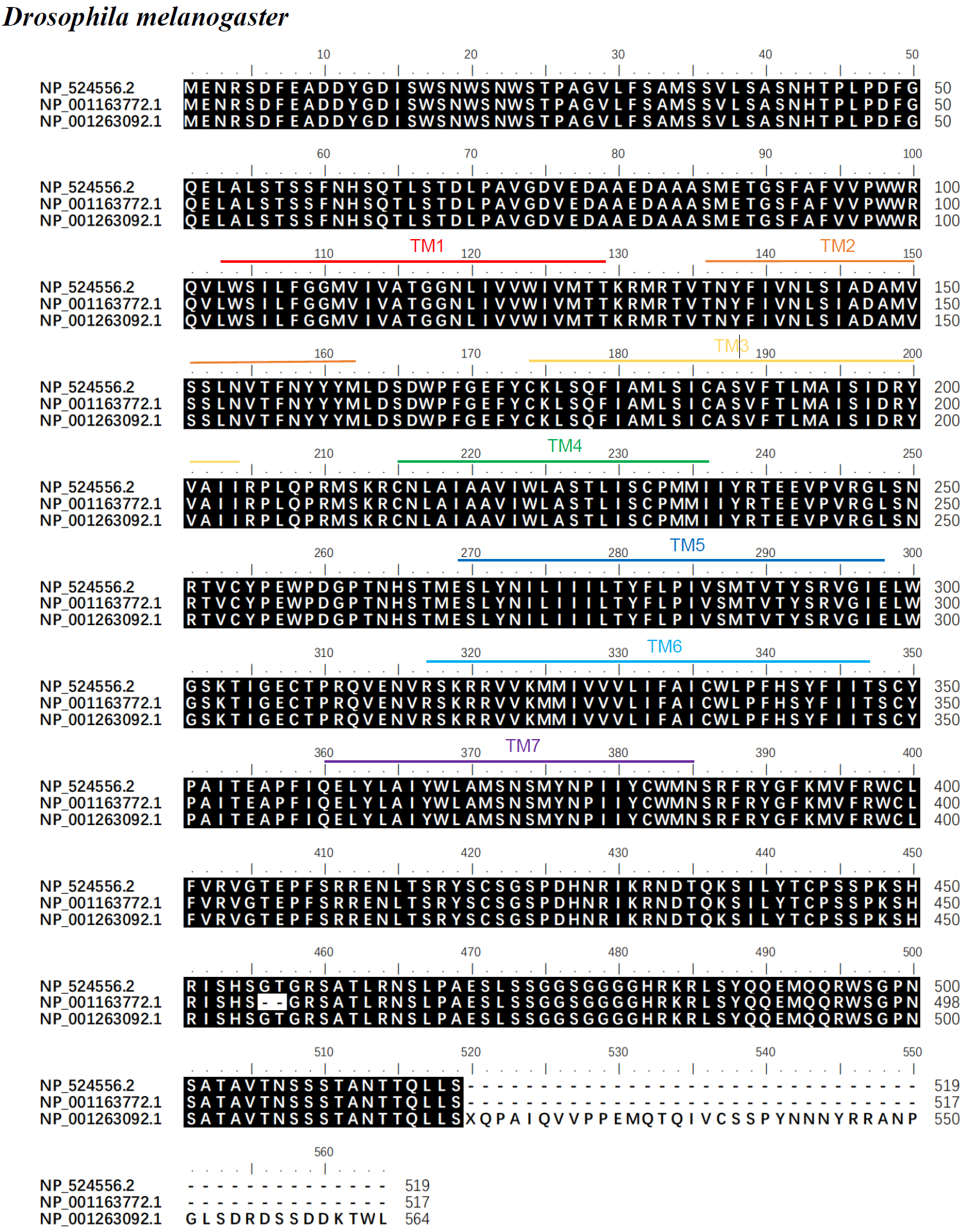


**B**


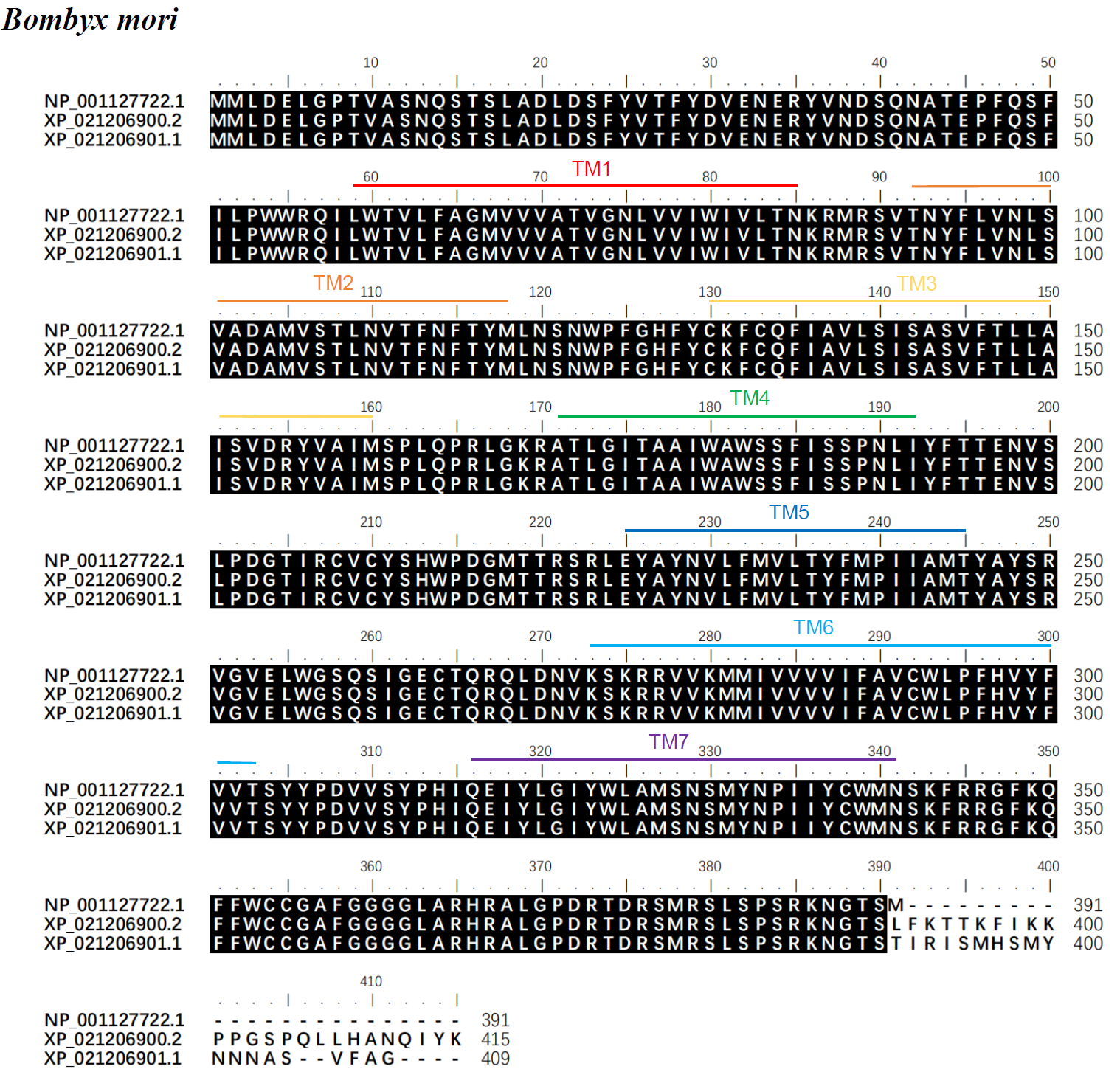


**C**


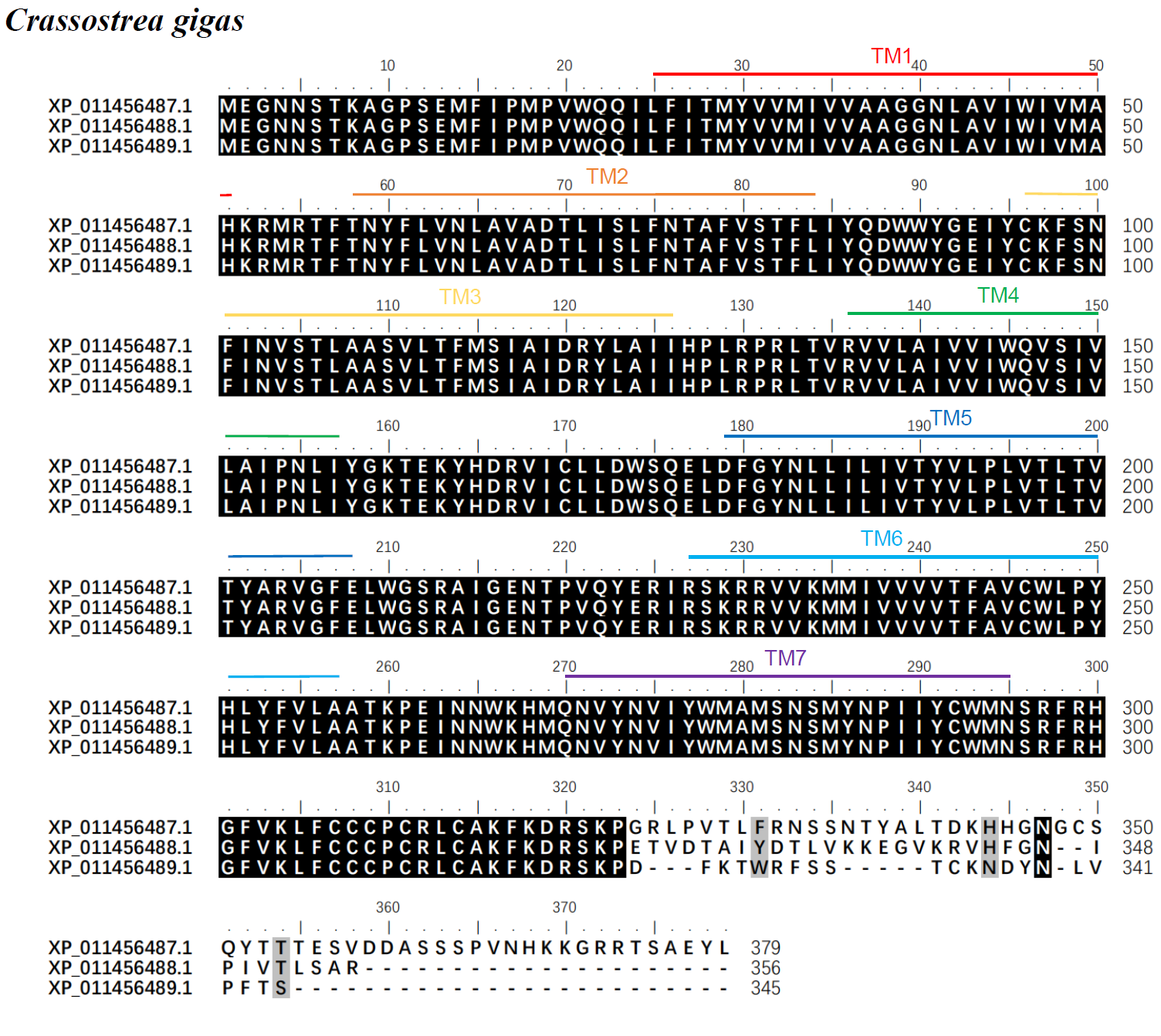


**Figure S7. Receptor isoforms possibly generated from alternative splicing in two arthropod and one molluscan species.** *A*, *Drosophila melanogaster*; *B*, *Bombyx mori*; *C*, *Crassostrea gigas*. They all appear to be C-terminal receptor isoforms in each species.


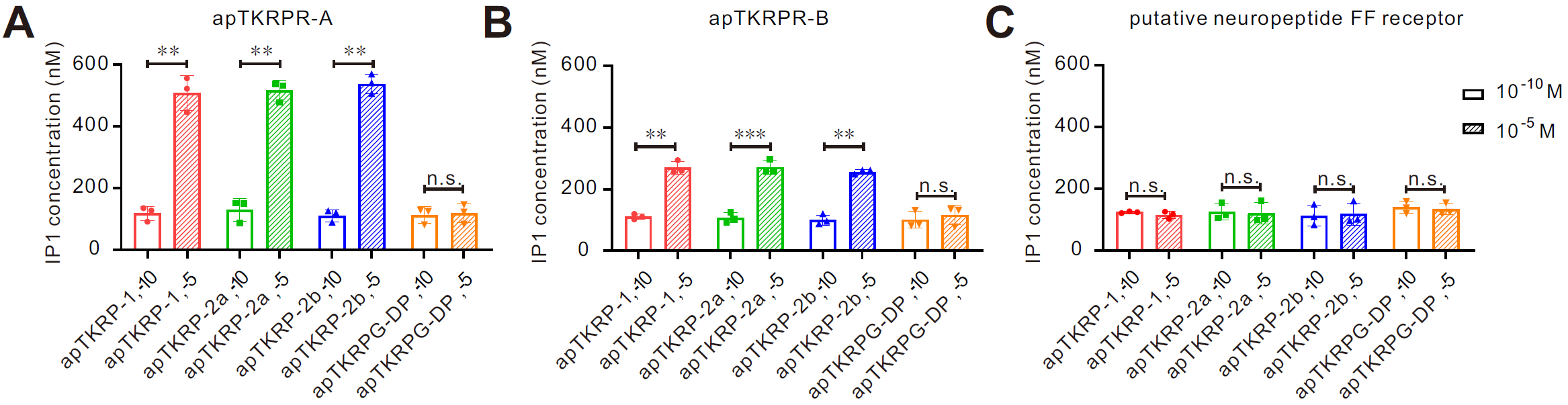


**Figure S8. Screening for potential activation of peptide ligands on putative receptors co-transfected with Gαq (also known as Gα16).** The experiments were similar to those shown in Fig. 6 *D - F*, but included co-transfection with Gαq. Two concentrations of peptides were tested: 10^-10^ M and 10^-5^ M (n = 3). At 10^-10^ M, the peptide minimally activates a receptor, serving as a control. apTKRPs significantly increased IP1 concentration when acting on apTKRPR-A (*A*) and apTKRPR-B (*B*), indicating that apTKRPs are ligands for apTKRPR-A and apTKRPR-B. In contrast, apTKRPG-DP did not activate either apTKRPR-A or apTKRPR-B. Moreover, the putative neuropeptide FF receptor (*C*) did not respond significantly to any of the peptides. Paired two-tailed t-test: n.s., P > 0.05; *, P < 0.05; **, P < 0.01; ***, P < 0.01. Error bars, SD.


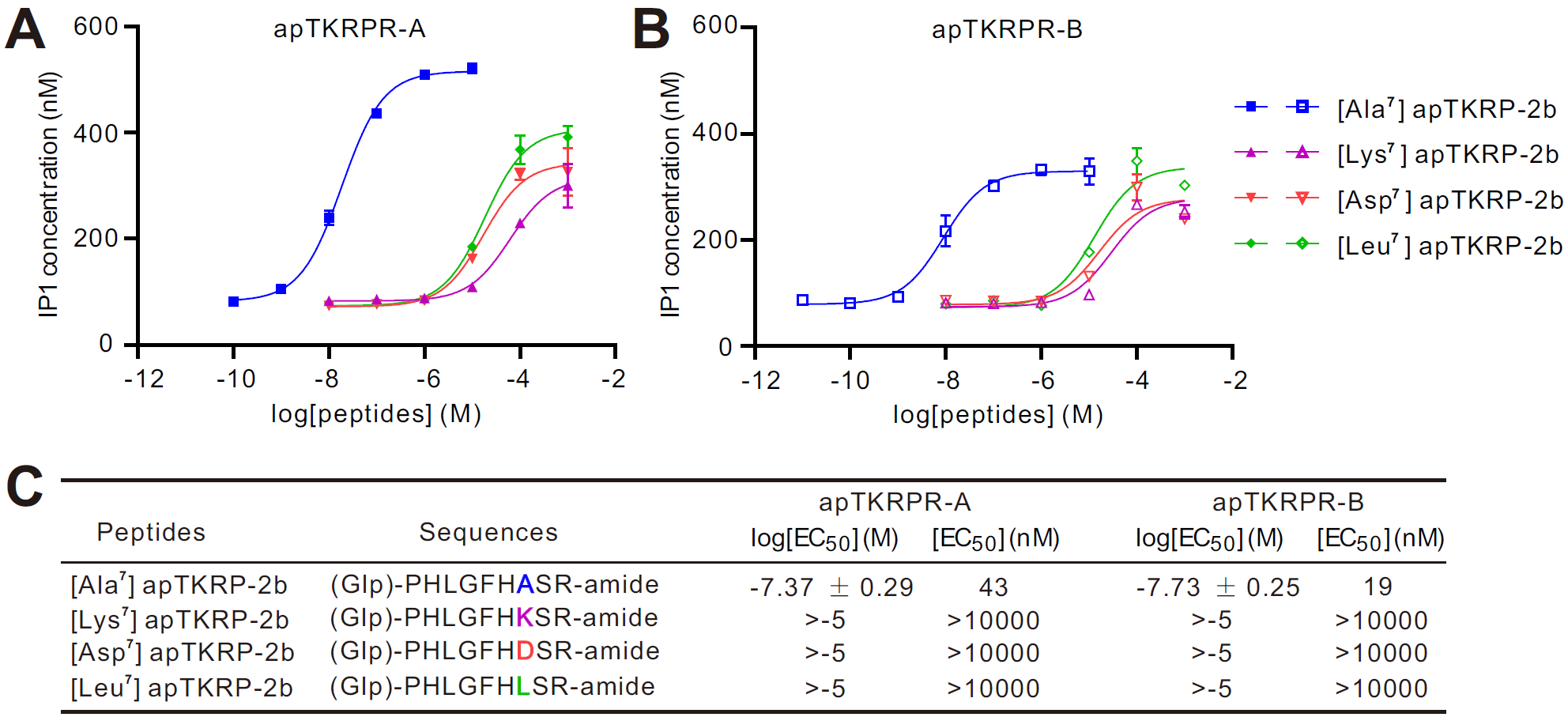


**Figure S9. The role of glycine residue was demonstrated based on the substitution of three additional amino acids.** The three amino acids differed from Gly or Ala (hydrophobic and relatively small) in their different chemical properties: Leu (hydrophobic but larger molecular weight), Asp (negatively charged), Lys (positively charged) *A*, and B, representative examples of dose-response curves showing the ability of apTKRP-2b analogs to activate apTKRPR-A (*A*) or apTKRPR-B (*B*). Error bars, SD. *C*, Sequences of apTKRP-2b analogs and summary of the average log [EC_50_] and EC_50_ on apTKRPRs, log [EC_50_] values are reported as the mean ± SD from at least three independent experiments (apTKRP-2b analogs, n = 4).


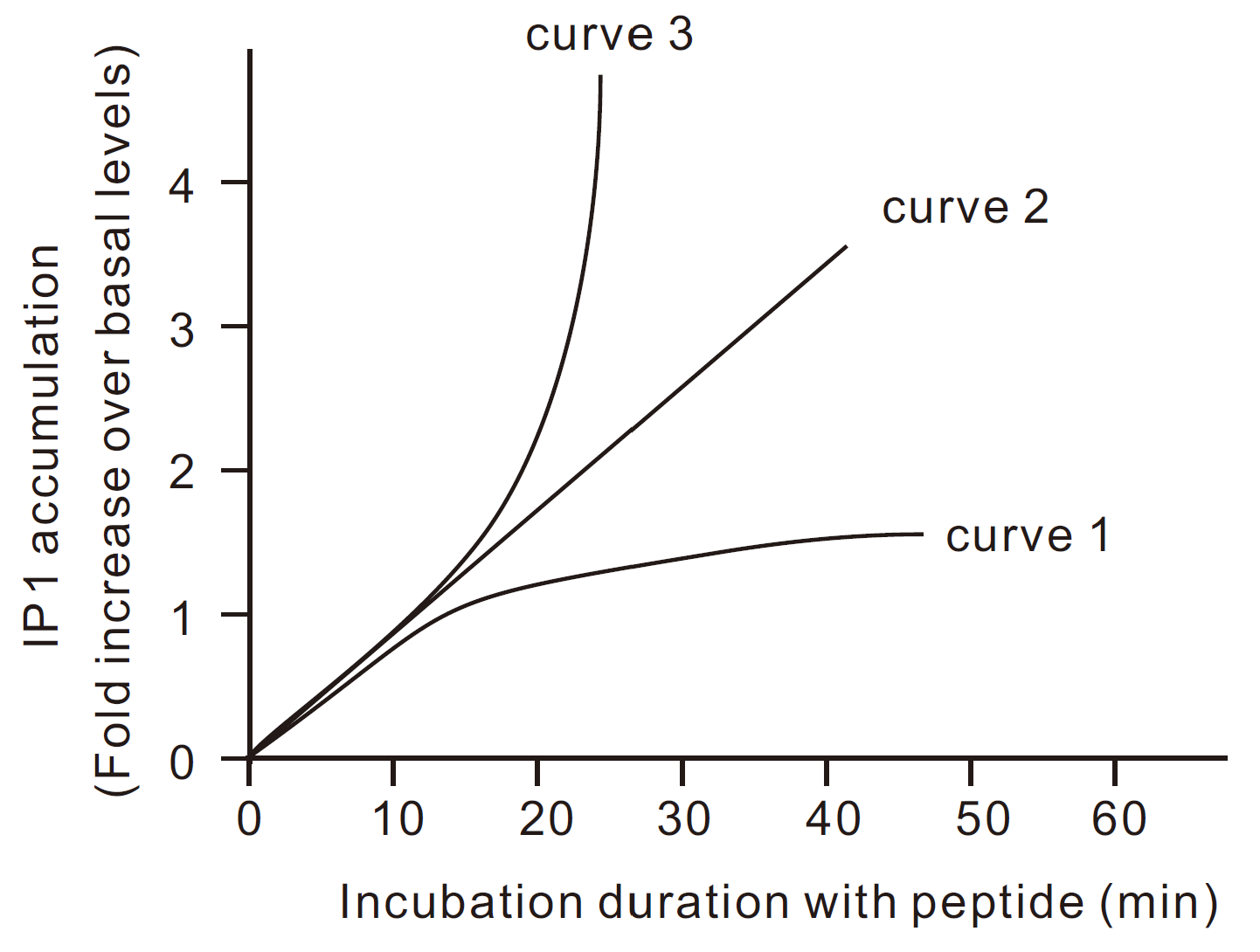


**Figure S10. A schematic diagram of Protocol 1 simulating prolonged exposure of an agonist (apTKRP-2b in this paper).** A response curve was established using the cumulative level of IP1 as the incubation time increases from 2 to 60 min of apTKRP-2b as the X axis. The slope of the curve represents the rate of IP1 accumulation. Curve 1 (responses of a receptor showing desensitization): IP1 accumulation rate gradually slows down; Curve 2 and Curve 3 (responses of a receptor without desensitization): IP1 accumulation rate is constant (curve 2) or gradually increases (curve 3).


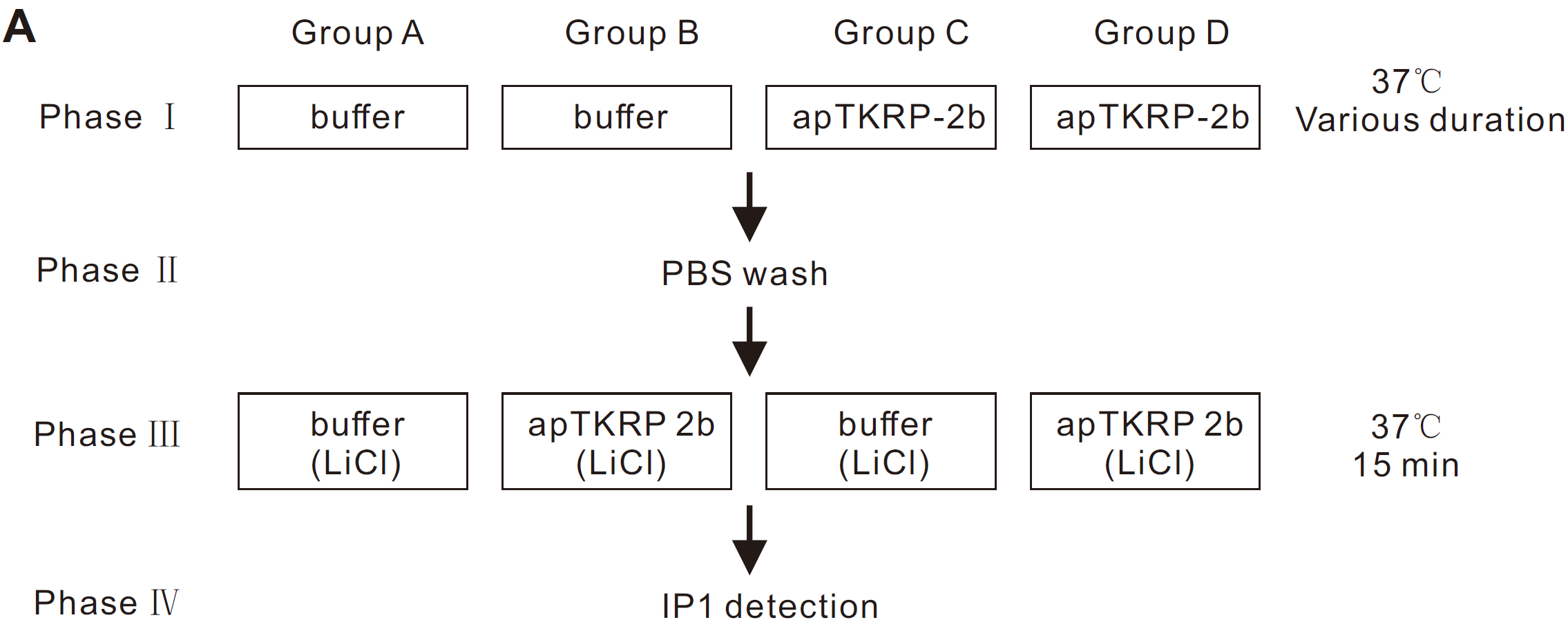


**B**

$$\% \mathrm{Control}=\frac{experimental nM-experimental basal nM}{control nM-control basal nM}\times100$$

control basal nM: IP1 accumulation of Group A;

control nM: IP1 accumulation of Group B;

experimental basal nM: IP1 accumulation of Group C;

experimental nM: IP1 accumulation of Group D.

**Figure S11. A schematic diagram of Protocol 2 simulating repeated (twice) exposure of an agonist (apTKRP-2b in this paper).** *A*, Experimental flow chart. *B*, Data Analysis.


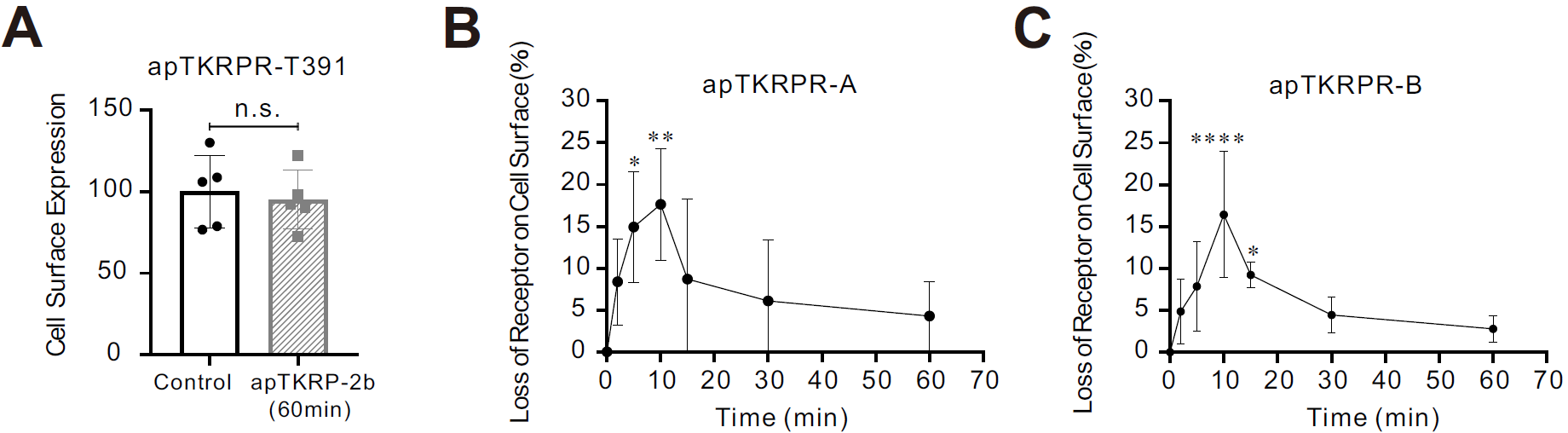


**Figure S12. Cell surface expression of receptors upon exposure of an agonist (apTKRP-2b at 10 µM).** *A*, Receptor expression of a truncated apTKRPR-B, T391, did not change significantly following apRKRP-2b exposure for 60 min (n = 5). Paired T-test. *B* and *C*, Receptor expression of apTKRPR-A (*B*, F(6, 21) = 3.723, P = 0.011, n = 4) and apTKRPR-B (*C*, F(6, 21) = 7.244, P = 0.0003, n = 4) following agonist exposure for different durations. There is a significant loss of receptor expression particularly around exposure duration of 10 min. Bonferroni post-hoc test: comparison with the expression at 0 min, n.s., *P* > 0.05; *, P < 0.05, **, P < 0.01, ****, P < 0.0001. Error bars, SD.


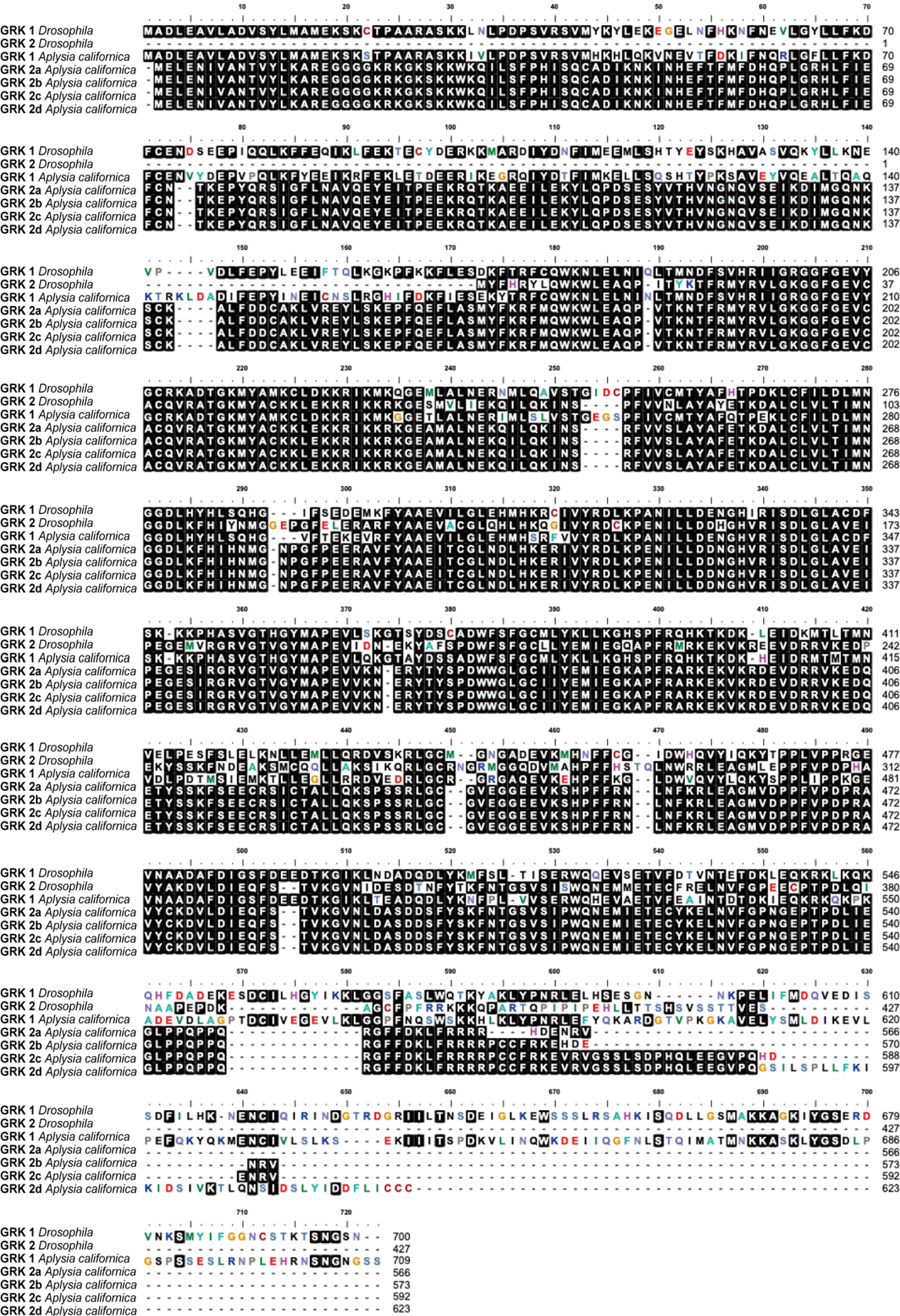


**Figure S13. Comparison of putative GRKs in *Aplysia* with the two GRKs in an arthropod (*Drosophila*) using BioEdit.**


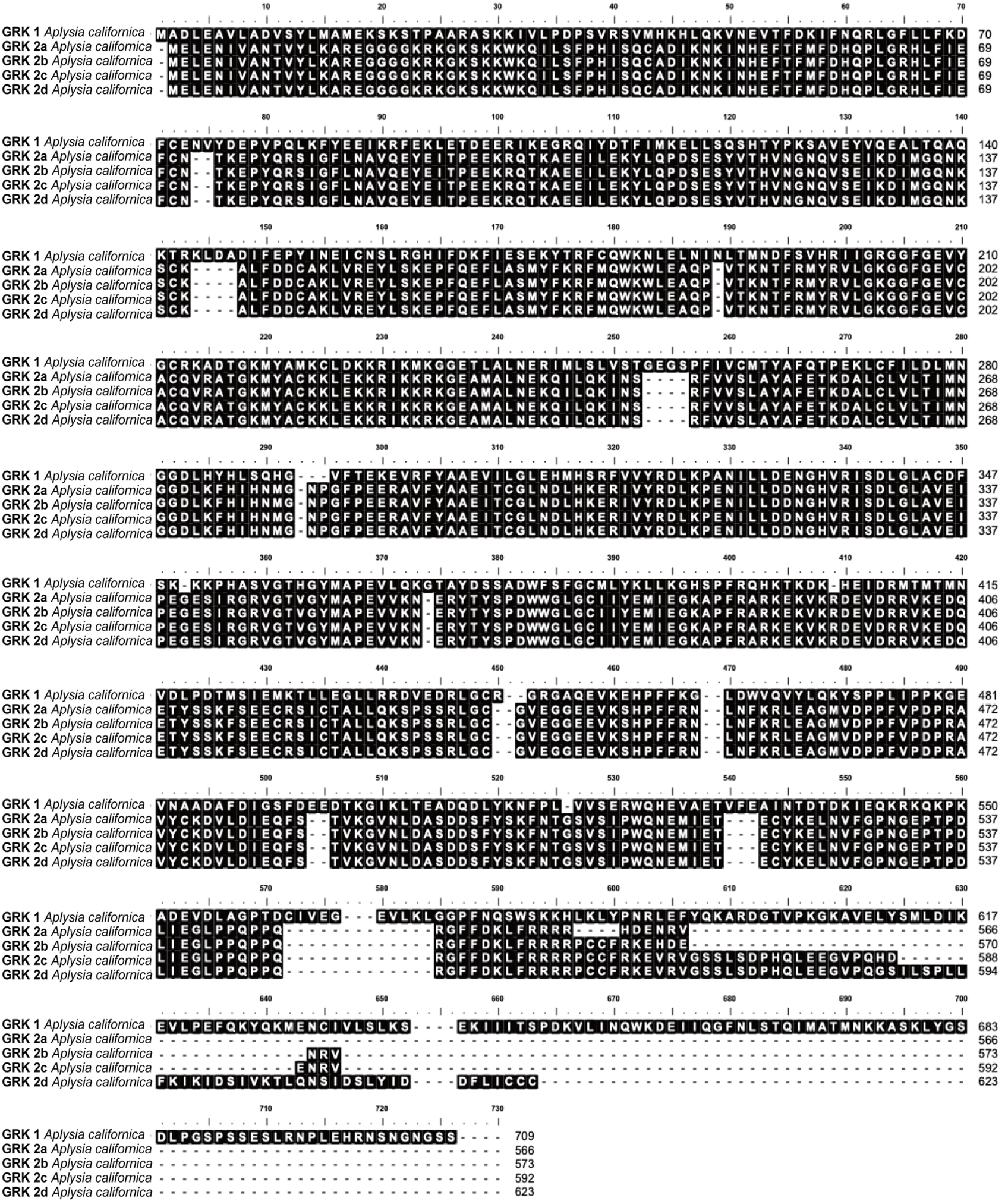


**Figure S14. Comparison of the five putative GRKs in *Aplysia* using BioEdit.**


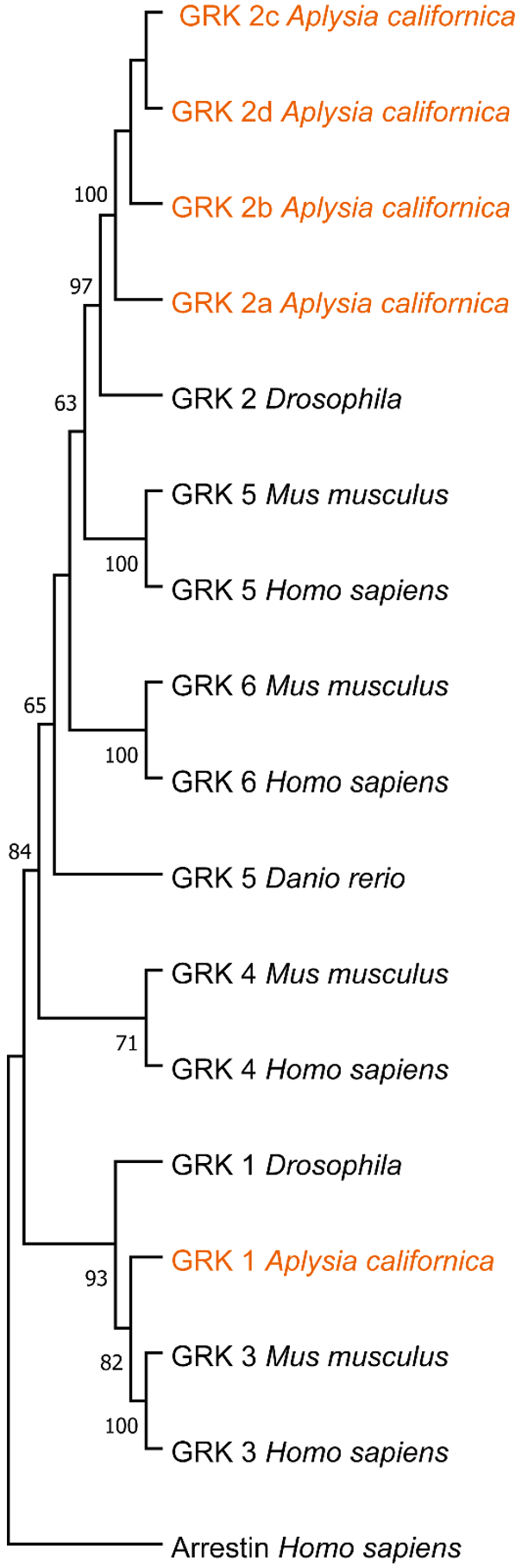


**Figure S15. A phylogenetic tree of predicted and identified GRKs using MEGA X.** Arrestin *Homo sapiens* was used as an outgroup. Numbers at the nodes are bootstrap values as percentages. Only bootstrap values greater than 50 are shown.


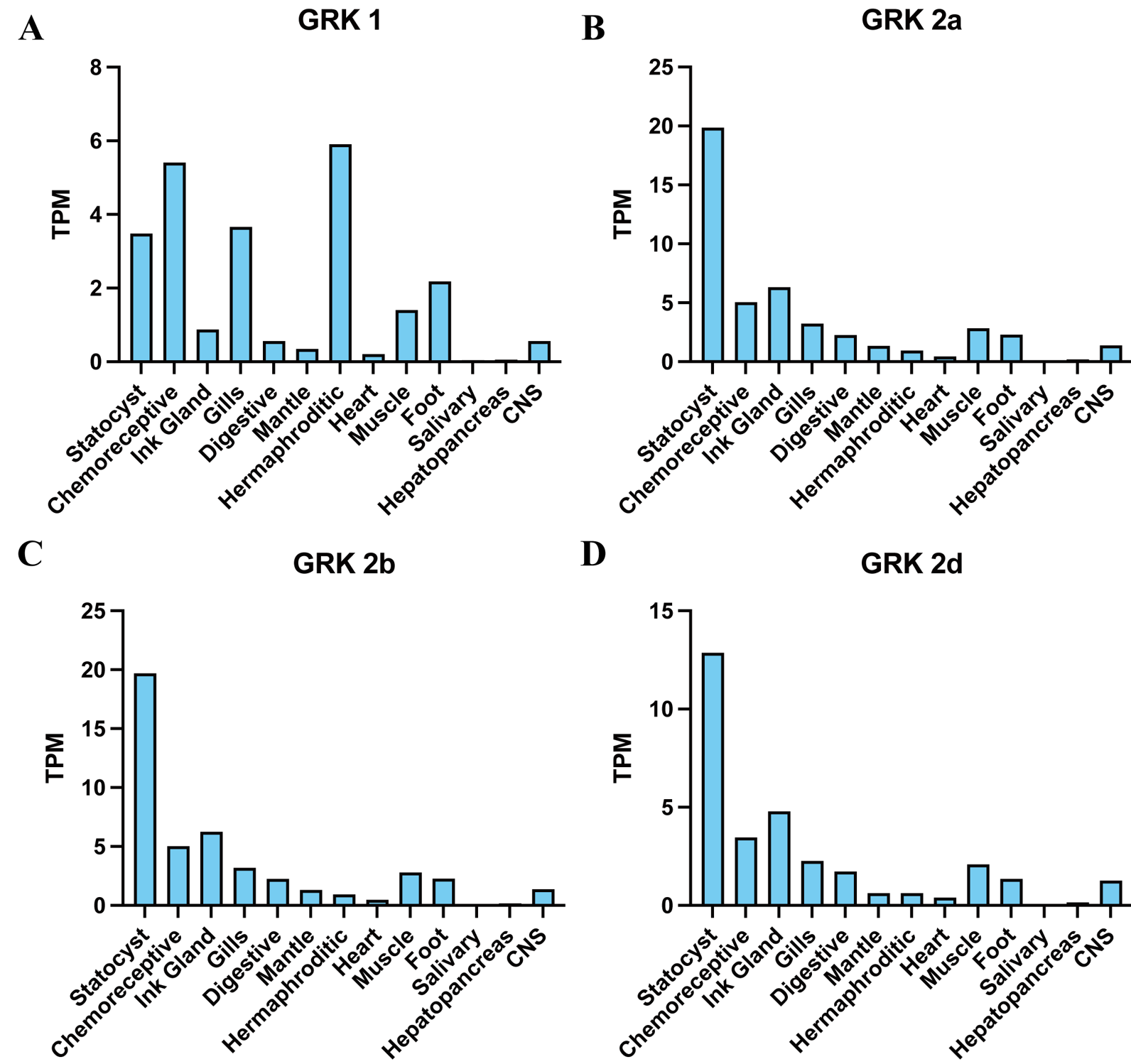


**Figure S16. The expression of GRKs in the CNS and peripheral tissues of *Aplysia***. *A*, GRK 1; *B*, GRK 2a; *C*, GRK 2b; *D*, GRK 2d. The RNA-seq data is represented as TPM (transcript per million).


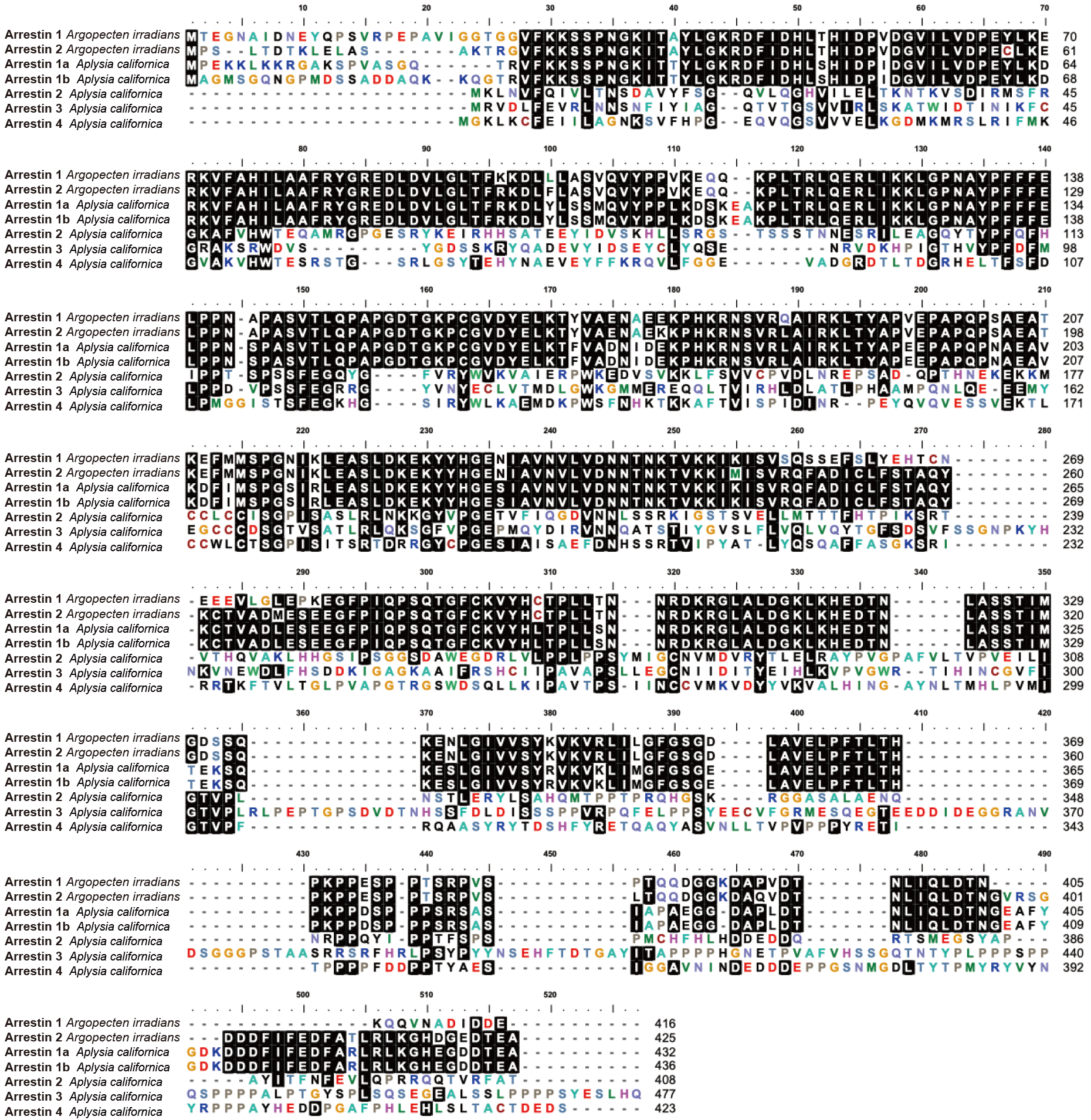


**Figure S17. Comparison of putative arrestins in *Aplysia* with the two arrestins in a mollusk (*Argopecten*) using BioEdit**.


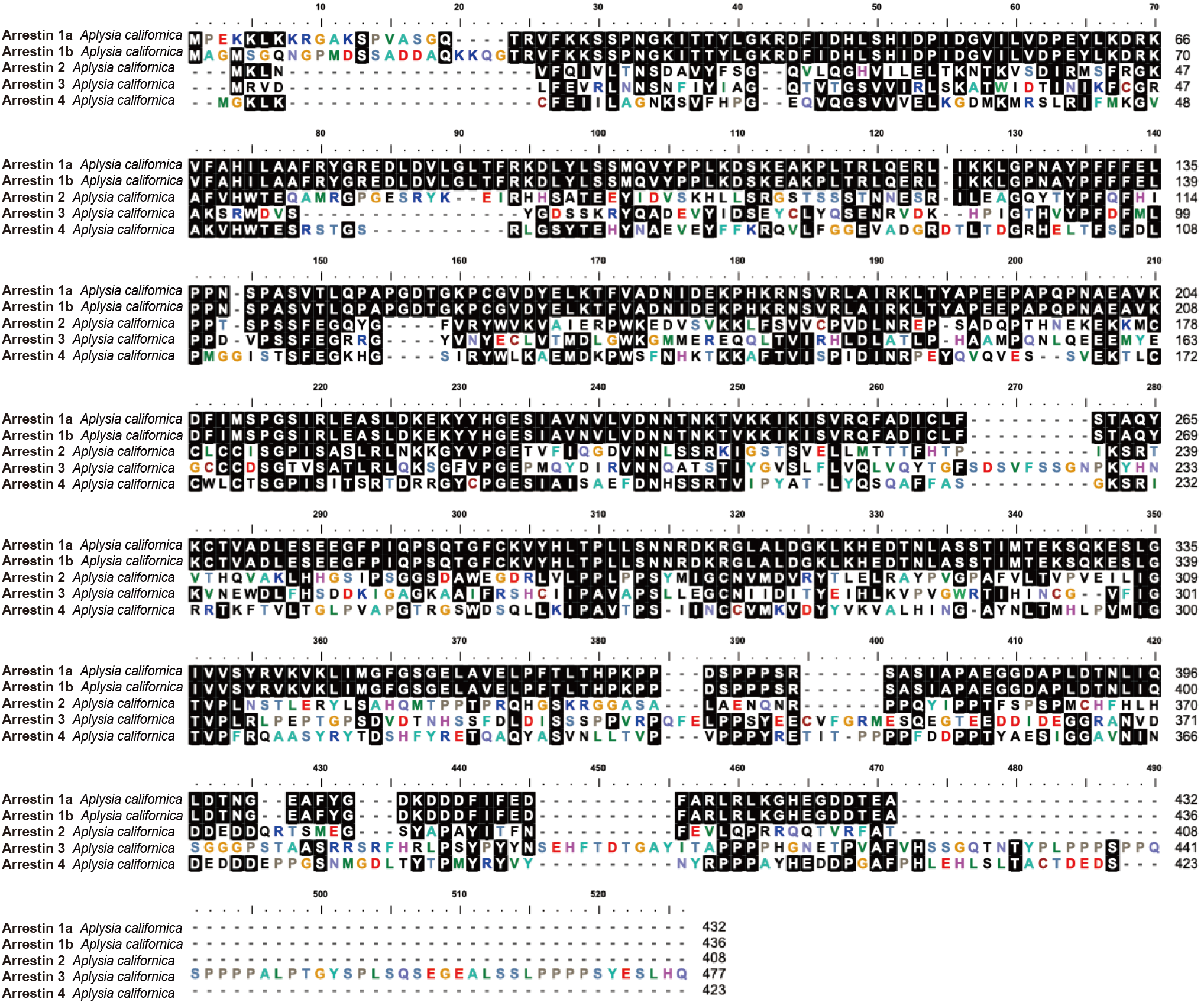


**Figure S18. Comparison of the five putative arrestins in *Aplysia* using BioEdit.**


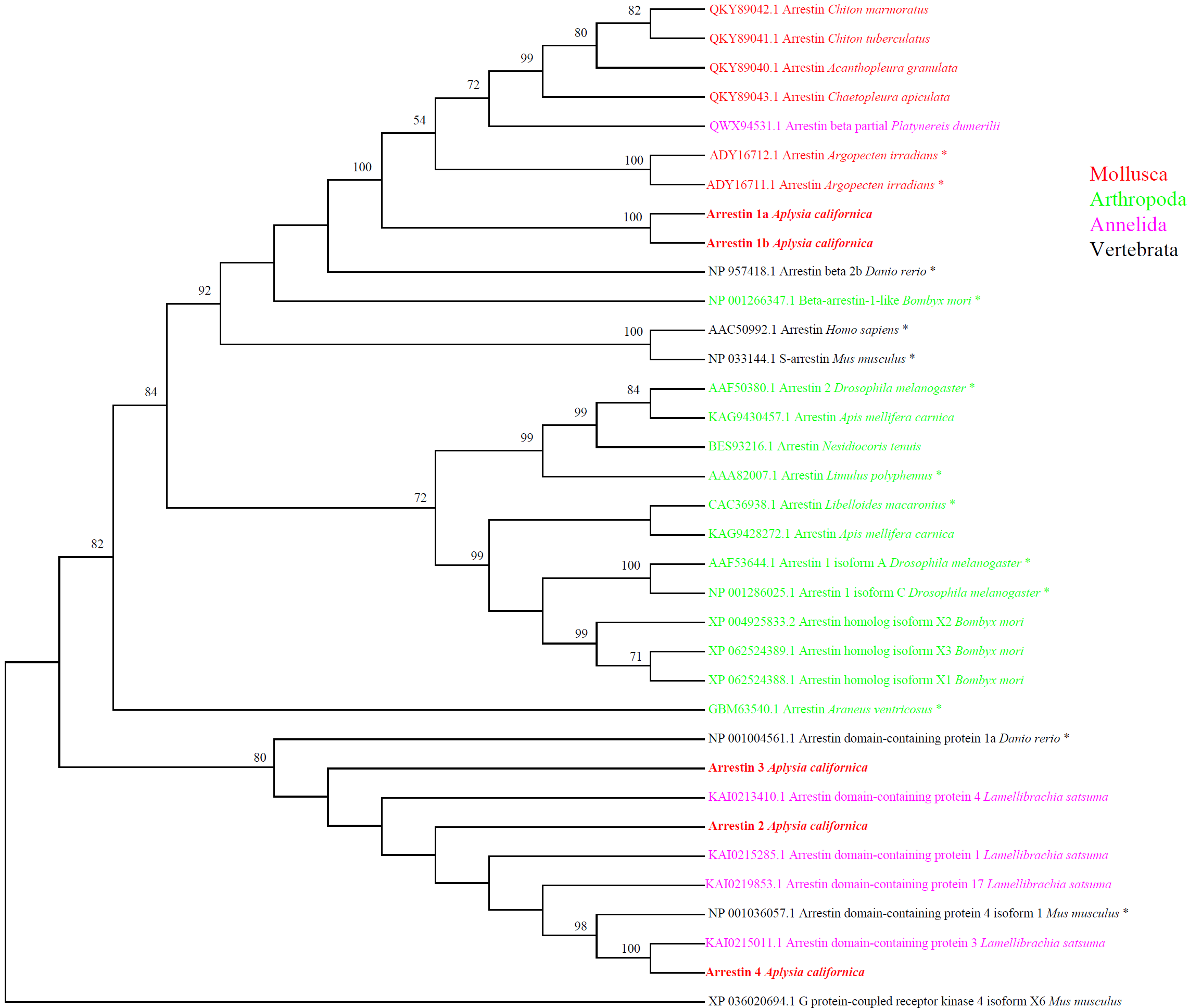


**Figure S19. A phylogenetic tree of predicted and identified GRKs using MEGA X.** GRK 4 *Mus musculus* was used as an outgroup. Numbers at the nodes are bootstrap values as percentages. Only bootstrap values greater than 50 are shown.


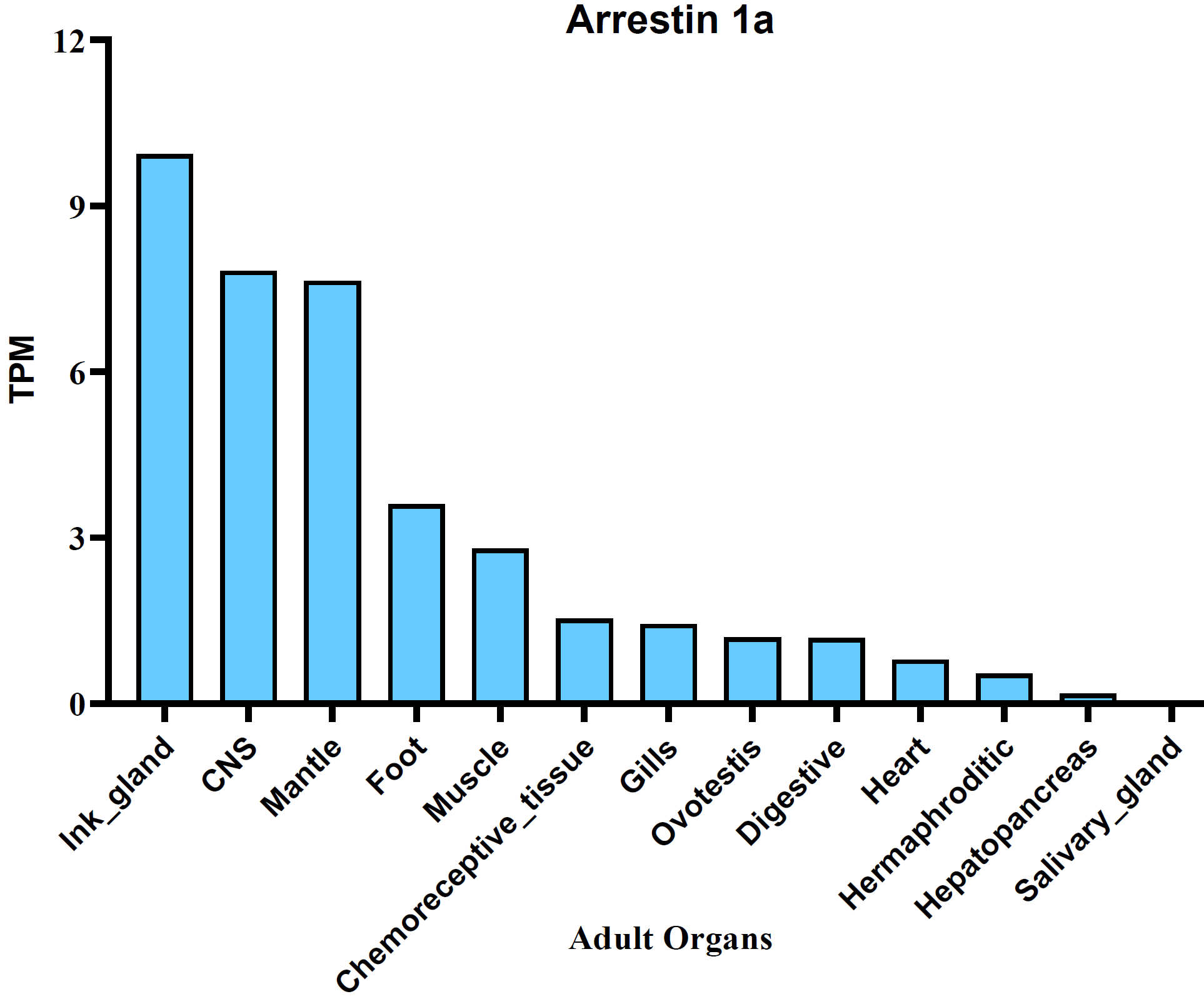


**Figure S20. The expression of arrestin 1a in the CNS and peripheral tissues of *Aplysia***. The RNA-seq data is represented as TPM (transcript per million).
